# Increased carrier mobility in end-functionalized oligosilanes†
†Electronic supplementary information (ESI) available: Synthetic procedures, tabulated characterization data, single crystal X-crystallography, differential scanning calorimetry thermograms, optical and force microscopy, optical and electronic characterization and device fabrication procedures. CCDC 1030984–1030987. For ESI and crystallographic data in CIF or other electronic format see DOI: 10.1039/c4sc03274h


**DOI:** 10.1039/c4sc03274h

**Published:** 2015-01-05

**Authors:** S. Surampudi, M.-L. Yeh, M. A. Siegler, J. F. Martinez Hardigree, T. A. Kasl, H. E. Katz, R. S. Klausen

**Affiliations:** a Department of Chemistry , Johns Hopkins University , 3400 N. Charles St , Baltimore , MD 21218 , USA . Email: klausen@jhu.edu; b Department of Materials Science and Engineering , Johns Hopkins University , 3400 N. Charles St , Baltimore , MD 21218 , USA

## Abstract

We show that a class of oligosilane–arene σ, π-hybrid materials exhibits distinct and enhanced solid-state electronic properties relative to its parent components. In the single crystal structure, the σ-conjugation axis of one molecule points towards the π-face of a neighboring molecule due to an unusual gauche conformation. This organization is hypothesized to be beneficial for charge transport. We show that solution-deposited crystalline films of the hybrid materials show up to a 100-fold increase in space-charge limited current (SCLC) mobility relative to literature reports of photoinduced hole transport in oligosilane films. The discovery that σ, π-hybrids are more than the sum of their parts offers a design opportunity for new materials.

## Abstract 2

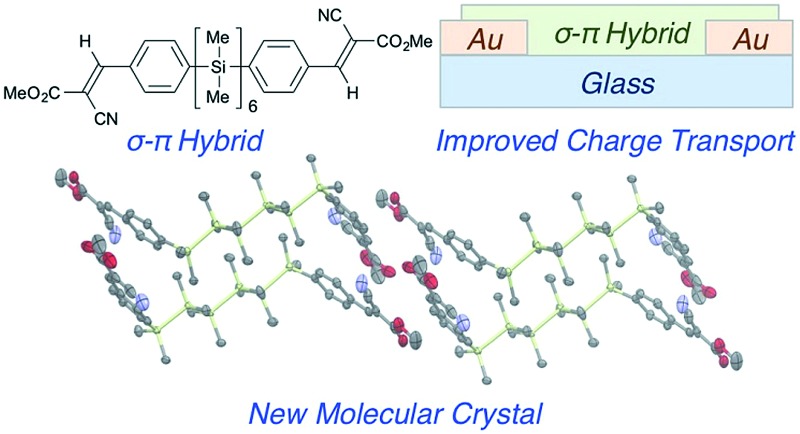
A class of hybrid oligosilane–arene materials outperforms the σ- and π-conjugated parent structures.

## Introduction

The ordering of individual molecules in a solid is as important for efficient charge transport as the electronic properties of the molecule itself. This manuscript describes a new, close-packed molecular crystal form exhibited by oligosilanes terminated with cyanovinyl groups. We report record-setting oligosilane charge carrier mobilities in their crystalline thin films.

**Fig. 1:**
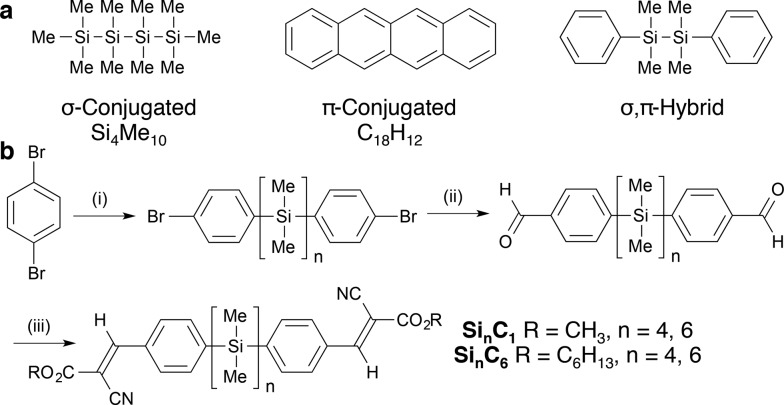
(a) Representative σ-conjugated, π-conjugated and hybrid materials. (b) Synthesis of cyanovinylsilanes. (i) *n*-BuLi, Et_2_O, 0 °C; Cl(SiMe_2_)_*n*_Cl (82–96%). (ii) *n*-BuLi, Et_2_O, 0 °C; DMF, (iii) RO_2_CCH_2_CN, piperdine, benzene, 80 °C (72–89%, 2 steps).

Crystalline silicon is the preeminent semiconductor. While molecular variants of other extended materials are well developed for electronic applications (for example, the pentacene^1–3^ core is a substructure of the graphitic carbon lattice), oligosilanes are not broadly studied as small molecule semiconductors. Nonetheless, oligosilanes have attractive properties that merit consideration. Permethylsilanes (Si_*n*_Me_2*n*+2_, Fig. 1)^4^ are the simplest organosilanes and are bench-stable, processable in organic solvents and absorb light strongly in the ultraviolet region due to delocalized σ molecular orbitals.^5,6^ Molecular silanes retain the practical attractions of silicon itself, like earth abundance and low toxicity. Lastly, the potential for structural variation is enormous. As part of a program dedicated to identifying the structural requirements for facile charge transport in silanes, here we focus on the integration of molecular silicon with functionalized π-conjugated organic groups.

## Results and discussion

### Design and synthesis

Tetra- and hexasilanes were selected for initial study on the basis of structure–property relationships identified in photoinduced hole transport studies of oligosilanes^7^ and polysilanes^8^ (*μ* = 10^–4^ to 10^–3^ cm^2^ V^–1^ s^–1^). Okumoto *et al.* in a time-of-flight study of polycrystalline Si_*n*_Me_2*n*+2_ (*n* = 8–12) films showed that mobility is lower in odd-numbered than even-numbered silane films and that mobility increases with oligosilane length (Si_8_Me_18_, *μ* ≅ 7.5 × 10^–4^ cm^2^ V^–1^ s^–1^).^7^ Kepler *et al.* observed similar magnitude hole mobilities in poly(SiMePh) and poly(Si(*n*-Pr)_2_) films (*μ* = 10^–4^ cm^2^ V^–1^ s^–1^), but poly(SiMePh) devices were more photostable.^8^

To further elucidate substituent effects, we installed terminal functionalized arenes, which also increase crystallinity. We particularly focused on cyanovinyl groups, an archetypal electron acceptor moiety.^9,10^ Thienyloligosilanes and polymers have previously been described.^11,12^ α,ω-Dichlorooligosilanes of defined length were synthesized and then arylated with 4-bromophenyllithium according to Tamao's literature procedure (Fig. 1b).^13,14^ Subsequent lithium–halogen exchange, formylation with *N*,*N*-dimethylformamide, and Knoevenagel condensation with methyl 2-cyanoacetate yields cyanovinylsilanes Si_4_C_1_ and Si_6_C_1_. Use of *n*-hexyl 2-cyanoacetate in the Knoevenagel condensation yields Si_4_C_6_ and Si_6_C_6_. Gratifyingly, all products are crystalline solids at room temperature. Products are exclusively the (*E*)-olefin isomer, as determined by 2-D NMR spectroscopy and single crystal X-ray diffraction (XRD).

### Unusual *gauche*-conformation in crystal structure

**Fig. 2:**
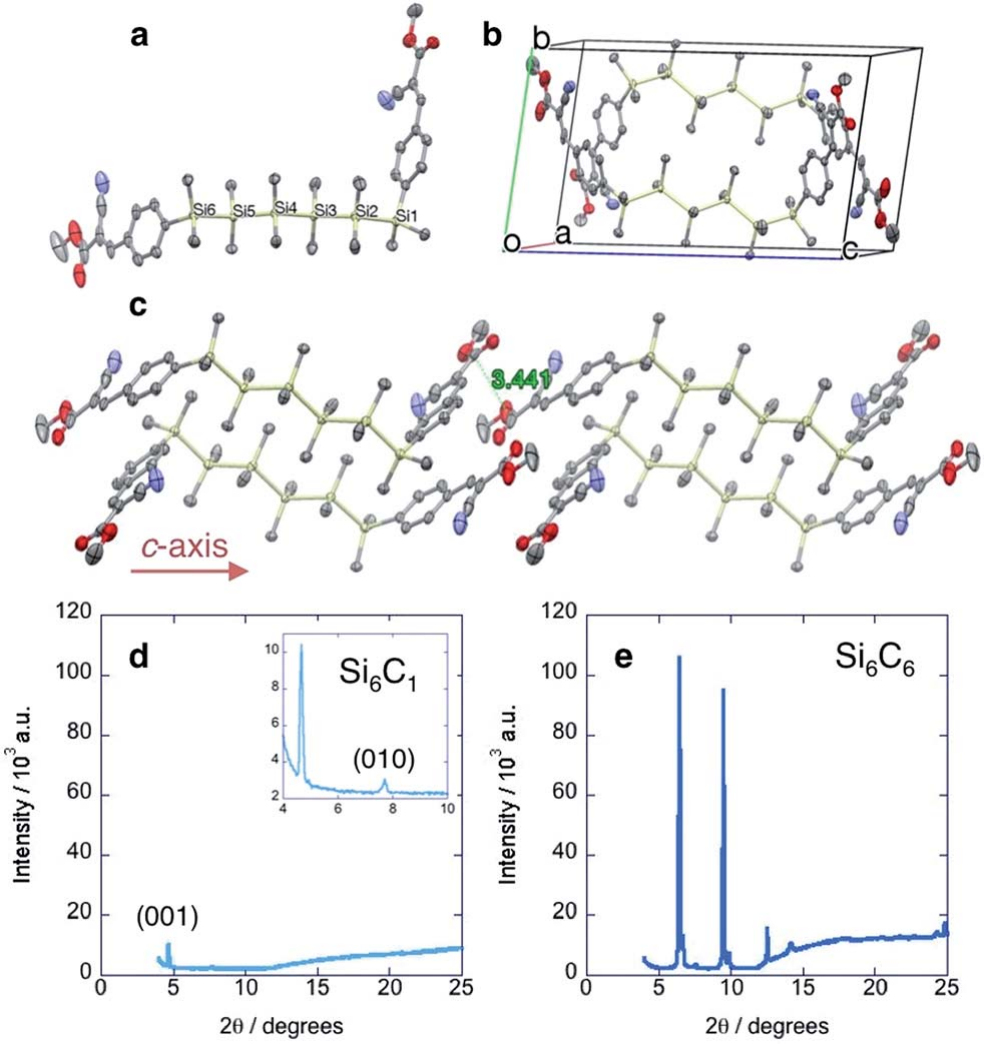
Cyanovinylsilane crystal packing. (a) Birds-eye view of Si_6_C_1_ molecule highlighting the *gauche* conformation at Si_1_. (b) Unit cell of Si_6_C_1_. (c) Crystal packing of Si_6_C_1_ extended along the *c* direction illustrating the short (3.44 Å) intermolecular distance between carbonyl carbons. (d) Powder XRD pattern of Si_6_C_1_ thin film. Inset shows higher resolution image. (e) Powder XRD pattern of Si_6_C_6_ thin film. Cu Kα = 1.54 Å. Displacement ellipsoids are shown at 50% probability. Carbon = gray; silicon = yellow; nitrogen = blue; oxygen = red. Hydrogens omitted for clarity.

The single crystal XRD structures of Si_4_C_1_ and Si_6_C_1_ show that one terminal arene is gauche relative to the silane chain (Fig. 2a), a highly unusual conformation. Most acyclic α,ω-arylmethylsilanes in the Cambridge Crystallographic Database adopt a fully extended, all-*trans* structure and a more limited number have eclipsed conformations.^14,15^ A complete list of references to relevant crystal structures can be found in the ESI.† A single crystal XRD structure of an acyclic permethylsilane has not been reported, but the powder XRD pattern of a vacuum-deposited Si_12_Me_26_ thin film shows a *d*-spacing (*d* = 25.9 Å) consistent with all-*anti*-silanes oriented perpendicular to the substrate.^16^

Two molecules of *gauche*-Si_6_C_1_ pack such that cyanovinylarene groups co-facially localize and the resultant dimer constitutes the unit cell (Fig. 2b). The distance between carbonyl carbons in neighboring dimers along the *c* direction is short (3.44 Å, Fig. 2c). We prepared solution-deposited crystalline films by drop-casting a 1,2-dichlorobenzene oligosilane solution onto a Si/SiO_2_ substrate. The XRD pattern of the Si_6_C_1_ thin film is consistent with a polycrystalline film in which silanes are oriented both perpendicular and parallel to the substrate. The most intense peak in the Si_6_C_1_ powder XRD pattern (Fig. 2d, 2*θ* = 4.67°, *d* = 18.9 Å) is consistent with the *c*-axis of the Si_6_C_1_ unit cell (20.4 Å) and is therefore assigned to reflection from the (001) plane. A lower intensity peak (2*θ* = 7.71°, *d* = 11.5 Å) is assigned to reflection from the (010) plane (*b*-axis = 11.8 Å).

While we were unsuccessful in obtaining a single crystal XRD structure of Si_6_C_6_ or Si_4_C_6_, the thin film XRD patterns are not consistent with a fully extended conformation. The intense reflection at 2*θ* = 6.47° corresponds to a *d*-spacing of 13.7 Å, significantly shorter than the predicted fully extended molecular length of >41.0 Å (see ESI† for more details). Peaks in the Si_6_C_6_ powder XRD pattern are much more intense than in the Si_6_C_1_ film, suggesting that Si_6_C_6_ forms the more well-ordered film with larger domains, a hypothesis supported by optical microscopy and atomic force microscopy (*vide infra*).

### Increased charge carrier transport

**Fig. 3:**
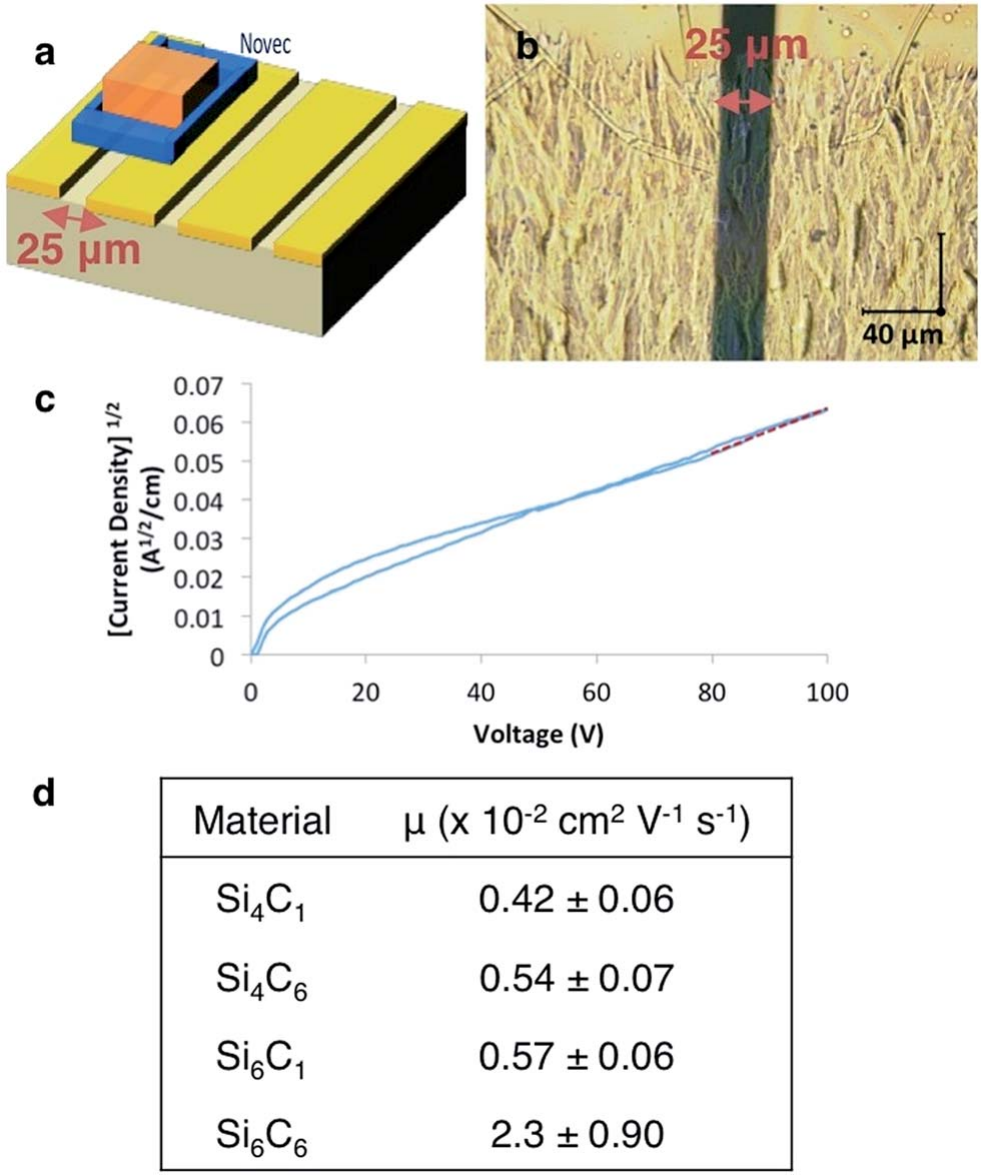
(a) Device architecture. (b) Optical microscope image of Si_6_C_6_ crystalline thin film. (c) Representative *J* ^1/2^–*V* plot of Si_6_C_6_ device. A least squares fit (red line) to the SCLC regime gives *y* = *ax* + *b* (*a* = 5.7 × 10^–4^; *b* = 5.4 × 10^–3^; *R* ^2^ = 0.98). (d) Tabulated SCLC mobility data for cyanovinylsilane materials. Each mobility is the average of at least five devices and error bars represent standard deviations. *J* = current density; *V* = voltage; Novec™ = 3M™ Novec™ 1700 Electronic Grade Coating.

In the Si_6_C_1_ structure, the gauche conformation allows for a close-packed structure and the intermolecular spacing in the *c*-axis direction is short (3.44 Å). Charge transport in the *c*-axis direction could therefore be particularly efficient as the *c*-axis is also aligned with the σ-conjugation axis. We fabricated devices to characterize electronic properties (Fig. 3a). Gold electrodes (50 nm) were thermally evaporated onto clean glass slides with a 25 μm tungsten wire shadow mask. The wires were removed and a rectangular pattern (1 × 2.5 mm) defined on the electrodes with Novec™ 1700 Electronic Grade Coating. A 1,2-dichlorobenzene solution of oligosilane was drop-cast onto the Novec™ pattern, then sequentially dried in ambient conditions and in a vacuum oven. A transparent, crystalline film covers the 25 μm channel (Fig. 3b). Current–voltage characteristics were measured and the charge carrier mobility extracted from the space-charge limited current (SCLC) regime of the *J* ^1/2^–*V* plot using the Mott–Gurney law (Fig. 3c and ESI†).^17^ We observe, as did Okumuto *et al.*,^7^ that the mobility increases with silane length.

More unusually, we observe an increase in mobility with increasing the length of the ester alkyl chain (Si_6_C_1_ < Si_6_C_6_ and Si_4_C_1_ < Si_4_C_6_, Fig. 3d). This is in distinct contrast to Okumoto's study showing a decrease in mobility in *anti*-silane films with longer alkyl groups due to increased interlayer distances.^18^ That we observe the opposite trend is supportive of the hypothesized beneficial effect of the *gauche*-conformation.

In solution, Si_6_C_1_ and Si_6_C_6_ have identical optical and electronic properties. The dependence of device mobility on alkyl chain length is therefore attributed to the increased crystallinity of the thin film as detected by XRD analysis (Fig. 2d and e).

**Fig. 4:**
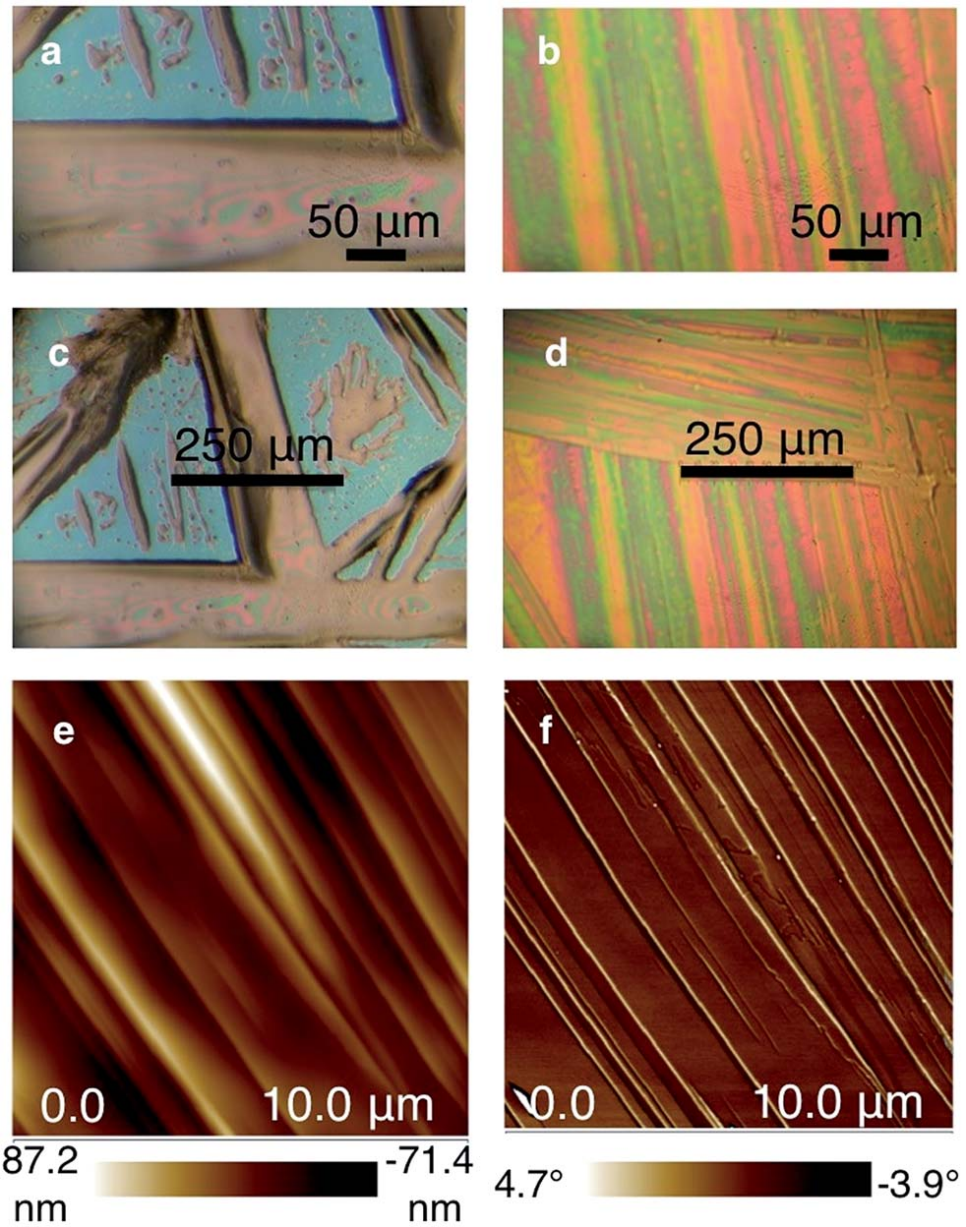
(a and c) Optical microscope image of drop-cast Si_6_C_1_ thin film on Si/SiO_2_ showing discontinuous structure and large needles. Images are at different resolutions. (b and d) Optical microscope image of drop-cast Si_6_C_6_ thin film on Si/SiO_2_ showing continuous and highly oriented film structure. Images are at different resolutions. (e) AFM height image of Si_6_C_6_ thin film. (f) AFM phase image of Si_6_C_6_ thin film. Both the height and phase images show oriented smooth regions. The images are consistent with a lamellar sheet morphology.

Optical and atomic force microscopy images confirm the superior film structure. The drop cast Si_6_C_1_ film contains both relatively large (100–150 μm wide) needles and precipitated islands (Fig. 4a and c). In contrast, the optical microscope image of the Si_6_C_6_ thin film shows a highly oriented smooth and continuous polycrystalline film (Fig. 4b and d). The Si_6_C_6_ thin film was further characterized by atomic force microscopy (AFM) (Fig. 4e and f). Both the AFM height and phase images show oriented, smooth regions and are consistent with a lamellar sheet morphology. Additional images are available in the ESI.†

The combination of silane length and desirable film morphology makes the Si_6_C_6_ SCLC mobility (*μ* = 0.023 cm^2^ V^–1^ s^–1^) the highest mobility measured in this system or in any molecular silane. High carrier mobility is also observed in preliminary thin film transistor studies. Three devices with a Si_6_C_6_ active layer and width-to-length ratios between 10 and 20 showed transistor-like output curves, from which mobilities between 0.01 and 0.06 cm^2^ V^–1^ s^–1^ were calculated, consistent with the SCLC values (see ESI†).

The carrier mobilities in this study are 1–2 orders of magnitude greater than previous reports, an especially notable result given that our oligosilanes (*n* = 4–6) are shorter than those previously characterized (*n* ≥ 8) and mobility increases with silane length. The observed carrier mobilities likely represent the lower limit of the carrier mobility because of the polycrystalline nature of the thin films investigated and the hypothesized anisotropy in charge transport.

### Alkyl-linked cyanovinylarenes are less effective charge transport materials

**Fig. 5:**
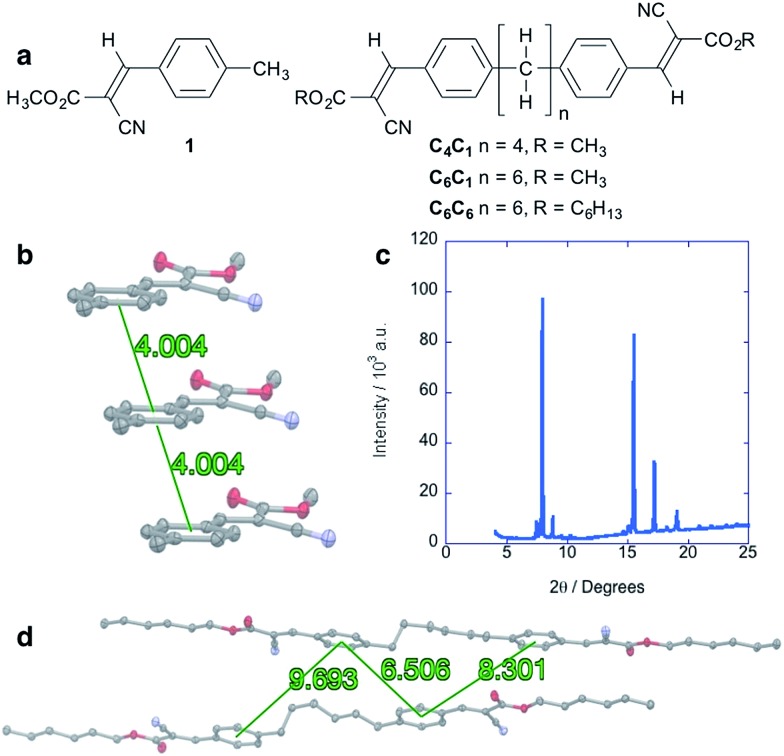
(a) Chemical structure of alkyl cyanovinyl materials. (b) Crystal packing of **1** along the *a* direction highlighting slip-stacked structure. Distances are given for arene centroid to centroid. (c) Powder XRD diffraction pattern of solution-deposited thin film of **1**. Cu Kα = 1.54 Å. (d) Structure of two neighboring molecules of C_6_C_6_. Distances are given for arene centroid to centroid. Displacement ellipsoids are shown at 50% probability. Carbon = gray; nitrogen = blue; oxygen = red. Hydrogens are omitted for clarity.

Alkane control molecules (Fig. 5a) strongly point to an essential structural and electronic role for the silane moieties in the hybrid materials. We find that the parent arene **1** adopts a slip-stacked structure in which centroids are separated by 4.00 Å (Fig. 5b), a very different packing structure from *gauche*-silanes. XRD analysis of a solution-deposited thin film of **1** shows high intensity, narrow peaks consistent with a polycrystalline structure (Fig. 5c). However, electronic characterization of devices fabricated in the same manner as the cyanovinylsilanes did not show a SCLC region and charge carrier mobility could not be determined (see the ESI† for more details). C_6_C_6_ (Fig. 5a), an analog of Si_6_C_6_, was synthesized and characterized as well. Widely displaced and separated aryl rings are observed in the C_6_C_6_ crystal structure (Fig. 5d). Current–voltage characterization does not show a SCLC region in devices prepared from C_4_C_1_, C_6_C_1_, nor C_6_C_6_.†

### Density functional theory calculations

**Fig. 6:**
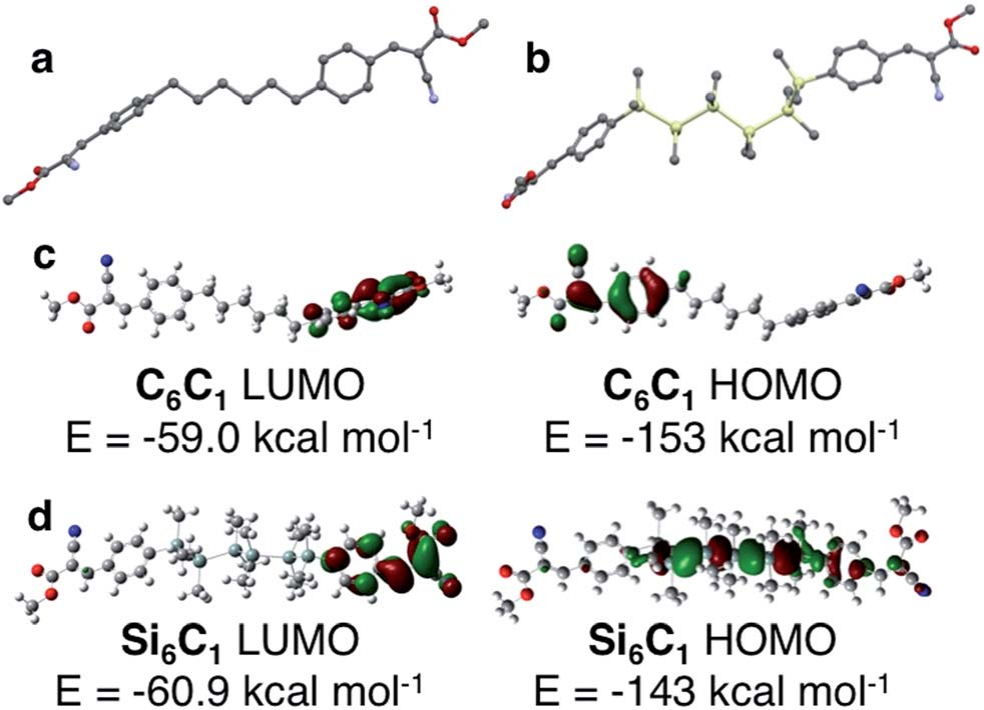
(a) DFT optimized ground state structure of C_6_C_1_ (B3LYP/6-31G). All-*anti* alkane linker is highlighted. Carbon = gray; nitrogen = blue; oxygen = red. Hydrogens omitted for clarity. (b) One of several low energy structures calculated for Si_6_C_1_. The torsional angle defined by Si_1_–Si_2_–Si_3_–Si_4_ is 57.7°, a *gauche* relationship. Carbon = gray; silicon = yellow; nitrogen = blue; oxygen = red. Hydrogens omitted for clarity. (c) DFT calculated highest occupied and lowest unoccupied molecular orbitals of C_6_C_1_. Orbital density is localized on opposite arenes. Carbon = gray; blue = nitrogen; red = oxygen; white = hydrogen. (d) DFT calculated highest occupied and lowest unoccupied molecular orbitals of Si_6_C_1_. The LUMO is localized on one arene, while the HOMO is delocalized on the hexasilane and partially onto one arene. Carbon = gray; pale green = silicon; blue = nitrogen; red = oxygen; white = hydrogen.

The structural role of the silane was further investigated by quantum chemical calculations (B3LYP/6-31G) of C_6_C_1_ and Si_6_C_1_. The optimized gas phase structure of an isolated C_6_C_1_ molecule is different from its crystal structure conformation (Fig. 6a). In the gas phase, linker chains adopt an all-*anti* conformation. The terminal arenes are in line with the linker axis, but the arene planes are perpendicular. Clearly, the kink in the alkane linker observed in the C_6_C_6_ crystal structure is supported by intermolecular interactions. Intermolecular CH–π interactions involving the alkane linker are found in the C_6_C_6_ crystal structure and are illustrated in Fig. S3 (see the ESI† for more details).

Quantum chemical calculations of Si_6_C_1_ are computationally intensive because of the six heavy elements and the additional degrees of freedom introduced by the silane methyl substituents. The calculations have difficulty converging on a single optimized geometry. One low energy conformation (Fig. 6b) has a *gauche* conformation within the silane chain itself (*ω* = 57.7°), a conformation different from both the Si_6_C_1_ crystal structure and the all-*anti* C_6_C_1_ optimized geometry. Other low energy conformations of Si_6_C_1_ have an all-*anti* relationship. We interpret the computational data to mean that rotation about the Si–Si bond in the gas phase is facile. We suggest that in the bulk material, the flexible oligosilane chain can easily adjust to accommodate stabilizing interactions with neighbouring functional groups, which drive the preference for the *gauche*-cyanovinylsilane conformation in the crystal structure. Fig. S4† highlights some of the intermolecular short contacts between Si_6_C_1_ molecules in the unit cell.

Our results are consistent with the body of theoretical studies on oligosilane structure that show a flat conformational profile.^4,19,20^ Studies by West and others report that both gauche and anti conformations are energy minima and differ by <1 kcal mol^–1^. The calculated barrier to rotation is small (∼1 kcal mol^–1^).^19^

Because of the well-known dependence of oligosilane electronic structure on conformation,^21,22^ it is difficult to compute a reliable Si_6_C_1_ electronic structure for comparison with C_6_C_1_ in view of the similar energies of multiple Si_6_C_1_ conformations. Nevertheless, we present one possible pairwise comparison here. In C_6_C_1_, the HOMO and LUMO are localized on opposite arenes and no orbital density is found on the alkane chain (Fig. 6c). In *gauche*-Si_6_C_1_, we find that the LUMO is also localized on one arene, while the HOMO is delocalized across the hexasilane (Fig. 6d). The alternating nodal pattern reproduces the electronic structure of simple permethyl and perhydrohexasilanes.^4,23^

The difference in the Si_6_C_1_ and C_6_C_1_ HOMO energies supports an electronic role for silicon. This is also strongly suggested by a visual comparison of the alkyl and silyl materials: the silanes are bright yellow in color, while the alkyl materials are white. A donor–acceptor interaction between the silane and the cyanovinyl group is consistent with intermolecular charge transfer from cyclosilanes to tetracyanoethylene identified by EPR studies.^24,25^ Intramolecular charge transfer studies have focused on the use of silanes as the bridge in donor-bridge–acceptor systems^26^ and nonlinear optical effects in these systems have been characterized.^27^

## Conclusions

We report here that the combination of σ- and π-conjugated units is greater than the sum of its parts. We observe electronic and structural properties in hybrid materials that cannot be achieved by either unit alone. This observation is expected to be of general interest to the electronic materials community. Our results indicate that further structural optimization of molecular silicon and hybrid materials could yield even greater advances in electronic properties.
