# Supplementary material for: Increased carrier mobility in end-functionalized oligosilanes
Source: Chem Sci. 2015 Jan 5;6(3):1905–9. doi: 10.1039/c4sc03274h (PMC5501097; doi:10.1039/c4sc03274h)

Electronic Supplementary Material (ESI) for Chemical Science.
This journal is © The Royal Society of Chemistry 2015

Increased Carrier Mobility in End-Functionalized Oligosilanes

Sravan Surampudi,[1] Ming-Ling Yeh,[1,2] Maxime A. Siegler,[1] Josue F. Martinez Hardigree, Tyler A. Kasl,[1] Howard E. Katz,[1,2] Rebekka S. Klausen[1*]

[1]*Department of Chemistry, Johns Hopkins University, Baltimore, MD 21218*

[2]*Department of Materials Science and Engineering, Johns Hopkins University, Baltimore, MD 21218*

**klausen@jhu.edu*

Electronic Supplementary Information

Table of Contents

1. General Information	S3
2. Synthetic Procedures	S5
3. UV-Vis Absorbance Spectra	S19
4. Cyclic Voltammetry	S20
5. Single Crystal X-Ray Diffraction	S22
6. Thin Film X-Ray Diffraction	S27
7. Optical Microscopy and AFM	S30
8. Differential Scanning Calorimetry (DSC) Thermograms	S32
9. Space-Charge Limited Current (SCLC) Devices	S34
10. Thin Film Transistor Devices	S39
11. Computational Methods and Coordinates	S43
12. References	S47
13. NMR Spectra of Unknown Compounds	S48

1. General Information

All reactions were performed in oven-dried glassware (oven temperature: 170 °C). The flasks were fitted with rubber septa and reactions were conducted under a positive pressure of argon, unless otherwise noted. Anhydrous anaerobic solvents were obtained from JC Meyer solvent purification system (Model: Phoenix 5 solvent freestanding double columns 24 x 24). All column chromatography was performed on a Teledyne ISCO Combiflash Rf using Redisep RF silica columns.

Materials: Commercial reagents were used without further purification. Commercial reagents purchased from Sigma Aldrich include: 1,4-dibromobenzene (**S4**), lithium, *n*-butyllithium (2.5 M in hexanes), *t*-butyllithium (1.7 M in pentane), aluminum chloride, methyl 2-cyanoacetate (**S7a**), 1,4-dibromobutane (**S8a**), cyanoacetic acid, 1-hexanol, piperidine, ferrocene, triethylsilane, bromobenzene, adipoylchloride, dimethylformamide (anhydrous) and benzene (anhydrous). Commercial reagents purchased from Acros Organics include: *p*-toulaldehyde, chloro(dimethyl)phenylsilane (**S1**), isopropylmagnesium chloride (2 M in THF). Commercial reagents purchased from Tokyo Chemical Industry (TCI) include: 1,2-dichlorotetramethyldisilane. Deuterated chloroform ($CDCl_3$) and deuterated benzene (C_6D_6) were obtained from Cambridge Isotope Laboratories. The following compounds were prepared according to literature procedures: 1,4-dichlorooctamethyltetrasilane (**S3a**),[1] methyl 2-cyano-3-phenylacetate,[2] methyl 2-cyano-3-(*p*-tolyl)acrylate (**1**),[3] hexyl 2-cyanoacetate (**S7b**),[4] 1,6-bis(4-bromophenyl)hexane (**S9b**).[5]

Tungsten wire (25 and 75 μm diameter, 99.95%, metals basis) was purchased from Alfa Aesar. Anhydrous chlorobenzene, 1,2-dichlorobenzene, and toluene were purchased from Sigma-Aldrich and used without purification. Novec™ 1700 electronic grade coating was

purchased from 3M. Si/SiO_2 substrates were made from heavily-doped silicon wafers (Si Tech) with 3000Å thermal oxide (Process Specialties) on top.

Instrumentation: Proton nuclear magnetic resonance (^1H NMR) spectra, carbon nuclear magnetic resonance (^{13}C) spectra and silicon nuclear magnetic resonance (^{29}Si) spectra were recorded on Bruker Avance 400 (400 MHz) spectrometer. Chemical shifts for protons, carbon and silicon are reported in parts per million downfield from tetramethylsilane and are referenced to residual protium in the NMR solvent for ^1H NMR ($CHCl_3$: δ 7.26; C_6H_6: δ 7.16), carbon resonances of the solvent for ^{13}C NMR ($CDCl_3$: δ 77.0; C_6D_6: δ 128.5) and silicon resonance of tetramethylsilane for ^{29}Si NMR (TMS: δ 0.0). Data are represented as follows: chemical shift, multiplicity (br = broad, s = singlet, d = doublet, t = triplet, q = quartet, m = multiplet), coupling constants in Hz, and integration. High-resolution mass spectra were obtained at the Johns Hopkins University Mass Spectrometry Facility using a VG Instruments VG70S/E magnetic sector mass spectrometer with EI (70 eV). Elemental analysis was performed at Robertson Microlit Laboratories, New Jersey. UV-Visible (UV-Vis) absorption spectra were obtained on a Varian Cary50 Bio UV-Visible spectrophotometer.

2. Synthetic Procedures

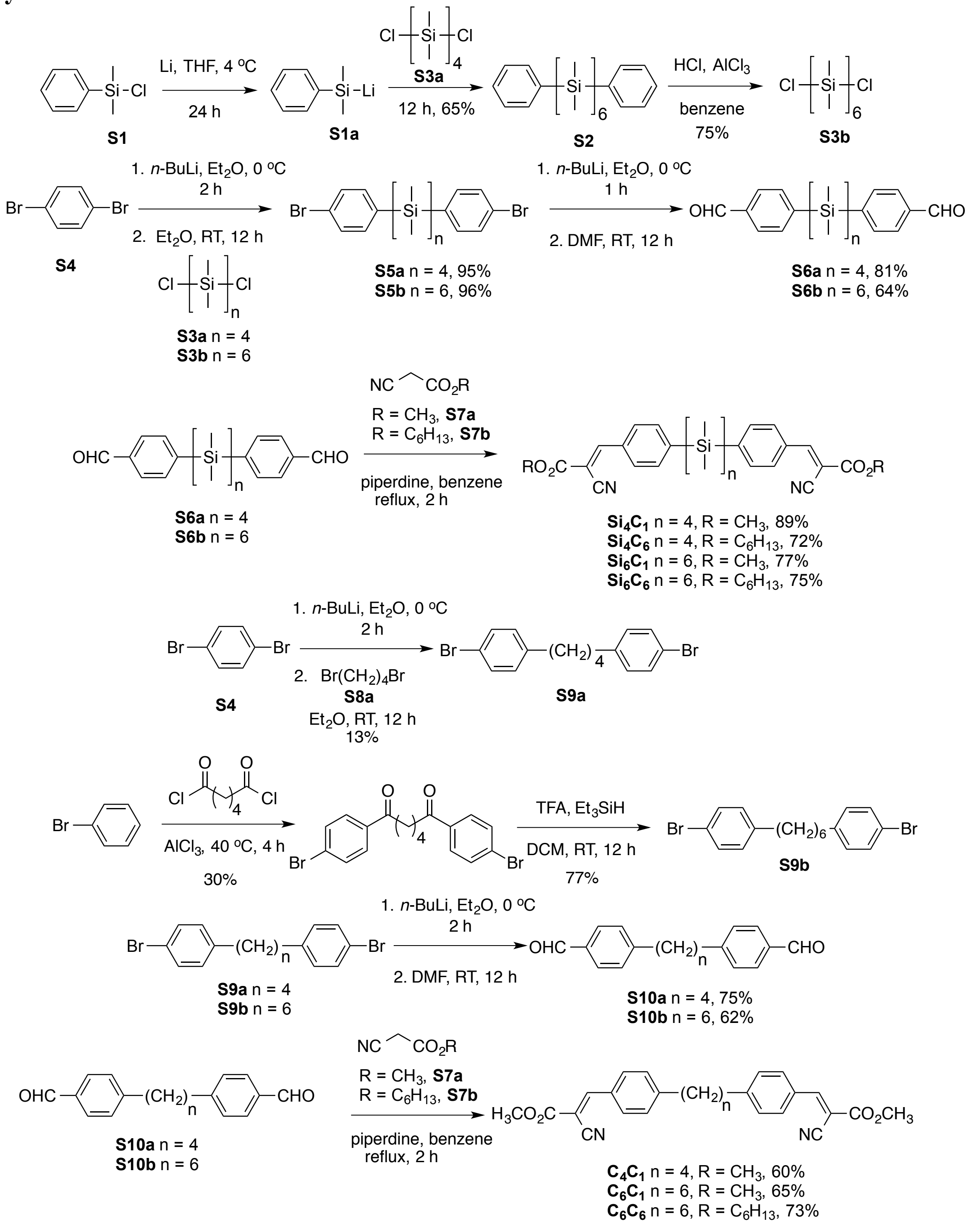

1,6-diphenyldodecamethylhexasilane (S2)

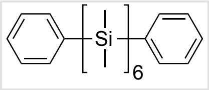

Phenyldimethylsilyllithium (**S1a**) was prepared according to adaptation of the procedure reported by Shibano, *et al.*[6] In an oven-dried 100 mL 3-neck round bottom flask fitted with an addition funnel and stir bar was cooled under argon atmosphere. To this flask was added granular lithium (0.44 g, 63.40 mmol, 8 equiv) and tetrahydrofuran (THF) (10 mL) and the suspension was cooled to 0 °C with an ice bath. Chloro(dimethyl)phenylsilane (**S1**) (2.70 mL, 15.80 mmol, 2 equiv) in THF (5 mL) was added to the lithium suspension dropwise *via* addition funnel and the resulting heterogeneous mixture was stirred for 24 hours at room temperature. During this time, the clear colorless solution turned to a dark red color. The excess lithium was then removed by filtration through a fritted schlenk funnel, while the receiving 100 mL schlenk flask was maintained at a temperature of 0 °C under argon atmosphere (Note: The filtered lithium is quenched by cautious addition of isopropanol with stirring under argon atmosphere). Isopropylmagnesium chloride (9.10 mL, 18.17 mmol, 2.3 equiv, 2 M in THF) was added dropwise to the filtrate in the receiving flask at 0 °C. Upon completion of the addition, the solution was stirred an additional 20 minutes at 0 °C. 1,4-dichlorooctamethyltetrasilane (**S3a**) (2.40 g, 7.90 mmol, 1 equiv) was then added dropwise at 0 °C. After addition, the ice bath was removed and the solution allowed to warm to room temperature and stirred overnight. The reaction mixture was then quenched by the careful addition of water (10 mL), followed by saturated ammonium chloride solution (10 mL). The aqueous and organic layers were separated using a seperatory funnel and the aqueous layer was extracted thrice with diethyl ether. The combined diethyl ether layers were dried over sodium sulfate, filtered, and concentrated on a rotary evaporator to yield a yellow colored oil. Purification by automated column chromatography on silica gel by eluting with hexanes yielded

a white solid as the title compound (2.50 g, 65% yield). ^{1}H NMR (400 MHz, $CDCl_3$) δ 7.46-7.44 (m, 4H), 7.35-7.33 (m, 6H), 0.39 (s, 12H), 0.12 (s, 12H), 0.61 (s, 12H). ^{13}C NMR (100 MHz, $CDCl_3$) δ 139.90, 133.74, 128.31, 127.70, -2.93, -4.50, -5.34. ^{29}Si NMR (80 MHz, $CDCl_3$) δ -17.96, -39.07, -43.24. HRMS (EI): calculated for $C_{24}H_{46}Si_6$ 502.2215, found 502.2212.

Dichlorododecamethylhexasilane (S3b)

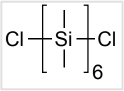

The title compound was prepared according to a similar procedure reported by Klausen *et al.*,[1] for the synthesis of dichlorooligosilanes with the following modification: 1,6-diphenyldodecamethylhexasilane (**S2**) (1.00 g, 2 mmol) was used instead. The title compound was purified by vacuum distillation (125 ^{o}C, 0.05 Torr) yielding a colorless liquid as the title compound (0.62 g, 75 % yield). ^{1}H NMR (400 MHz, C_6D_6) δ 0.43 (s, 12H), 0.26 (s, 12H), 0.23 (s, 12H).

2.1 General procedure for the synthesis of bis(4-bromophenyl)oligosilanes (**S5a-b**)

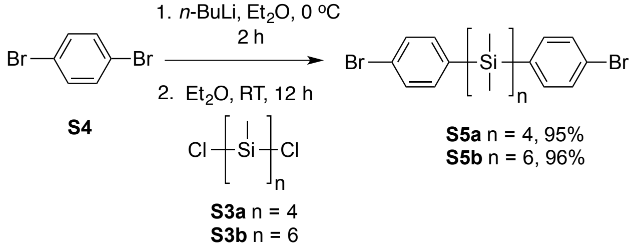

1,4-bis(4-bromophenyl)octamethyltetrasilane (S5a)

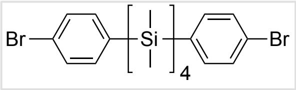

1,4-bis(4-bromophenyl)octamethyltetrasilane (**S5a**) was prepared according to adaptation of the procedure reported by Shibano, *et al.*[6] To a solution of 1,4-dibromobenzene (**S4**) (1.55 g, 6.60 mmol, 2 equiv) in diethyl ether (15 mL) in an 50 mL schlenk flask under argon atmosphere, was added *n*-butyllithium dropwise (2.64 mL, 6.60 mmol, 2 equiv, 2.5 M in hexanes) at 0 °C and the reaction mixture stirred for 2 hours at this temperature. Then, 1,4-dichlorooctamethyltetrasilane (**S3a**) (1.00 g, 3.30 mmol, 1 equiv) was added while maintaining the reaction mixture at 0 °C. After addition, the reaction mixture was gradually warmed up to room temperature and stirred overnight. The reaction mixture was then quenched by the careful addition of saturated ammonium chloride solution (10 mL). The aqueous and organic layers were separated using a seperatory funnel and the aqueous layer was extracted thrice with diethyl ether. The combined diethyl ether layers were dried over sodium sulfate, filtered, and concentrated on a rotary evaporator to yield a colorless oil. Purification by automated column chromatography on silica gel by eluting with hexanes yielded a white solid as the title compound (1.71 g, 95% yield). ^{1}H NMR (400 MHz, $CDCl_3$) δ 7.44 (d, *J* = 8 Hz, 4H), 7.24 (d, *J* = 8 Hz, 4H), 0.32 (s, 12H), 0.01 (s, 12H). ^{13}C NMR (100 MHz, $CDCl_3$) δ 138.63, 135.26, 130.87, 123.16, -3.06, -5.70. ^{29}Si NMR (80 MHz, $CDCl_3$) δ -17.41, -44.56. HRMS (EI): calculated for $C_{20}H_{32}Br_2Si_4$ 543.9927, found 543.9910.

1,6-bis(4-bromophenyl)dodecamethylhexaasilane (S5b)

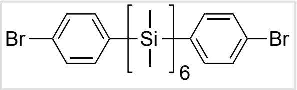

The title compound was prepared according to the general procedure described for the synthesis of **S5a** with the following modification: 1,6-dichlorododecamethylhexasilane (**S3b**) (0.61 g, 1.45 mmol, 1 equiv) was used instead of **S3a**. Purification by automated column chromatography on silica gel by eluting with hexanes yielded a white solid as the title compound (0.92 g, 96% yield). ^{1}H NMR (400 MHz, $CDCl_3$) δ 7.45 (d, *J* = 8 Hz, 4H), 7.28 (d, *J* = 8 Hz, 4H), 0.36 (s, 12H), 0.09 (s, 12H), 0.05 (s, 12H). ^{13}C NMR (100 MHz, $CDCl_3$) δ 138.80, 135.28, 130.84, 123.10, -2.96, -4.45, -5.38. ^{29}Si NMR (80 MHz, $CDCl_3$) δ -17.35, -39.08, -43.37. HRMS (EI): calculated for $C_{24}H_{44}Br_2Si_6$ 660.0405, found 660.0412.

2.2 General procedure for the synthesis of 4,4'-(oligosilanediyl)dibenzaldehyde (**S6a-b**)

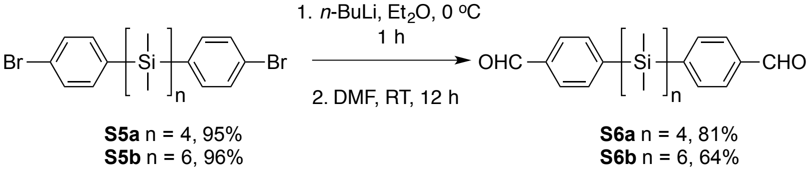

4,4'-(1,1,2,2,3,3,4,4-octamethyltetrasilane-1,4-diyl)dibenzaldehyde (S6a)

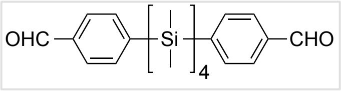

To a solution of **S5a** (1.00 g, 1.83 mmol, 1 equiv) in diethyl ether (10 mL) in a 50 mL schlenk flask under argon atmosphere was added *n*-butyllithium dropwise (1.50 mL, 3.70 mmol, 2 equiv, 2.5 M in hexanes) at 0 °C and the reaction mixture stirred for one hour at this temperature. Then, anhydrous dimethylformamide (DMF) (0.31 mL, 3.92 mmol, 2.1 equiv) was added dropwise while maintaining the reaction mixture at 0 °C (formation of a white precipitate is observed during the addition of DMF). Upon addition, the reaction mixture was gradually warmed to room temperature and stirred overnight. The reaction mixture was then quenched by the careful addition of 1N hydrochloric acid (5 mL). The aqueous and organic layers were separated using a seperatory funnel and the aqueous layer extracted thrice with diethyl ether. The combined diethyl ether layers were dried over sodium sulfate, filtered, and concentrated on a rotary evaporator to yield a white solid as the crude product. Purification by automated column chromatography on silica gel by eluting with 5% ethyl acetate/hexanes yielded a white solid as the title compound (0.64 g, 81% yield). ^{1}H NMR (400 MHz, $CDCl_3$) δ 10.00 (s, 2H), 7.80 (d, *J* = 8 Hz, 4H), 7.56 (d, *J* = 8 Hz, 4H), 0.38 (s, 12H), 0.01 (s, 12H). ^{13}C NMR (100 MHz, $CDCl_3$) δ 192.61, 136.15, 134.15, 133.75, 128.60, -3.30, -5.74. ^{29}Si NMR (80 MHz, $CDCl_3$) δ -16.94, -44.0. HRMS (EI): calculated for $C_{22}H_{34}O_2Si_4$ 442.1635, found 442.1635.

4,4'-(1,1,2,2,3,3,4,4,5,5,6,6-dodecamethylhexasilane-1,6-diyl)dibenzaldehyde (S6b)

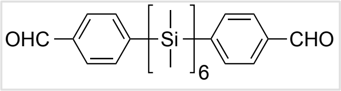

The title compound was prepared according to the above procedure described for the synthesis of **S6a** with the following modification: **S5b** (0.55 g, 0.83 mmol, 1 equiv) was used instead of **S5a**. Purification by automated column chromatography on silica gel by eluting with 5% ethyl acetate/hexanes yielded a white solid as the title compound (0.30 g, 64% yield). ^{1}H NMR (400 MHz, $CDCl_3$) δ 10.00 (s, 2H), 7.81 (d, J = 8 Hz, 4H), 7.59 (d, J = 8 Hz, 4H), 0.41 (s, 12H), 0.10 (s, 12H), 0.04 (s, 12H). ^{13}C NMR (100 MHz, $CDCl_3$) δ 192.61, 136.10, 134.18, 128.57, -3.18, -4.47, -5.40. ^{29}Si NMR (80 MHz, $CDCl_3$) δ -16.89, -38.88, -42.96. HRMS (EI): calculated for $C_{26}H_{46}O_2Si_6$ 558.2113, found 558.2107.

2.3 General procedure for the synthesis of ***Si_4C_1-Si_6C_6*** *by Knoevanagel condensation*

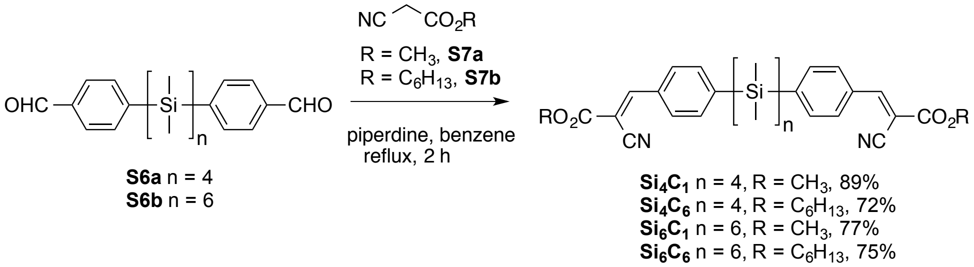

Si_4C_1

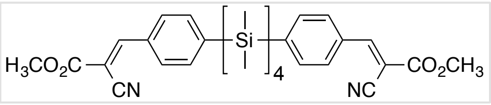

To a 50 mL schlenk flask equipped with a condenser was added **S6a** (0.25 g, 0.56 mmol, 1 equiv) and anhydrous benzene (10 mL) under argon atmosphere. To the above solution were added methyl 2-cyanoacetate (**S7a**) (0.10 mL, 1.18 mmol, 2.1 equiv) and piperidine (0.02 mL, 0.17 mmol, 0.3 equiv) (the solution immediately turns yellow upon the addition of piperidine), and the reaction mixture was heated to reflux for two hours. The reaction mixture was then cooled down, and the benzene evaporated on a rotary evaporator to yield a yellow color solid as the crude product. The crude product was recrystallized in diethyl ether at room temperature to obtain yellow crystals of the title compound (0.30 g, 89% yield). ^{1}H NMR (400 MHz, $CDCl_3$) δ 8.24 (s, 2H), 7.91 (d, J = 8 Hz, 4H), 7.54 (d, J = 8 Hz, 4H), 3.94 (s, 6H), 0.37 (s, 12H), 0.03 (s, 12H). ^{13}C NMR (100 MHz, $CDCl_3$) δ 163.10, 155.38, 148.43, 134.42, 131.13, 129.90, 115.58, 102.23, 53.38, -3.34, -5.70. ^{29}Si NMR (80 MHz, $CDCl_3$) δ -16.82, -43.74. HRMS (EI): calculated for $C_{30}H_{40}N_2O_4Si_4$ 604.2065, found 604.9632.

Si_4C_6

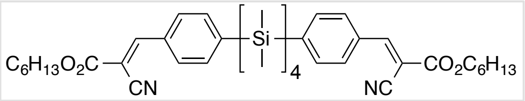

The title compound was prepared according to the above procedure described for the synthesis of **Si_4C_1** with the following modification: **S6a** (0.30 g, 0.67 mmol, 1 equiv), and hexyl 2-cyanoacetate

(**S7b**) (0.24 g, 1.40 mmol, 2.1 equiv) were used instead of **S6a** and **S7a**. Purification by automated column chromatography on silica gel by eluting with 5% ethyl acetate/hexanes yielded a yellow solid as the title compound (0.36 g, 72% yield). ^{1}H NMR (400 MHz, $CDCl_3$) δ 8.24 (s, 2H), 7.94 (d, *J* = 8 Hz, 4H), 7.56 (d, *J* = 8 Hz, 4H), 4.34 (t, *J* = 6.8, 4H), 1.8-1.76 (m, 4H), 1.45-1.34 (m, 12H), 0.93 (t, *J* = 6.8 Hz, 6H), 0.39 (s, 12H), 0.05 (s, 12H). ^{13}C NMR (100 MHz, $CDCl_3$) δ 162.70, 155.05, 148.22, 134.41, 131.24, 129.88, 115.58, 102.73, 66.81, 31.37, 28.46, 25.46, 22.51, 14.00, -3.33, -5.70. ^{29}Si NMR (80 MHz, $CDCl_3$) δ -16.88, -43.80. Elemental analysis, found: C, 64.7%; H, 8.0%; N, 3.8%. Calc. for $C_{40}H_{60}N_2O_4Si_4$: C, 64.5%; H, 8.1%; N, 3.8%. HRMS (EI): calculated for $C_{40}H_{60}N_2O_4Si_4$ 744.3630, found 744.3620.

Si_6C_1

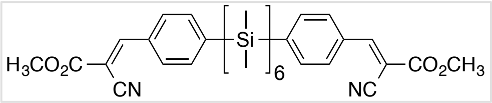

The title compound was prepared according to the above procedure described for the synthesis of **Si_4C_1** with the following modification: **S6b** (0.15 g, 0.28 mmol, 1 equiv), and methyl 2-cyanoacetate (**S7a**) (0.05 mL, 0.59 mmol, 2.1 equiv) were used instead of **S6a** and **S7a**. Purification by automated column chromatography on silica gel by eluting with 15% ethyl acetate/hexanes yielded a yellow solid as the title compound (0.14 g, 77% yield). ^{1}H NMR (400 MHz, $CDCl_3$) δ 8.24 (s, 2H), 7.92 (d, *J* = 8 Hz, 4H), 7.57 (d, *J* = 8 Hz, 4H), 3.94 (s, 6H), 0.40 (s, 12H), 0.10 (s, 12H), 0.05 (s, 12H). ^{13}C NMR (100 MHz, $CDCl_3$) δ 163.16, 155.46, 148.8, 134.47, 131.06, 129.9, 115.62, 102.11, 53.39, -3.21, -4.44, -5.40. ^{29}Si NMR (80 MHz, $CDCl_3$) δ -16.75, -38.77, -42.76. Elemental analysis, found: C, 56.4%; H, 7.2%; N, 3.7%. Calc. for $C_{34}H_{52}N_2O_4Si_6$: C, 56.6%; H, 7.3%; N, 3.9%. HRMS (EI): calculated for $C_{34}H_{52}N_2O_4Si_6$ 720.2542, found 720.2525.

Si_6C_6

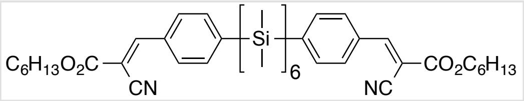

The title compound was prepared according to the above procedure described for the synthesis of **Si_4C_1** with the following modification: **S6b** (0.13 g, 0.23 mmol, 1 equiv), and hexyl 2-cyanoacetate (**S7b**) (0.08 g, 0.49 mmol, 2.1 equiv) were used instead of **S6a** and **S7a**. Purification by automated column chromatography on silica gel by eluting with 10% ethyl acetate/hexanes yielded a yellow solid as the title compound (0.15 g, 75% yield). ^1H NMR (400 MHz, $CDCl_3$) δ 8.22 (s, 2H), 7.92 (d, J = 8 Hz, 4H), 7.56 (d, J = 8 Hz, 4H), 4.31 (t, J = 5.6 Hz, 4H), 1.79-1.72 (m, 4H), 1.44-1.31 (m, 12H), 0.90 (t, J = 6.8 Hz, 6H), 0.40 (s, 12H), 0.10 (s, 12H), 0.05 (s, 12H). ^{13}C NMR (100 MHz, $CDCl_3$) δ 162.71, 155.1, 148.56, 134.44, 131.17, 129.86, 155.58, 102.64, 66.80, 31.36, 28.46, 25.45, 22.50, 14.00, -3.22, -4.44, -5.40. ^{29}Si NMR (80 MHz, $CDCl_3$) δ -16.80, -38.77, -42.78. Elemental analysis, found: C, 61.5%; H, 8.5%; N, 3.2%. Calc. for $C_{44}H_{72}N_2O_4Si_6$: C, 61.3%; H, 8.4%; N, 3.25%. HRMS (EI): calculated for $C_{44}H_{72}N_2O_4Si_6$ 860.4107, found 860.4088.

2.4 Synthesis of alkanes $\mathbf{C_4C_1}$*,* $\mathbf{C_6C_1}$ ***and*** $\mathbf{C_6C_6}$

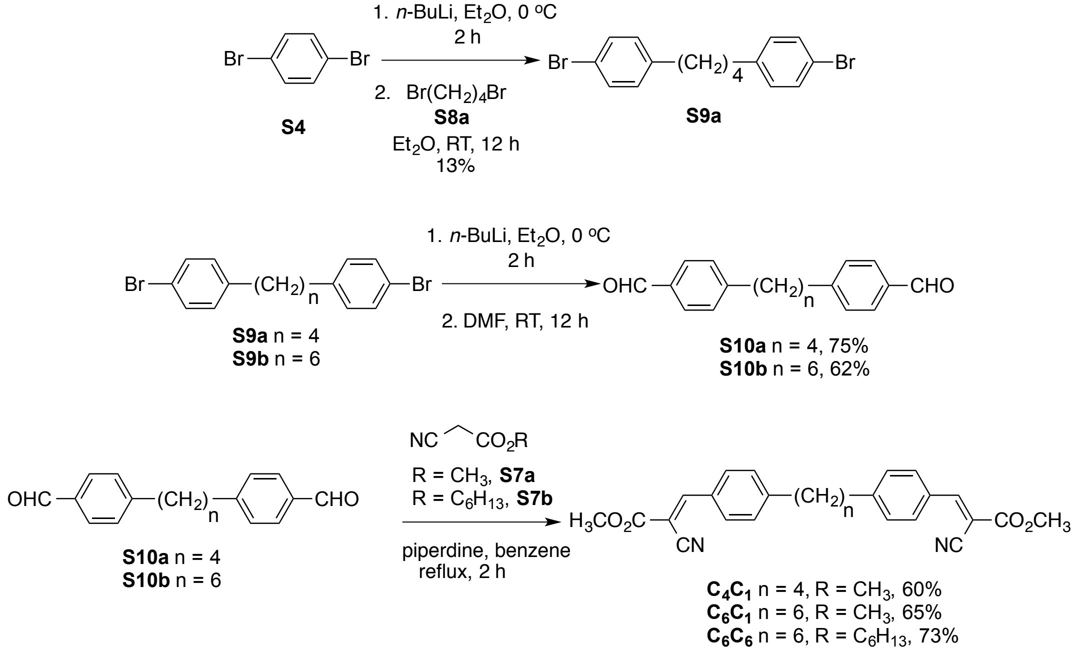

1,4-bis(4-bromophenyl)butane (S9a)

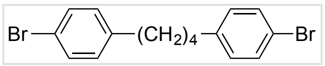

To an ether solution of 1,4-dibromobenzene (**S4**) (5.00 g, 21.20 mmol, 2 equiv) in a two neck round bottom flask equipped with an addition funnel was added *n*-butyllithium (8.50 mL, 21.20 mmol, 2 equiv, 2.5 M in hexanes) dropwise under argon atmosphere at 0 °C and the reaction mixture stirred for 2 hours at this temperature. Then, 1,4-dibromobutane (**S8a**) (1.30 mL, 10.60 mmol, 1 equiv) was added dropwise *via* the addition funnel at 0 °C. After addition, the reaction mixture was gradually warmed up to room temperature and stirred overnight. The reaction mixture was then quenched by the careful addition of saturated ammonium chloride solution (30 mL). The aqueous and organic layers were separated using a seperatory funnel and the aqueous layer extracted thrice with diethyl ether. The combined diethyl ether layers were dried over sodium sulfate, filtered, and concentrated on a rotary evaporator to yield a colorless oil as the crude product. Purification by automated column chromatography on silica gel by eluting with hexanes yielded a white solid. Further purification

by recrystallization of the white solid in methanol at room temperature yielded the title compound as a colorless crystalline solid (0.40 g, 13% yield). ^{1}H NMR (400 MHz, $CDCl_3$) δ 7.38 (d, *J* = 8 Hz, 4H), 7.02 (d, *J* = 8 Hz, 4H), 2.57 (t, *J* = 6.4, 4H), 1.63-1.60 (m, 4H). ^{13}C NMR (100 MHz, $CDCl_3$) δ 141.26, 131.32, 130.13, 119.42, 35.12, 30.70. HRMS (EI): calculated for $C_{16}H_{16}Br_2$ 367.9598, found 367.9590.

S10a

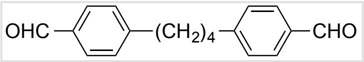

The title compound was prepared according to the procedure described for the synthesis of **S6a** with the following modification: **S9a** (0.30 g, 0.83 mmol, 1 equiv) was used instead of **S5a**. Purification by automated column chromatography on silica gel by eluting with 10% ethyl acetate/hexanes yielded a white solid as the title compound (0.15 g, 75% yield). ^{1}H NMR (400 MHz, $CDCl_3$) δ 9.97 (s, 2H), 7.79 (d, *J* = 8 Hz, 4H), 7.32 (d, *J* = 8 Hz, 4H), 2.73 (t, *J* = 6.4 Hz, 4H), 1.72-1.68 (m, 4H). ^{13}C NMR (100 MHz, $CDCl_3$) δ 191.94, 149.7, 134.58, 130, 1293.06, 36.0, 30.54. HRMS (EI): calculated for $C_{18}H_{18}O_2$ 266.1306, found 266.1298.

S10b

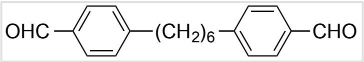

The title compound was prepared according to the procedure described for the synthesis of **S6a** with the following modification: **S9b** (0.71 g, 1.80 mmol, 1 equiv) was used instead of **S5a**. Purification by automated column chromatography on silica gel by eluting with 10% ethyl acetate/hexanes yielded a white solid as the title compound (0.32 g, 62% yield). ^{1}H NMR (400 MHz, $CDCl_3$) δ 9.97 (s, 2H), 7.79 (d, *J* = 8 Hz, 4H), 7.32 (d, *J* = 8 Hz, 4H), 2.67 (t, *J* = 7.6 Hz, 4H), 1.64 (t, J = 7.2, 4H), 1.39-1.35 (m, 4H). ^{13}C NMR (100 MHz, $CDCl_3$) δ 192, 150.2, 134.47, 129.91, 129.06, 36.14, 30.91, 29.02. HRMS (EI): calculated for $C_{20}H_{22}O_2$ 294.1619, found 294.1616.

C_4C_1

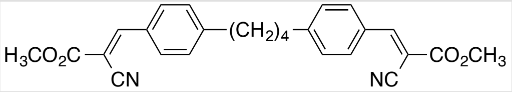

The title compound was prepared according to the procedure described for the synthesis of **Si_4C_1** with the following modification: **S10a** (0.12 g, 0.50 mmol, 1 equiv) and **S7a** (0.1 mL, 1 mmol, 2.1 equiv) were used instead of **S6a** and **S7a**. Purification by recrystallization in ethyl acetate at room temperature yielded a cream color solid as the title compound (0.10 g, 60% yield). ^{1}H NMR (400 MHz, $CDCl_3$) δ 8.23 (s, 2H), 7.92 (d, J = 8 Hz, 4H), 7.29 (d, J = 8 Hz, 4H), 3.93 (s, 6H), 2.72 (br t, 4H), 1.7 (br t, 4H). ^{13}C NMR (100 MHz, $CDCl_3$) δ 163.26, 155.19, 148.9, 131.43, 129.4, 129.25, 115.76, 101.38, 53.34, 35.92, 30.46. HRMS (EI): calculated for $C_{26}H_{24}N_2O_4$ 428.1736, found 428.1724.

C_6C_1

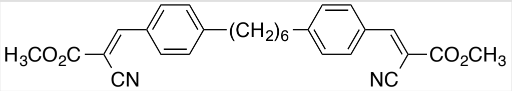

The title compound was prepared according to the procedure described for the synthesis of **Si_4C_1** with the following modification: **S10b** (0.22 g, 0.76 mmol, 1 equiv) and **S7a** (0.15 mL, 1.6 mmol, 2.1 equiv) were used instead of **S6a** and **S7a**. Purification by recrystallization in ethyl acetate at room temperature yielded a light yellow color solid as the title compound (0.22 g, 65% yield). ^{1}H NMR (400 MHz, $CDCl_3$) δ 8.23 (s, 2H), 7.92 (d, J = 8 Hz, 4H), 7.29 (d, J = 8 Hz, 4H), 3.93 (s, 6H), 2.67 (t, J = 7.6 Hz, 4H), 1.65-1.6 (m, 4H), 1.37 (br t, 4H). ^{13}C NMR (100 MHz, $CDCl_3$) δ 163.3, 155.28, 149.47, 131.39, 129.4, 129.10, 115.77, 101.21, 53.32, 36.1, 30.83, 29.0. HRMS (EI): calculated for $C_{28}H_{28}N_2O_4$ 456.2049, found 456.2049.

C_6C_6

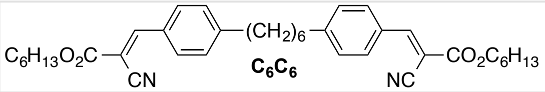

The title compound was prepared according to the procedure described for the synthesis of **Si_4C_1** with

the following modification: **S10b** (0.14 g, 0.50 mmol, 1 equiv) and **S7b** (0.18 g, 1.05 mmol, 2.1 equiv) were used instead of **S6a** and **S7a**. Purification by automated column chromatography on silica gel by eluting with 10% ethyl acetate/hexanes yielded a crystalline white solid as the title compound (0.22 g, 73% yield). ^{1}H NMR (400 MHz, $CDCl_3$) δ 8.24 (s, 2H), 7.94 (d, *J* = 8 Hz, 4H), 7.32 (d, *J* = 8 Hz, 4H), 4.3 (t, *J* = 8 Hz, 4H), 2.69 (t, *J* = 7.6 Hz, 4H), 1.8-1.76 (m, 4H), 1.66-1.64 (m, 4H), 1.46-1.35 (m, 16H), 0.93 (t, *J* = 8 Hz, 6H). ^{13}C NMR (100 MHz, $CDCl_3$) δ 162.87, 154.94, 149.29, 131.32, 129.37, 129.19, 115.75, 101.72, 66.74, 36.09, 31.37, 30.83, 29.02, 28.48, 25.46, 22.50, 14. HRMS (EI): calculated for $C_{38}H_{48}N_2O_4$ 596.3614, found 596.3627.

3. UV-vis Absorption Spectroscopy

Absorption spectra were collected on a Varian Cary50 Bio UV-Visible spectrophotometer at room temperature. Stock solutions of all compounds were prepared in chloroform at 1.0 x 10^{-3} M concentration. A 1-cm cuvette was used to record the absorption spectra. To the cuvette was added 50 µL of stock solution of the compound and 3 mL of chloroform (new concentration: 1.64 x 10^{-5} M), and the absorption spectrum recorded. Absorption spectra of compounds $\mathbf{C_4C_1}$, $\mathbf{C_6C_1}$ and $\mathbf{C_6C_6}$ were recorded at a different concentration: 1.32 x 10^{-5} M (obtained by adding 40 µL of 1 mM stock solution to 3 mL of chloroform).

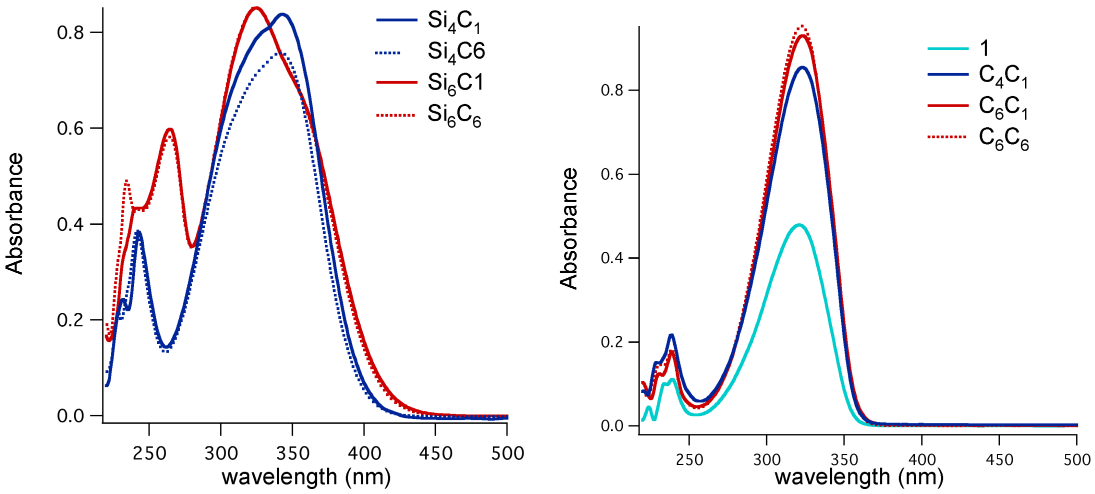

Figure S1. UV-Vis absorbance spectra of cyanovinylsilanes and cyanovinylalkanes.

4. Cyclic Voltammetry

Cyclic voltammetry measurements were performed under air-free and moisture-free conditions in a one-chamber, three-electrode cell using a PGSTAT302 potentiostat. A platinum (Pt) button electrode (2 mm^2) was used as the working electrode, a Pt wire as the counter electrode and an Ag/AgCl as the reference electrode. All cyclic voltammograms were taken in 0.1 M n-Bu_4NPF_6/ acetonitrile solutions of the analyte at a scan rate of 0.1 V/s with ferrocene (Fc) as the internal standard.

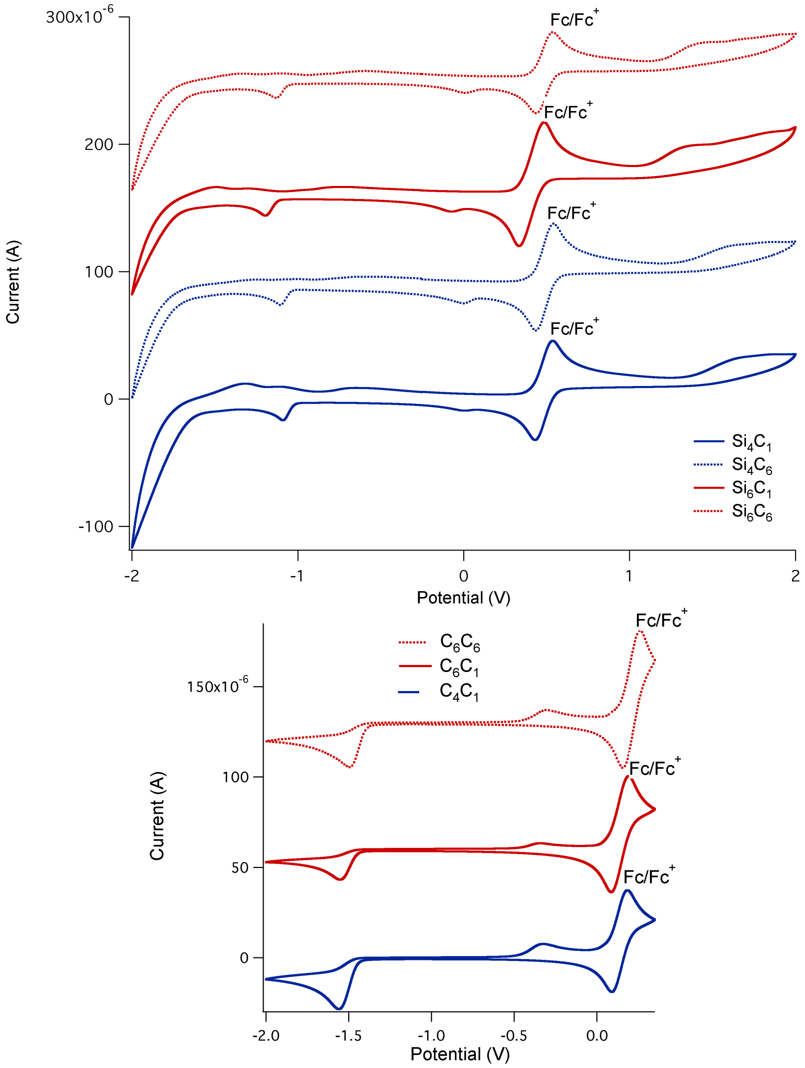

Figure S2. Cyclic voltammetry of cyanovinylsilanes and cyanovinylalkanes.

Table S1. Oxidation and reduction potentials relative to Fc/Fc^+ internal standard. Fc = ferrocene.

Compound	E_{red} (V)	E_{red} (V)	E_{ox} (V)
Si_4C_1	0.425	1.52	1.10
Si_4C_6	0.434	1.53	1.06
Si_6C_1	0.401	1.53	0.85
Si_6C_6	0.430	1.57	0.86
C_4C_1	1.65	-	-
C_6C_1	1.64	-	-
C_6C_6	1.66	-	-

5. Single Crystal X-Ray Diffraction

A. Single Crystal Growth Conditions

Si_4C_1: Single crystals suitable for X-ray diffraction were obtained from a mixture of benzene (0.4 mL) and dimethylsulfoxide (DMSO) (0.1 mL) by slow evaporation at room temperature. In a vial the compound was dissolved in benzene. To the above solution DMSO was added and the vial was capped (the vial cap was not tightened completely) and placed on a shelf at room temperature. The crystals thus obtained were yellow colored and needle shaped.

Si_6C_1: Single crystals suitable for X-ray diffraction were obtained by the liquid-liquid diffusion method from a solution of the compound in dichloromethane (≈0.5 mL) layered with hexane (≈0.3 mL) by slow evaporation at room temperature. In a vial the compound was dissolved in dichloromethane (DCM). To the above solution hexane was carefully added on the walls of the vial resulting in formation of a thin layer over the DCM solution. The vial was capped (the vial cap was not tightened completely) and placed on a shelf at room temperature. The crystals thus obtained were yellow colored.

C_6C_6: Single crystals suitable for X-ray diffraction were obtained from a mixture of hexanes (90%) and ethyl acetate (10%) at room temperature. Upon purification of the compound using automated flash column chromatography, colorless cubic crystals were observed in test tube fractions containing the compound, which were used for X-ray diffraction.

1: Single crystals suitable for X-ray diffraction were obtained from a solution of compound in benzene-d_6 by slow evaporation at room temperature. NMR sample of the compound prepared in bezene-d_6 in a NMR tube was left at room temperature for slow evaporation. The crystals thus obtained were colorless and needle shaped.

B. Single Crystal X-Ray Crystallography

All reflection intensities were measured at 110(2) K using either a KM4/Xcalibur diffractometer (detector: Sapphire3) with enhance graphite-monochromated Mo *K*α radiation (λ = 0.71073 Å) for compound **1** or a SuperNova diffractometer (equipped with Atlas detector) with Cu *K*α radiation (λ = 1.54178 Å) for compounds $\mathbf{C_6C_6}$, $\mathbf{Si_4C_1}$ and $\mathbf{Si_6C_1}$ under the program CrysAlisPro (Versions1.171.36.24 or 1.171.36.32 Agilent Technologies, 2013). The same program was used to refine the cell dimensions and for data reduction. The structure was solved with the program SHELXS-2013 (Sheldrick, 2013) and was refined on F^2 with SHELXL-2013 (Sheldrick, 2013). Analytical numeric absorption corrections based on a multifaceted crystal model were applied using CrysAlisPro. The temperature of the data collection was controlled using the system Cryojet (manufactured by Oxford Instruments). The H atoms were placed at calculated positions using the instructions AFIX 23, AFIX 43 or AFIX 137 with isotropic displacement parameters having values 1.2 or 1.5 times *U*eq of the attached C atoms.[9]

The structures of **1**, $\mathbf{C_6C_6}$, $\mathbf{Si_4C_1}$ and $\mathbf{Si_6C_1}$ are ordered.

1, Fw = 201.22, colorless block, 0.36 × 0.19 × 0.12 mm^3, triclinic, *P*-1 (no. 2), a = 4.00444(19), b = 11.1381(6), c = 12.2020(7) Å, α = 71.955(5), β = 89.514(4), γ = 80.992(4)°, V = 510.62(5) Å^3, Z = 2, D_x = 1.309 g cm^{-3}, μ = 0.090 mm^{-1}, T_{min}–T_{max}: 0.977–0.991. 7897 Reflections were measured up to a resolution of $(\sin \theta/\lambda)_{max}$ = 0.65 Å^{-1}. 2342 Reflections were unique (R_{int} = 0.0374), of which 1907 were observed [$I > 2\sigma(I)$]. 138 Parameters were refined. $R1/wR2$ [$I > 2\sigma(I)$]: 0.0412/0.1083. $R1/wR2$ [all refl.]: 0.0507/0.1126. S = 1.083. Residual electron density found between −0.20 and 0.21 e Å^{-3}.

C_6C_6: Fw = 596.78, irregular colorless block, 0.35 × 0.25 × 0.17 mm^3, monoclinic, $P2_1/c$ (no. 14), a = 14.93217(16), b = 14.22602(15), c = 15.93712(17) Å, β = 100.8897(10)°, V = 3324.48(6) $Å^3$, Z = 4, D_x = 1.192 g cm^{-3}, μ = 0.603 mm^{-1}, T_{min}-T_{max}: 0.872–0.928. 22101 Reflections were measured up to a resolution of $(\sin \theta/\lambda)_{max}$ = 0.62 $Å^{-1}$. 6527 Reflections were unique (R_{int} = 0.0223), of which 5792 were observed [$I > 2\sigma(I)$]. 399 Parameters were refined. $R1/wR2$ [$I > 2\sigma(I)$]: 0.0335/0.0893. $R1/wR2$ [all refl.]: 0.0382/0.0935. S = 1.041. Residual electron density found between −0.18 and 0.25 e $Å^{-3}$.

Si_4C_1: Fw = 605.00, pale yellow needle, 0.41 × 0.05 × 0.04 mm^3, triclinic, P-1 (no. 2), a = 8.5558(5), b = 11.8870(4), c = 17.0071(8) Å, α = 99.115(3), β = 92.129(4), γ = 94.397(4)°, V = 1700.67(14) $Å^3$, Z = 2, D_x = 1.181 g cm^{-3}, μ = 1.901 mm^{-1}, T_{min}-T_{max}: 0.621–0.935. 18214 Reflections were measured up to a resolution of $(\sin \theta/\lambda)_{max}$ = 0.62 $Å^{-1}$. 6566 Reflections were unique (R_{int} = 0.0506), of which 4730 were observed [$I > 2\sigma(I)$]. 371 Parameters were refined. $R1/wR2$ [$I > 2\sigma(I)$]: 0.0629/0.1706. $R1/wR2$ [all refl.]: 0.0889/0.1941. S = 1.026. Residual electron density found between −0.43 and 0.97 e $Å^{-3}$.

Si_6C_1: Fw = 721.31, pale yellow lath, 0.34 × 0.06 × 0.04 mm^3, triclinic, P-1 (no. 2), a = 8.6514(2), b = 11.8373(4), c = 20.4256(6) Å, α = 82.751(3), β = 87.339(2), γ = 86.175(2)°, V = 2068.90(11) $Å^3$, Z = 2, D_x = 1.158 g cm^{-3}, μ = 2.172 mm^{-1}, abs. corr. range: 0.668–0.925. 28024 Reflections were measured up to a resolution of $(\sin \theta/\lambda)_{max}$ = 0.62 $Å^{-1}$. 8119 Reflections were unique (R_{int} = 0.0906), of which 6072 were observed [$I > 2\sigma(I)$]. 430 Parameters were refined using 93 restraints. $R1/wR2$ [$I > 2\sigma(I)$]: 0.0643/0.1681. $R1/wR2$ [all refl.]: 0.0838/0.1887. S = 1.041. Residual electron density found between −0.37 and 1.15 e $Å^{-3}$.

C. Relevant Crystal Structures from Cambridge Crystallographic Database

References to crystal structures of all-*anti*-silanes and eclipsed silanes from the Cambridge Crystallographic Database:

1. Sanji, T.; Yoshiwara, A.; Kibe, T.; Sakurai, H. *Silicon Chem*. **2003**, *2*, 151.
2. Oshita, J.; Iida, T.; Ohta, N.; Komaguchi, K.; Shiotani, M.; Kunai, A. *Org. Lett*. **2002**, *4*, 403.
3. Mignani, G.; Barzoukas, M.; Zyss, J.; Soula, G.; Balegroune, F.; Grandjean, D.; Josse, D. *Organometallics*, **1991**, *10*, 3660.
4. Gualco, P.; Amgoune, A.; Miqueu, K.; Ladeira, S.; Bourissou, D. *J. Am. Chem. Soc.* **2011**, *133*, 4257.
5. Nakanishi, W.; Hitosugi, S.; Shimada, Y.; Isobe, H. *Chem. Asian J.* **2011**, *6*, 554.

D. Description of Short Contacts

Some short intermolecular contacts in the C_6C_6 crystal structure involving the central alkane linkers are highlighted below.

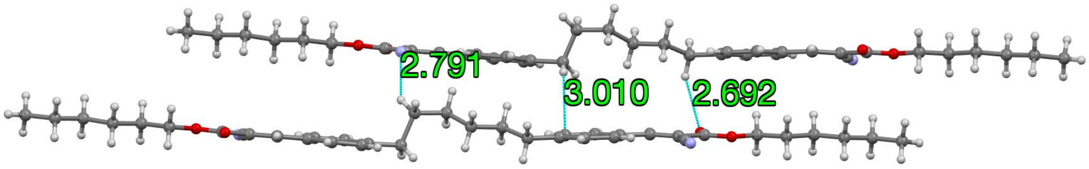

Figure S3. Crystal packing of C_6C_1. Three CH-pi short contacts are identified involving carbons in the central linker.

Some short intermolecular contacts in the Si_6C_1 unit cell are highlighted below. Green: CH−π interaction (CH−Nitrile), 3.06 Å; Purple: bifurcated hydrogen bond (CH−O), 2.63 and 2.79 Å; Blue: carbonyl-carbonyl distance, 3.44 Å.

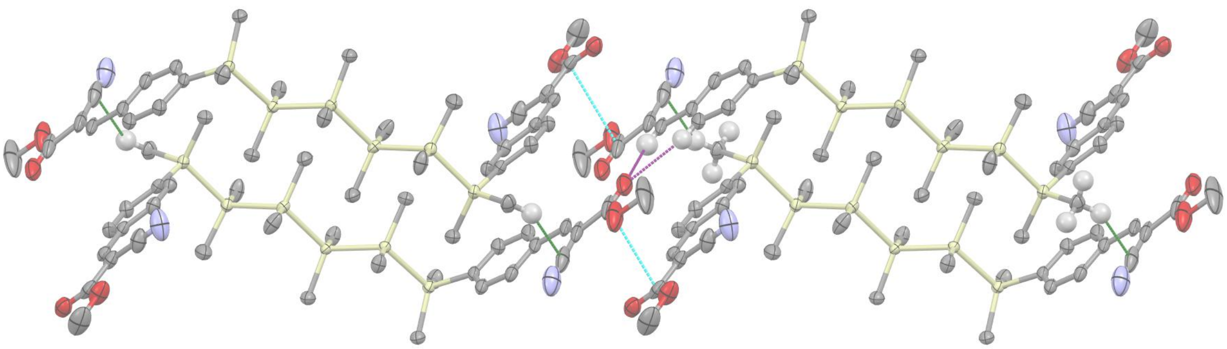

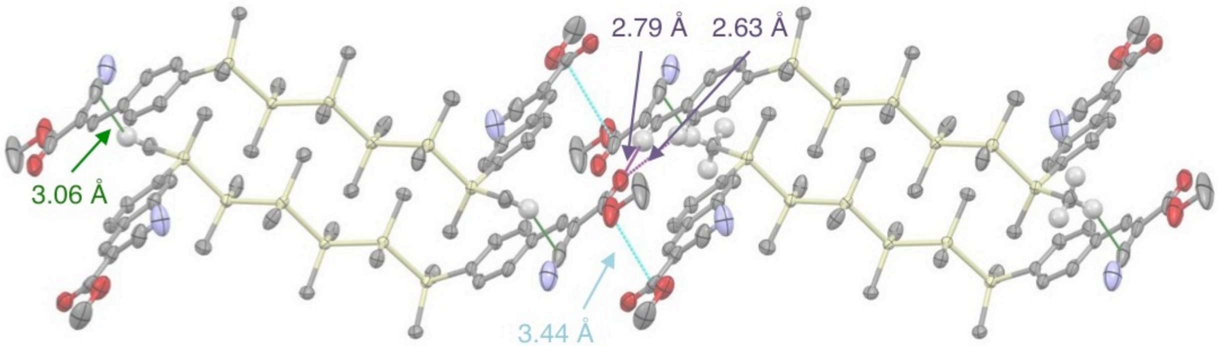

Figure S4. Crystal packing of Si_6C_1 extended along the **c** direction. Short contacts are highlighted as described above. Yellow = silicon; gray = carbon; red = oxygen; blue = nitrogen. Some hydrogens are omitted for clarity. Displacement ellipsoids shown at 50% probability.

6. Thin Film X-Ray Diffraction

XRD samples were drop-cast on Si/SiO_2 substrates with 10 mg/mL solution of different oligosilanes, made from anhydrous 1,2-dichlorobenzene and filtered thought 0.45 μm PTFE filter. The cast sample covered with Petri dish was placed inside a fume hood and dried for two days at room temperature. XRD spectra were acquired in the Bragg-Brentano (θ-2θ) geometry using a Phillips X-pert Pro X-ray diffraction system with the X-ray source wavelength of 1.54Å (Cu Kα). Scan step size was 0.02° and time per step was 2 seconds. The XRD patterns were summarized in Table S2.

Table S2. Thin film XRD patterns of the oligosilanes and alkanes. Values in the parentheses are the *d*-spacings (Å) corresponding to the diffraction angles 2θ (degrees) as determined by Bragg's Law (λ = 2d sinθ).

Compound	Diffraction Angles 2θ and *d*-Spacings
Si_4C_1	7.8(11.3), 15.3(5.8)
Si_4C_6	4.9(18.1), 5.8(15.1), 7.3(12.0), 9.5(9.3), 13.2(6.7), 16.2(5.5), 18.6(4.8), 23.2(3.8)
Si_6C_1	4.7(18.9), 7.7(11.5)
Si_6C_6	6.4 (13.8), 6.7 (13.3), 9.5 (9.3), 9.87(9.0), 12.7 (7.0)
C_4C_1	9.2(9.6)
C_6C_1	8.1(11.0)
C_6C_6	6.8(12.9), 8.0(11.1), 9.8(9.0), 14.6(6.1), 15.7(5.7), 21.4(4.1), 22.2(4.0), 23.4(3.8)

Si_6C_1 Assignments. The most intense peak (4.7° (18.9 Å)) is consistent with the unit cell c-axis (20.4 Å) and the second, less intense peak (7.7° (11.5 Å)) is consistent with the unit cell b-axis (11.8 Å) (unit cell dimensions, a = 8.65 Å, b = 11.8 Å, c = 20.4 Å).

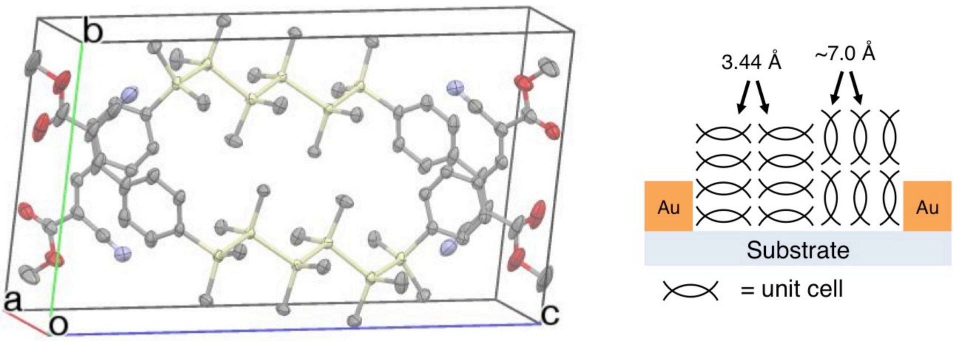

Figure S5. Schematic representation of polycrystalline thin film structure and relevance to device properties.

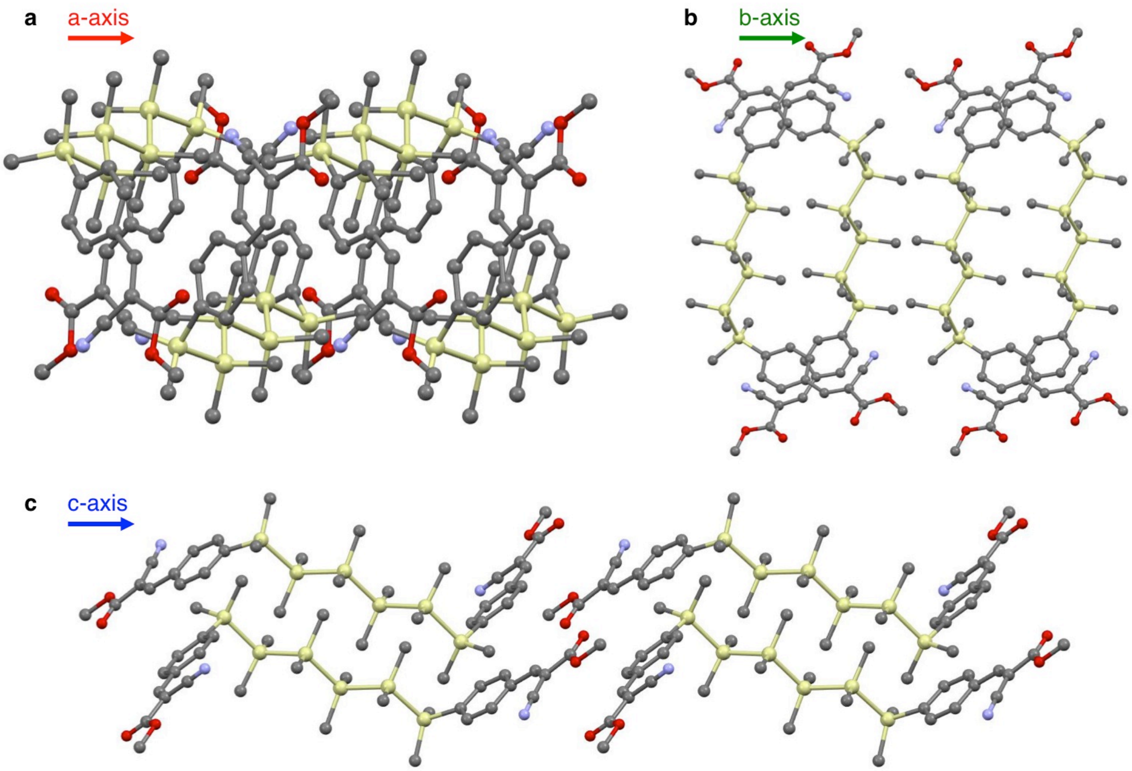

Figure S6. Crystal packing of Si_6C_1 extended along the a) **a** axis direction, b) **b** axis direction, and c) **c** axis direction. In all cases, the unit cell axis is oriented horizontally.

These d-spacings suggest that the Si_6C_1 dimers which make up the unit cell are oriented both parallel (reflection from the (010) plane, b = 11.8 Å) and perpendicular (reflection from the

(001) plane, c = 20.4 Å) to the substrate. This is illustrated in the above schematic. In our device fabrication scheme, the intermolecular spacing in the parallel orientation is shorter (3.44 Å between carbonyl carbons, see Figure 2c) than in the perpendicular orientation (~7 Å between silane chains).

Si_6C_6 Assignments. Chem 3D modeling of an all-*anti*-hexasilane with methyl side chains predicts a molecular length of nearly 30 Å and of an all-*anti*-hexasilane with hexyl side chains predicts a molecular length >41 Å.

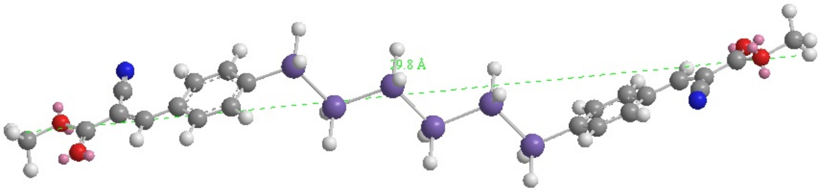

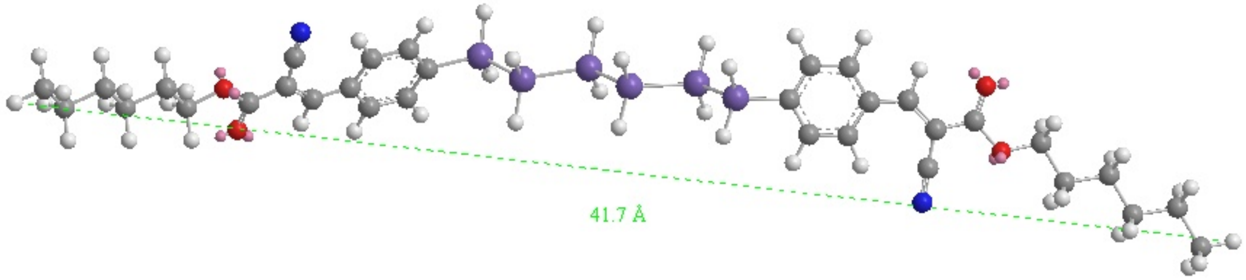

However, the longest d-spacing observed in the thin film XRD pattern is 13.8 Å. We suggest that the 13.8 Å d-spacing may be consistent with reflection from the (010) plane as replacement of the methyl groups of Si_6C_1 with *n*-hexyl groups would lengthen the unit cell b-axis. The higher order reflection at $2\theta = 12.7°$ (d = 7.0 Å) is assigned to the (020) plane.

7. Optical Microscopy and Atomic Force Microscopy

Optical microscopic images of the oligosilane films were taken using Vista Vision 82026-636 Metallurgical Microscope. The oligosilane films were the same samples used for thin film XRD measurements. Si_6C_1 formed highly crystalline isolated needles, whereas Si_6C_6 resulted in densely-packed layer of parallel-oriented structures. These results are in line with the observations from the difference in XRD peak intensity of two films.

Surface profiles of organosilane films were acquired using a Nanoscope V (Digital Instruments) atomic force microscope in tapping mode with a Cr/Pt-coated tip (f = 150 kHz, 40 N/m, BudgetSensors). Images for Si_6C_1 samples were acquired in regions between macroscopic needle features (Δz > 400 nm). Images of sample Si_6C_6 were acquired approximately 50 μm from the film edge with the underlying Si/SiO_2 substrate. Si_6C_6 display a lamellar sheet morphology. As seen below, a section of the Si_6C_6 sample shows individual sheets have a thickness of approximately 2.5-3 nm in height. Multilayers can also be observed.

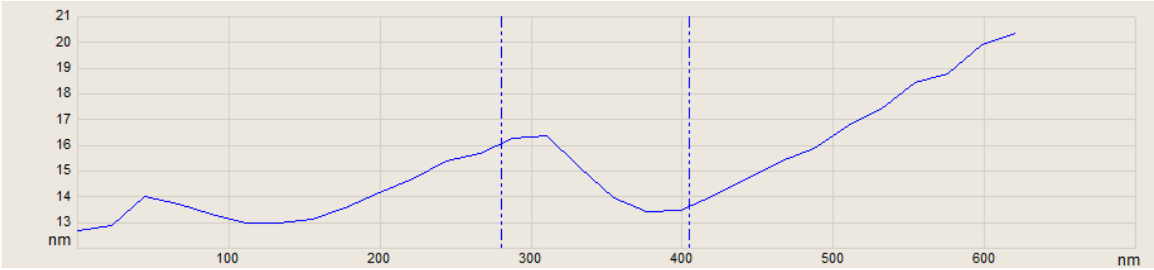

Figure S7. A typical AFM height profile of Si_6C_6 sample showing approximately 2.5 nm sheet height.

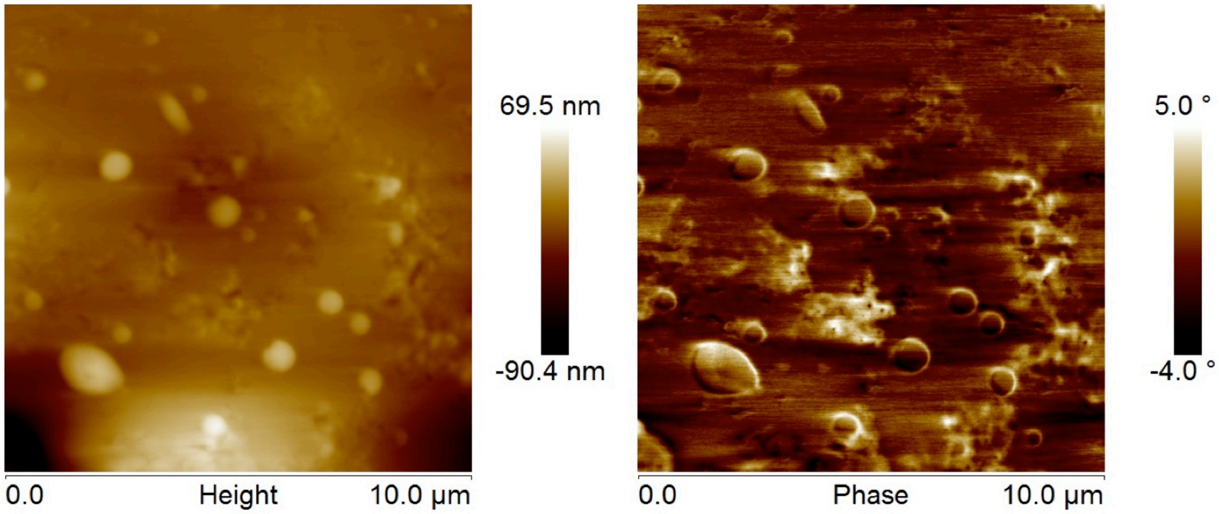

Figure S8. AFM height and phase images of Si_6C_1 taken between macroscopic needle features. Islands of material are visible. Macroscopic needle features are too tall (up to 700 nm) to image well by AFM.

8. Differential Scanning Calorimetry (DSC) Thermograms

DSC thermograms of the oligosilanes and alkanes were shown in Figure S9, with the detailed numbers summarized in Table S3. The measurements were carried out with a TA Instruments DSC Q20 at heating rate of 5 °C/min and cooling rate of 1 °C/min, with a nitrogen flow of 50 mL/min. The reported data were obtained from the second heating/cooling cycle.

Table S3. Transition temperatures of the oligosilanes and alkanes in heating and cooling steps. Values in the parentheses are the transition enthalpies (J/g).

	Temperature (^oC)	
**Compound**	**Heating**	**Cooling**
Si_4C_1	133.6 (61.7)	58.8 (36.1)
Si_4C_6	76.6 (1.5), 87.2 (81.7)	38.4 (69.5)
Si_6C_1	156.7 (70.0)	94.4 (64.4)
Si_6C_6	86.5 (51.5), 92.6 (10.3)	62.8 (50.0)
C_4C_1	192.3 (127.7)	181.0 (60.3), 177.7 (58.2)
C_6C_1	133.2 (100.6)	109.9 (98.0)
C_6C_6	82.7 (106.2), 91.6 (19.3)	73.5 (117.1)

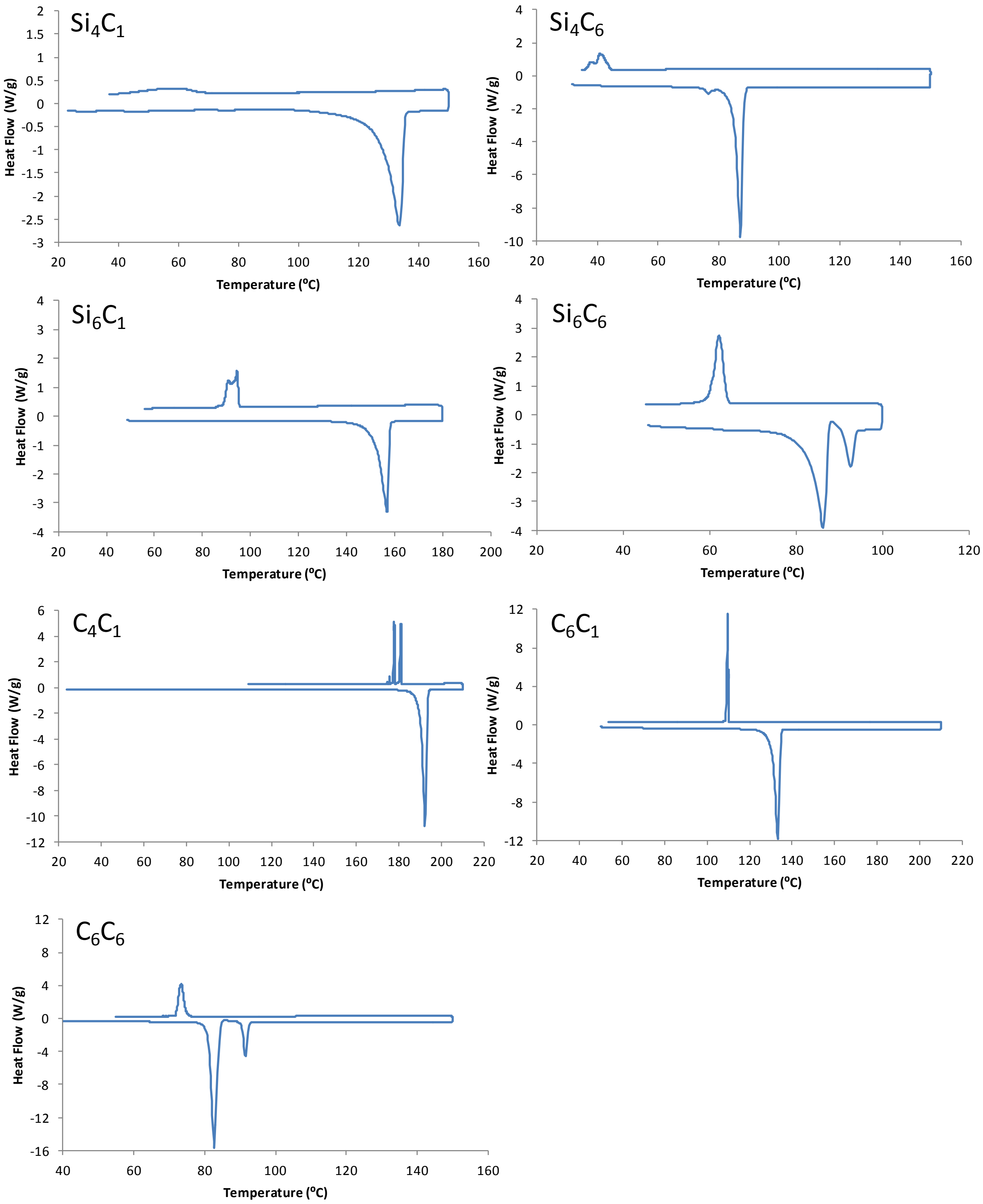

Figure S9. DSC thermograms of the oligosilanes and alkanes (exothermic up).

9. Space-Charge Limited Current (SCLC) Devices

Device Fabrication: The device scheme is shown in Figure S10. Glass slides were cut then cleaned by sequential ultrasonication in acetone and 2-propanol (20 minutes each) and subsequently dried with a nitrogen flow. 50-nm gold electrodes were thermally evaporated onto the clean glass slides at the rate of 5Å/sec (monitoring by quartz crystal microbalance) at < 5 x 10^{-6} Torr pressure in the vacuum chamber of Edwards Coating System E306A, using 25μm diameter tungsten wire as shadow mask. A rectangular area (1-mm x 2.5-mm) over the 25 μm electrode gap was defined with Novec (painted with a micropipette) and dried on 110 °C hotplate for 10 minutes in ambient conditions. 0.5 μL of a solution of oligosilane (10 mg/mL solution, anhydrous 1,2-dichlorobenzene and filtered thought 0.45 μm PTFE filter (Agela Technologies)) was drop-cast into the area defined by Novec. The cast sample was covered with a Petri dish and placed inside a fume hood and dried for one day at room temperature, and further kept in a 70°C vacuum oven (VWR International LLC) under 40 cm-Hg vacuum for one day.

Device Characterization: Current-voltage characteristics were measured under ambient conditions using an Agilent 4155C semiconductor parameter analyzer with EasyEXPERT software. The space-charge-limited mobility μ was extracted from the $J^{1/2}$-V plots in Figure S11 according to the Mott–Gurney Law:[7]

$$J = \frac{9\epsilon_0\epsilon_r\mu V^2}{8L^3}$$

where ϵ_0 is the vacuum permittivity (8.854 x 10^{-14} F cm^{-1}), ϵ_r is the relative permittivity (2.5),[8] V is the voltage applied, L is the channel length, and J is the current density, given by the current I through cross-sectional area A:

$$J = I/A$$

Cross-sectional area A was calculated as width W times height H of the thin film in the area defined by Novec:

$$A = W \times H$$

Height of the thin film (i.e. film thickness) was calculated from thin film volume V_{film}:

$$V_{film} = W \times L \times H$$

V_{film} can be calculated from the mass of the thin film M:

$$M = V_{film} \times D_x = V_{solution} \times C_{solution}$$

where D_x is mass density of the thin film (densities determined from single crystals, see table below and attached CIF files), $V_{solution}$ is the volume of the drop-cast solution (0.5 μL), and $C_{solution}$ is the concentration of the drop-cast solution (10 mg/mL).

Material	*Density (g cm^{-3})*
Si_4C_1, Si_4C_6	1.181
Si_6C_1, Si_6C_6	1.158
C_6C_6	1.192

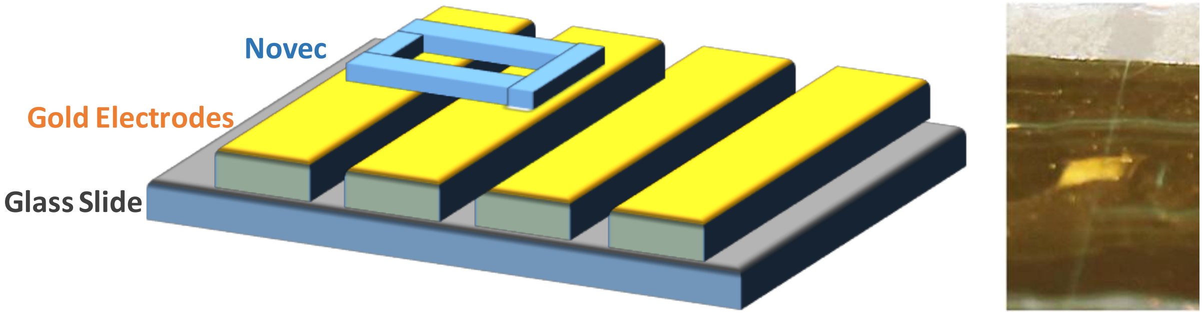

Figure S10. The left panel shows the fabrication of the oligosilane device on glass substrates, with gold as the bottom contacts and a rectangular area defined by Novec for the subsequent drop-casting. Not to scale. An image of the actual device fabricated is shown on the right panel.

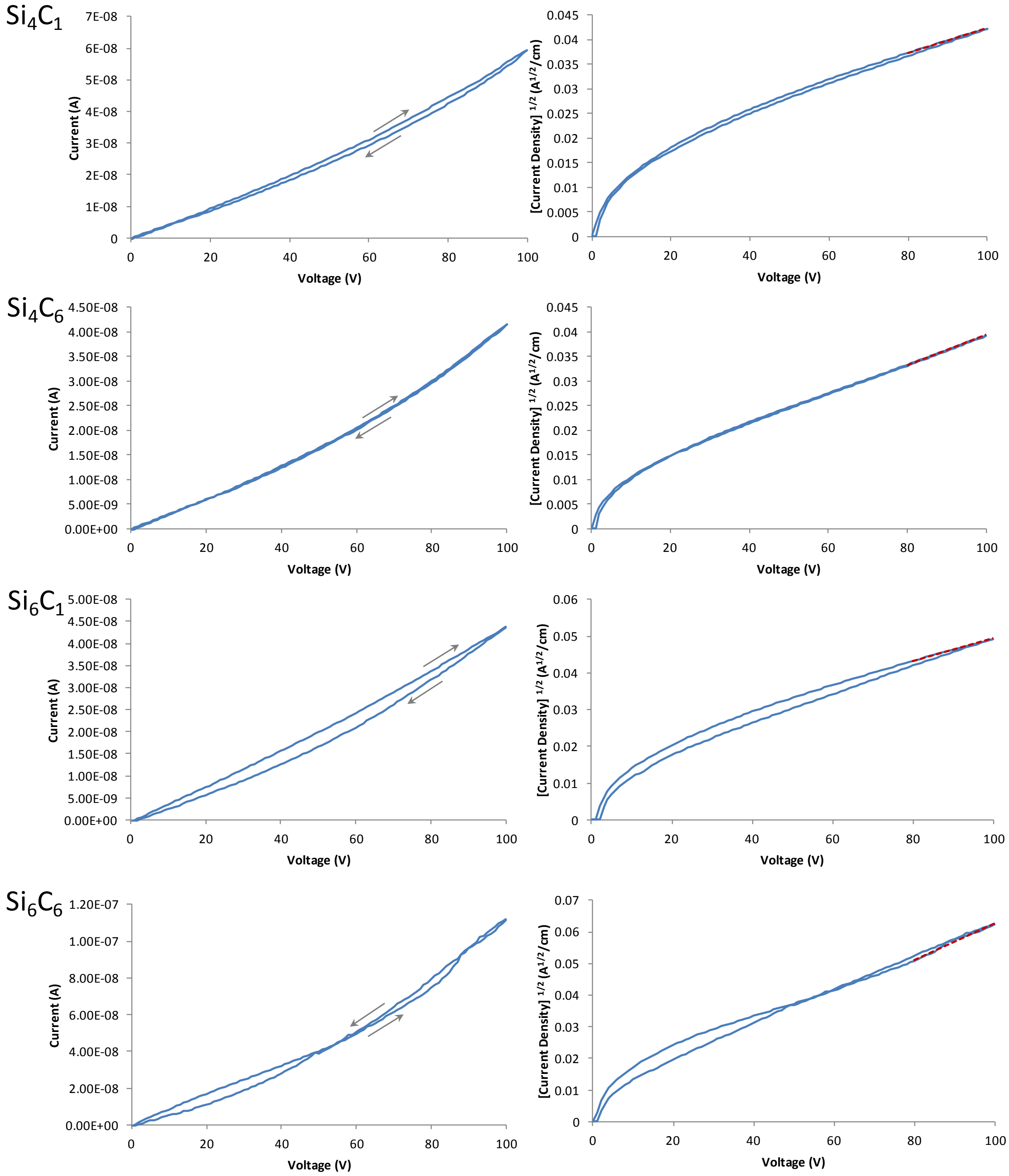

Figure S11. SCLC measurements of the oligosilanes. The left panels are the *I*-*V* plots and the right panels are the $J^{1/2}$-V plots.

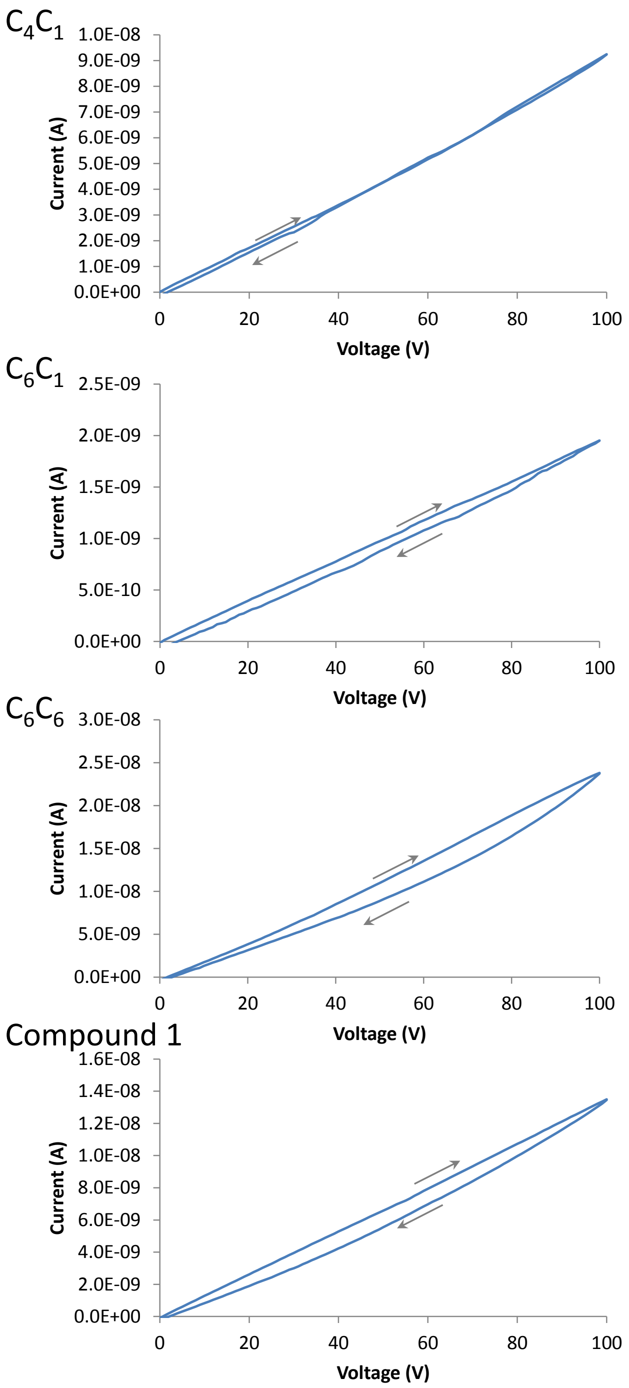

Figure S12. *I*-*V* plots from SCLC measurements of the alkanes.

Table S4. Average SCLC mobility of cyanovinylsilanes. Error bars are standard deviations.

Material	μ (x 10^{-2} cm^2/Vs)	Number of Device Tested
Si_4C_1	0.42 ± 0.06	5
Si_4C_6	0.54 ± 0.07	6
Si_6C_1	0.57 ± 0.06	5
Si_6C_6	2.29 ± 0.90	5

10. Thin Film Transistor Devices

Device Fabrication: The fabrication of TFT devices is similar to that of SCLC devices. Instead of glass slides, substrates with Si/SiO_2 stack (heavily-doped silicon wafers with 3000Å thermal oxide on top) were used as the gate electrode and dielectric layer. Si/SiO_2 substrates were cut then cleaned in piranha solution (mixture of H_2SO_4 : 30% H_2O_2 = 3:1. Danger! Highly corrosive and oxidizing) for 30 minutes. After rinsing with DI water, the substrates were cleaned by sequential ultrasonication in acetone and 2-propanol (20 minutes each) and subsequently dried with a nitrogen flow. 75μm diameter tungsten wires were used as shadow mask when gold electrodes were thermally evaporated onto the clean Si/SiO_2 substrates. Following the same procedure for SCLC devices, oligosilane solution was drop-cast into the area defined by Novec and sequentially dried under ambient condition and then in the heated vacuum oven.

Device Characterization: Current-voltage characteristics were measured under ambient conditions using an Agilent 4155C semiconductor parameter analyzer with EasyEXPERT software. The transistor mobility μ was extracted from the current I_D in the saturation regime using the $(I_D)^{1/2}$-V_G plot and the following equation:

$$I_D = \frac{W}{2L}\mu\, C_i(V_G - V_{TH})^2$$

where W and L are the device channel width and length, C_i is the capacitance of the 3000Å SiO_2 dielectric (1.15×10^{-9} F/cm^2), and V_{TH} is the threshold voltage. We now show three devices, each with a different saturation regime.

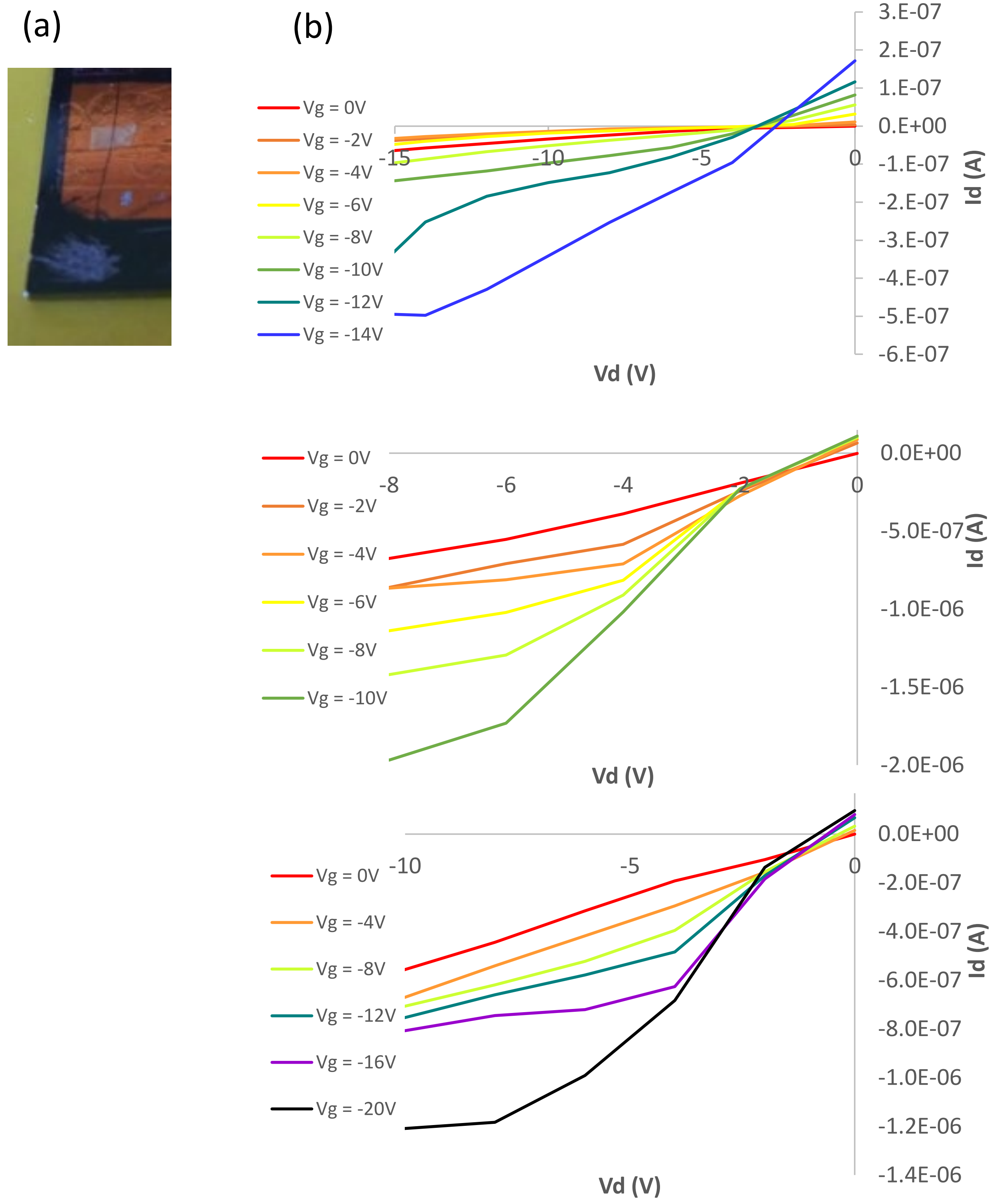

Figure S13. (a) Image of the fabricated Si_6C_6 thin-film transistor. (b) I_d-V_d curves of the transistors.

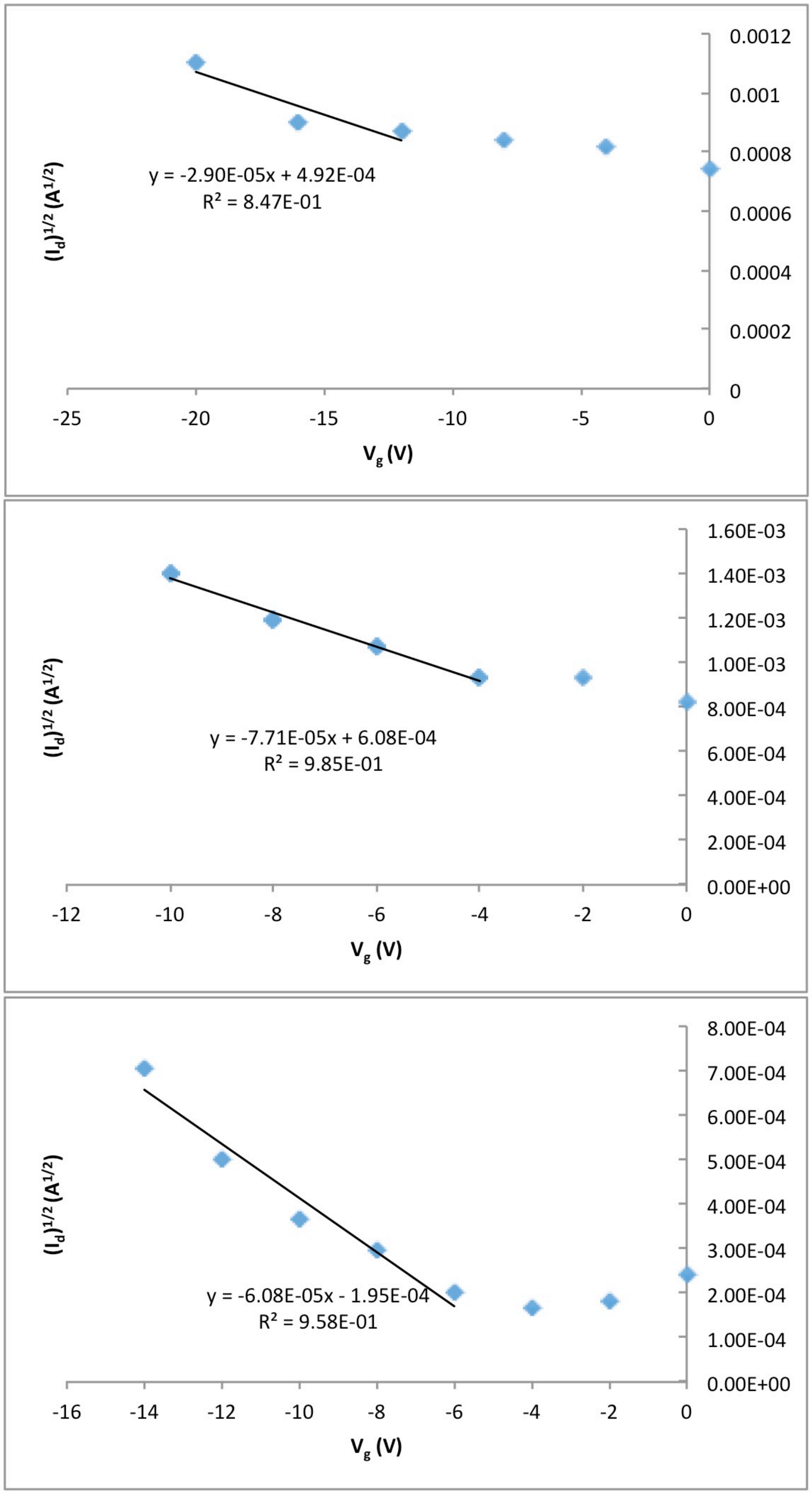

Figure S14. $I_d^{1/2}$-V_g curves for the Si_6C_6 transistors.

Table S5. W/L ratio and mobility of Si_6C_6 transistors

Device	W/L	Mobility ($cm^2V^{-1}s^{-1}$)
#1	10.7	6.0×10^{-2}
#2	18.7	5.5×10^{-2}
#3	13.3	1.1×10^{-2}

11. Computational Methods and Coordinates

All DFT calculations were done using Gaussian 09.[10] We optimized the geometry of each molecule using the B3LYP functional and the 6-31G basis set. At the optimized geometry, we calculated frontier orbital energies using the 6-31G basis set.

1. C_6C_1 Coordinates

C	7.18975	-0.5349	-0.40547
C	6.55581	-0.58154	0.8552
C	5.24117	-1.0157	0.96567
C	4.50127	-1.41884	-0.15775
C	5.12984	-1.37486	-1.41046
C	6.44416	-0.94301	-1.53243
C	3.05936	-1.85049	-0.02486
C	2.06733	-0.67451	-0.15232
C	0.60346	-1.11306	-0.02261
C	-0.39041	0.04814	-0.15522
C	-1.85555	-0.39068	-0.03099
C	-2.83189	0.78333	-0.16506
C	-4.31195	0.4581	-0.08064
C	-4.80171	-0.82601	0.18829
C	-6.17064	-1.06473	0.25944
C	-7.11043	-0.033	0.06472
C	-6.6146	1.26243	-0.20696
C	-5.24884	1.48969	-0.27584
C	8.55616	-0.10427	-0.64134
C	-8.51348	-0.39269	0.15918
C	9.53065	0.35095	0.1948
C	-9.65732	0.33528	0.02489
C	10.84632	0.70813	-0.42349
O	11.09492	0.61662	-1.60913
O	11.72211	1.14434	0.49949
C	13.01832	1.51164	-0.00143
C	9.38763	0.51158	1.60634
N	9.2633	0.64027	2.75715
C	-10.95282	-0.39501	0.19366
O	-11.04547	-1.58109	0.4401
O	-12.00598	0.4273	0.03791
C	-13.29762	-0.18627	0.18253
C	-9.70805	1.73279	-0.26336
N	-9.74161	2.87291	-0.49874
H	7.09041	-0.28393	1.74876
H	4.7765	-1.04889	1.94837
H	4.58292	-1.68811	-2.29666
H	6.91444	-0.92077	-2.51247
H	2.90959	-2.34031	0.94606
H	2.82645	-2.59857	-0.79386
H	2.22094	-0.17827	-1.12054
H	2.29922	0.07634	0.61554
H	0.45674	-1.60939	0.94795

H	0.3818	-1.87101	-0.78844
H	-0.24075	0.54583	-1.12452
H	-0.1705	0.80471	0.61215
H	-2.00181	-0.88702	0.93845
H	-2.07456	-1.14371	-0.80093
H	-2.64716	1.29497	-1.12109
H	-2.59936	1.53098	0.60816
H	-4.11454	-1.6512	0.34372
H	-6.52718	-2.07023	0.4693
H	-7.29664	2.08875	-0.36479
H	-4.89166	2.49521	-0.48726
H	8.87756	-0.14643	-1.68063
H	-8.70426	-1.44189	0.37833
H	13.58297	1.83889	0.87138
H	12.93007	2.32046	-0.73134
H	13.50128	0.65317	-0.47537
H	-14.01782	0.61569	0.02156
H	-13.40834	-0.61074	1.18376
H	-13.42858	-0.97909	-0.55846

2. Si_6C_1 Coordinates

C	-7.90141000	-1.15749000	0.83981300
C	-6.55448800	-0.86740700	1.04486300
C	-6.02182500	0.39646800	0.71348700
C	-6.90658600	1.35046400	0.16494100
C	-8.25605700	1.07023900	-0.04566200
C	-8.78589900	-0.19818200	0.28982000
H	-8.28811600	-2.13717500	1.10736300
H	-5.91333400	-1.63169800	1.47365200
H	-6.53846800	2.33695800	-0.10008800
H	-8.89169000	1.83824100	-0.46580500
C	-10.17172500	-0.60661100	0.11945000
H	-10.38375000	-1.61988900	0.44815500
C	-11.26029800	0.05277200	-0.38031900
C	-11.24357600	1.39039000	-0.87855400
C	-12.58780100	-0.60868600	-0.44318500
C	-13.85161100	-2.61540000	0.03027700
H	-14.21159300	-2.70168100	-0.99690800
H	-14.59458800	-2.08648000	0.63062100
H	-13.62566700	-3.59222900	0.45300600
O	-12.57202800	-1.89186300	0.05839900
Si	-4.15006900	0.79139400	0.96592100
Si	-3.01074000	0.09690400	-1.03083300
Si	-0.70233300	0.75121100	-1.21733500
Si	0.71674900	-0.00450800	0.57375100
Si	3.01392900	-0.09849900	-0.13270900
Si	4.55020500	-0.44981500	1.68440900
C	6.30534200	-0.66569100	0.91496900
C	7.14447400	0.44391200	0.67448300
C	6.78546200	-1.93437100	0.52771500
C	8.40649300	0.30578100	0.09736500
H	6.80837800	1.44065100	0.94516800
C	8.03611300	-2.07976200	-0.06803200
H	6.17582600	-2.81864600	0.68710100

C	8.88855500	-0.96750800	-0.27995500
H	9.02218600	1.17746300	-0.06236400
H	8.37330800	-3.06781700	-0.36977400
C	10.15563500	-1.25536000	-0.94573300
H	10.13182000	-2.22796000	-1.43659100
C	11.35496200	-0.61313400	-1.12334300
C	11.88302400	0.64956900	-0.55176600
C	12.30954700	-1.27379400	-1.97139600
O	12.94714400	1.16853900	-0.91559200
O	-13.61343600	-0.08281000	-0.89269300
N	-11.22239500	2.49141800	-1.28719200
N	13.06007000	-1.84473700	-2.67098500
O	11.12566500	1.17930600	0.46355100
C	11.67048100	2.37959400	1.12117100
H	10.92716300	2.64159800	1.87130500
H	12.63227700	2.14604300	1.58162300
H	11.80347600	3.18113800	0.39212800
C	-3.55217600	-0.17381000	2.52046300
H	-3.60454600	-1.25774600	2.37181900
H	-2.51338100	0.08101400	2.75673600
H	-4.17180200	0.08305600	3.38728800
C	-3.96236300	2.67869900	1.28624400
H	-4.26272700	3.27202200	0.41611100
H	-4.57684400	2.98599900	2.14017500
H	-2.91813000	2.92290200	1.51154600
C	-3.95056900	0.87412000	-2.53740300
H	-5.00943600	0.59329000	-2.51287900
H	-3.88605100	1.96753300	-2.52868900
H	-3.52302900	0.51495800	-3.48060300
C	-3.16642800	-1.82784600	-1.17431100
H	-2.65427800	-2.33782700	-0.35189400
H	-4.22181800	-2.12423000	-1.16147900
H	-2.72866200	-2.17508400	-2.11727800
C	-0.08370500	0.04667800	-2.91558900
H	0.94079300	0.37779700	-3.12033800
H	-0.09762500	-1.04865000	-2.92384400
H	-0.72324800	0.40228300	-3.73168100
C	-0.58474700	2.68203800	-1.31657700
H	-1.21958600	3.06352600	-2.12450400
H	-0.90282100	3.15327500	-0.38021800
H	0.44705700	2.99026300	-1.52145900
C	0.54119600	1.23036800	2.05672100
H	0.90660300	2.22690600	1.78620800
H	-0.50679500	1.32586800	2.36440200
H	1.11545800	0.87863600	2.92165500
C	0.18259800	-1.76063000	1.19052000
H	-0.85300200	-1.74358300	1.54861300
H	0.25475700	-2.50137100	0.38699300
H	0.82173500	-2.09115100	2.01737400
C	3.24848700	-1.55473200	-1.38702100
H	2.94529900	-2.51110800	-0.94649700
H	2.64797600	-1.38917500	-2.28812400
H	4.30009800	-1.63320500	-1.68502200
C	3.52277900	1.55037600	-1.01317300
H	3.38359400	2.41387300	-0.35346100

H	4.57690200	1.51309900	-1.31127000
H	2.92160100	1.71053200	-1.91575700
C	4.59773500	1.05335100	2.88516000
H	4.79847300	1.99145200	2.35686700
H	3.63687900	1.16026000	3.40022300
H	5.37782100	0.91174600	3.64196500
C	4.13033500	-2.03812400	2.68690800
H	3.16749600	-1.92249500	3.19723500
H	4.06325500	-2.92213100	2.04358000
H	4.89817700	-2.22450500	3.44658900

12. References

1. Klausen, R. S.; Widawsky, J. R.; Steigerwald, M. L.; Venkataraman, L.; Nuckolls, C. *J. Am. Chem. Soc.* **2012,** *134* (10), 4541-4544.

2. Peukert, S.; Sun, Y.; Zhang, R.; Hurley, B.; Sabio, M.; Shen, X.; Gray, C.; Dzink-Fox, J.; Tao, J.; Cebula, R.; Wattanasin, S. *Bioorg. Med. Chem. Lett.* **2008,** *18* (6), 1840-1844.

3. Hayashi, T. *J. Org. Chem.* **1966,** *31* (10), 3253-&.

4. Hu, Y.; Gao, X.; Di, C.-a.; Yang, X.; Zhang, F.; Liu, Y.; Li, H.; Zhu, D. *Chem. Mater.* **2011,** *23* (5), 1204-1215.

5. Li, P.; Sisto, T. J.; Darzi, E. R.; Jasti, R. *Org. Lett.* **2014,** *16* (1), 182-185.

6. Shibano, Y.; Sasaki, M.; Tsuji, H.; Araki, Y.; Ito, O.; Tamao, K. *J. Organomet. Chem.* **2007,** *692* (1-3), 356-367.

7. Mott, N. F. and Gurney, R. F. *Electronic Processes In Ionic Crystals*. 2nd ed. Oxford: Clarendon Press, 1948.

8. A relative permittivity of 2.5 is assumed by comparison to literature values reported for 1,4-dicyanobenzene (2.8) and organosilicate films (2.3-3.2). 1,4-Dicyanobenzene: Huber, H.; Wright, G. F. *Can. J. Chem*. **1973**, *51*, 2234. Organosilicate films: (a) Lee, P.-C.; Kim, S.; Hwang, T.; Oh, J.-S.; Oh, Y.-S.; Pu, L.; Kim, B.-W.; Lee, Y.; Choi, H. R.; Jeoung, S. K.; Nam, J.-D. *J. Mater. Sci*. **2012**, *47*, 4540. (b) Volksen, W.; Miller, R. D.; DuBois, G. *Chem. Rev*. **2010**, *110*, 56. (c) Coclite, A. M.; Milella, A.; Palumbo, F.; Fracassi, F.; d'Agostino, R. *Plasma Process. Polym*. **2009**, *6,* 512. (d) Knoesen, A.; Song, G.; Volksen, W.; Huang, E.; Magbitang, T.; Sundberg, L.; Hedrick, J. L.; Hawker, C.; Miller, R. D. *Elec. Mater*. **2004**, *33*, 135.

9. Sheldrick, G. M. *Acta Crystallogr., Sect. A: Cryst. Phys., Diffr., Theor. Gen. Crystallogr.* **2008**, *64*, 112.

10. M. J. Frisch, G. W. Trucks, H. B. Schlegel, G. E. Scuseria, M. A. Robb, J. R. Cheeseman, G. Scalmani, V. Barone, B. Mennucci, G. A. Petersson, H. Nakatsuji, M. Caricato, X. Li, H. P. Hratchian, A. F. Izmaylov, J. Bloino, G. Zheng, J. L. Sonnenberg, M. Hada, M. Ehara, K., Toyota, R. Fukuda, J. Hasegawa, M. Ishida, T. Nakajima, Y. Honda, O. Kitao, H. Nakai, T. Vreven, J. A. Montgomery, Jr., J. E. Peralta, F. Ogliaro, M. Bearpark, J. J. Heyd, E. Brothers, K. N. Kudin, V. N. Staroverov, T. Keith, R. Kobayashi, J. Normand, K. Raghavachari, A. Rendell, J. C. Burant, S. S. Iyengar, J. Tomasi, M. Cossi, N. Rega, J. M. Millam, M. Klene, J. E. Knox, J. B. Cross, V. Bakken, C. Adamo, J. Jaramillo, R. Gomperts, R. E. Stratmann, O. Yazyev, A. J. Austin, R. Cammi, C. Pomelli, J. W. Ochterski, R. L. Martin, K. Morokuma, V. G. Zakrzewski, G. A. Voth, P. Salvador, J. J. Dannenberg, S. Dapprich, A. D. Daniels, O. Farkas, J. B. Foresman, J. V. Ortiz, J. Cioslowski, and D. J. Fox, Gaussian, Inc., Wallingford CT, 2010.

^{1}H NMR, $CDCl_3$, 400 MHz

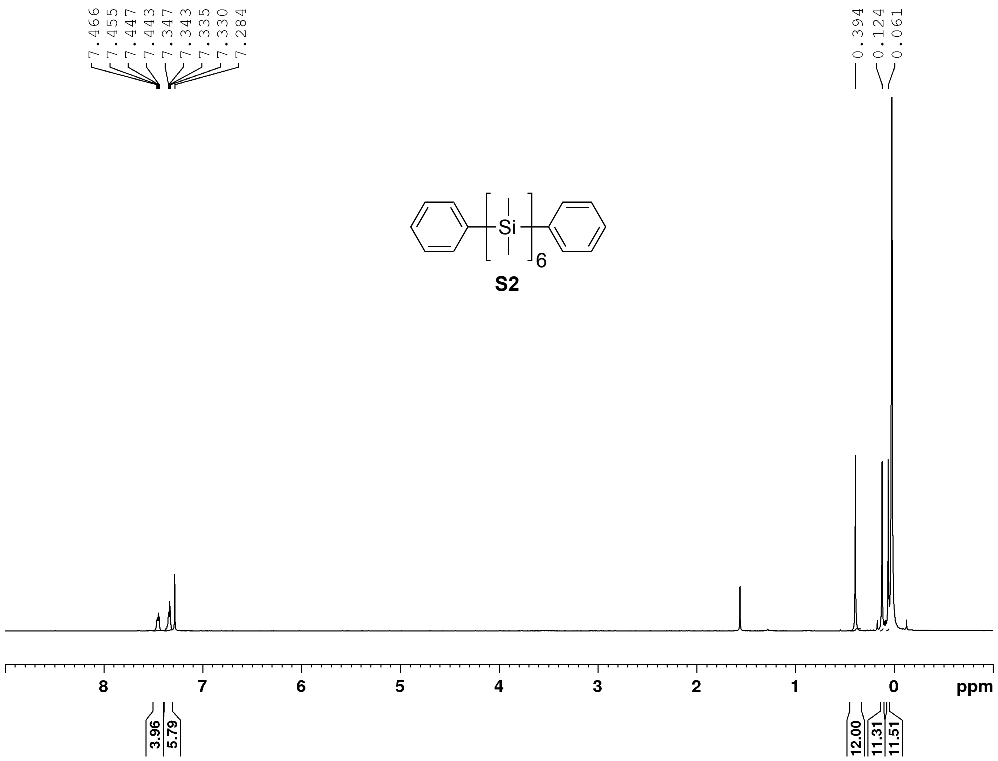

^{13}C NMR, $CDCl_3$, 100 MHz

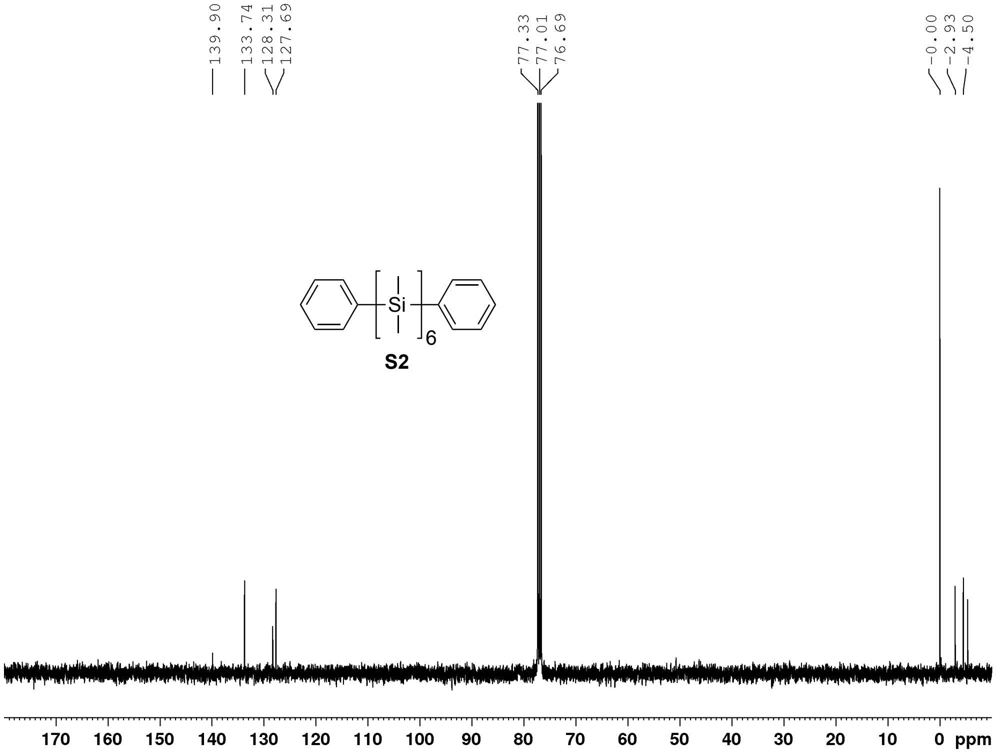

^{29}Si NMR, $CDCl_3$, 80 MHz

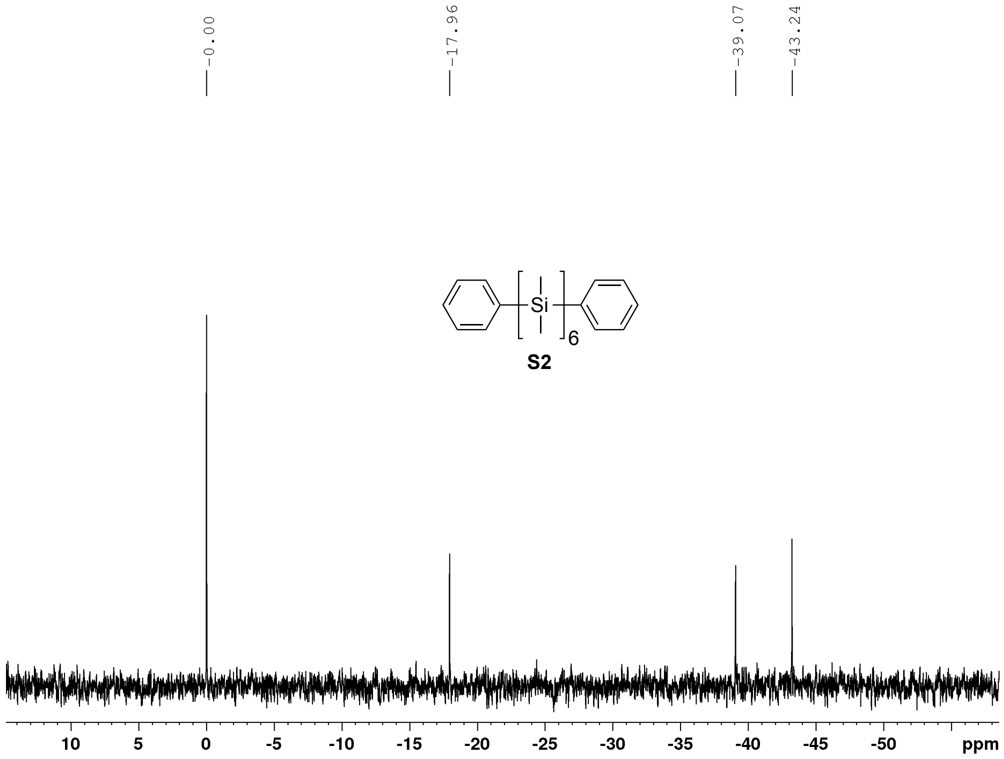

^{1}H NMR, C_6D_6, 400 MHz

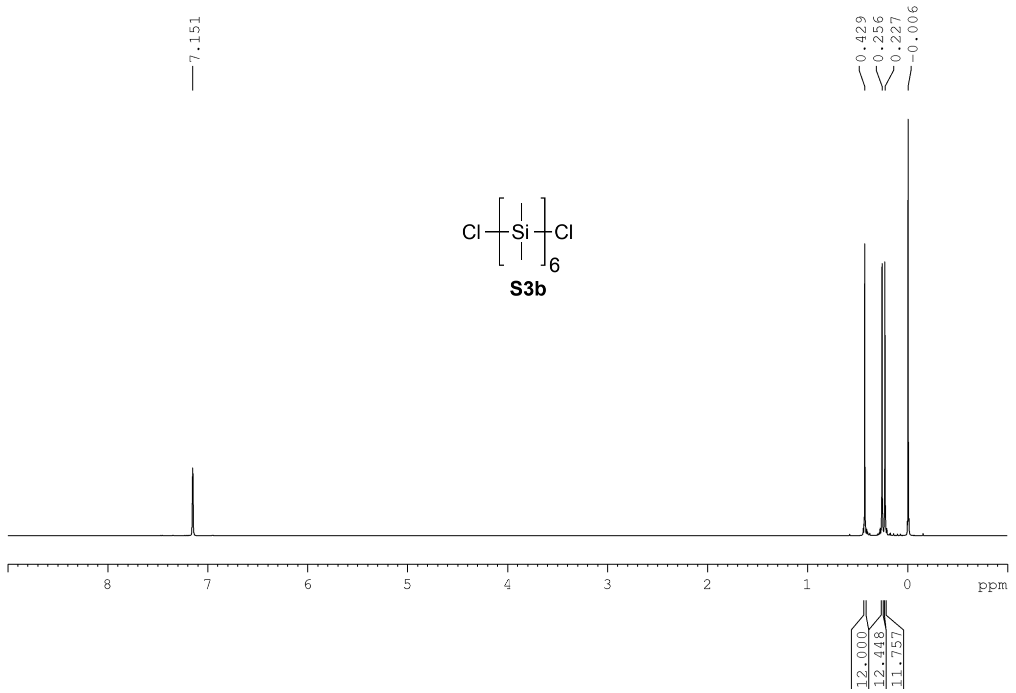

^{1}H NMR, $CDCl_3$, 400 MHz

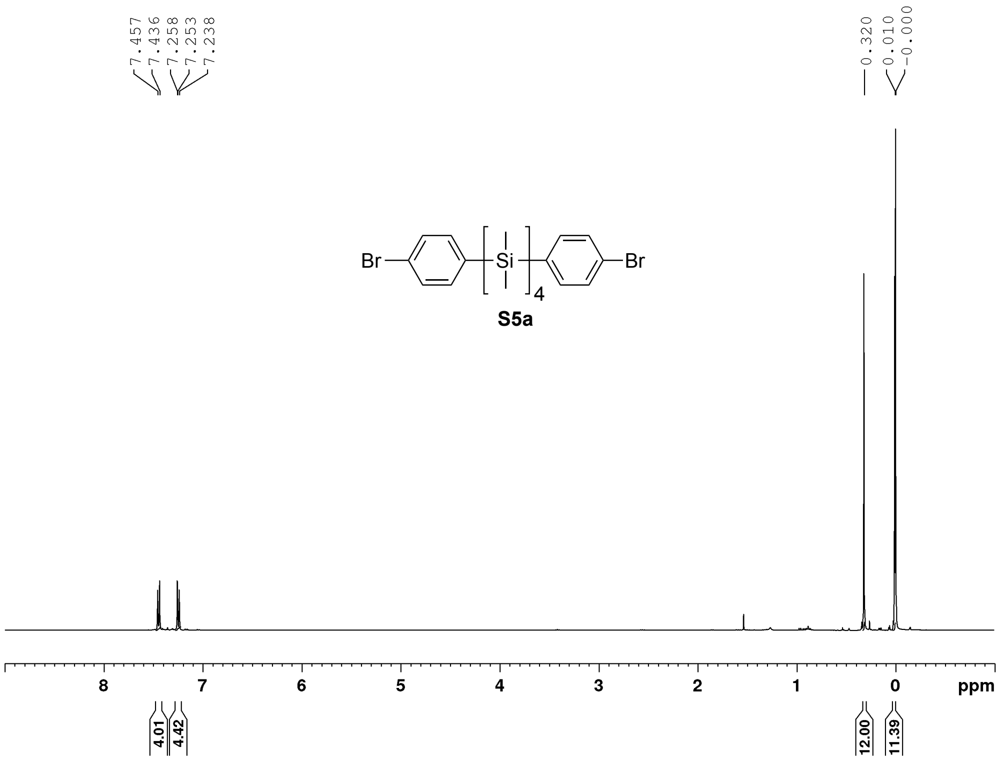

^{13}C NMR, $CDCl_3$, 100 MHz

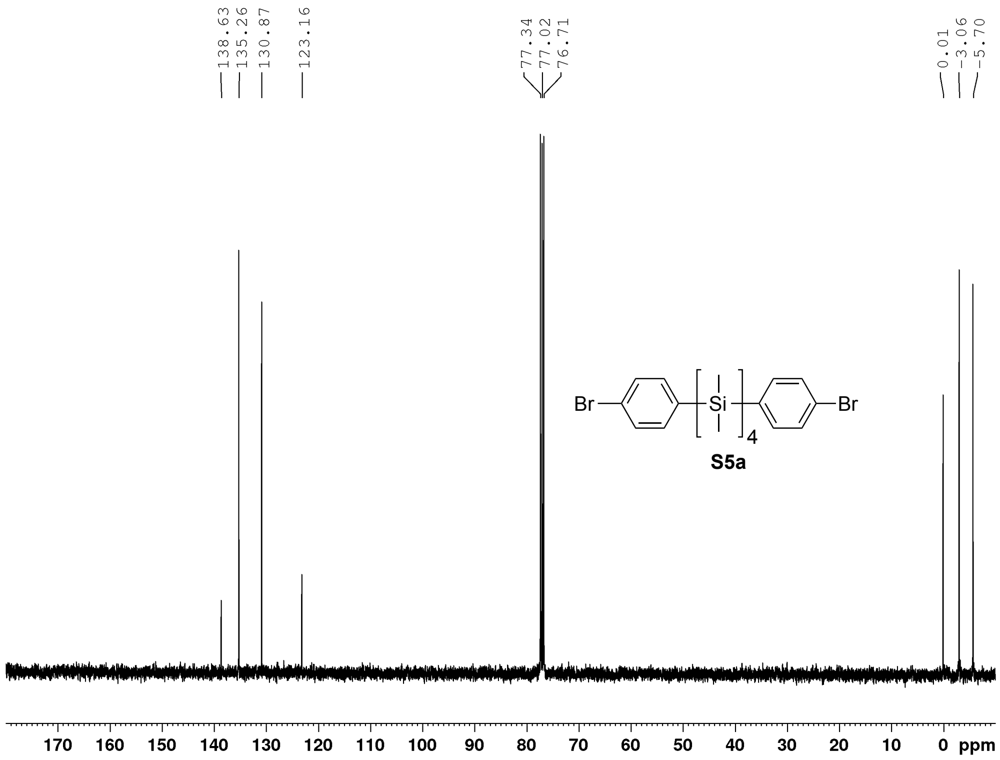

^{29}Si NMR, $CDCl_3$, 80 MHz

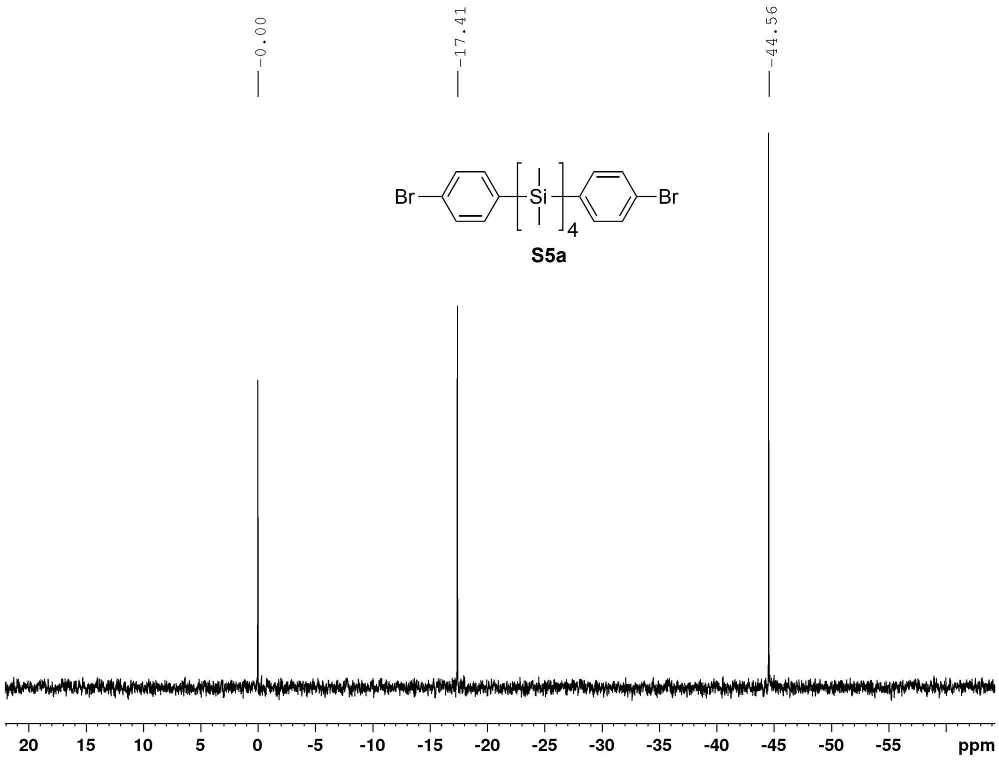

^{1}H NMR, $CDCl_3$, 400 MHz

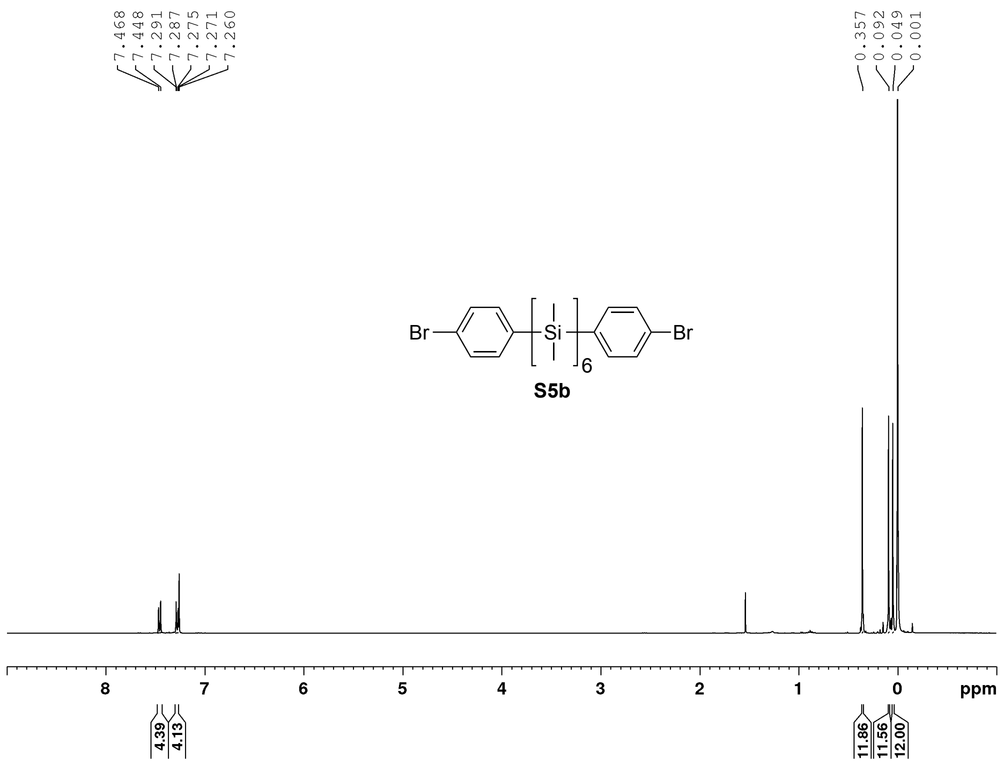

^{13}C NMR, $CDCl_3$, 100 MHz

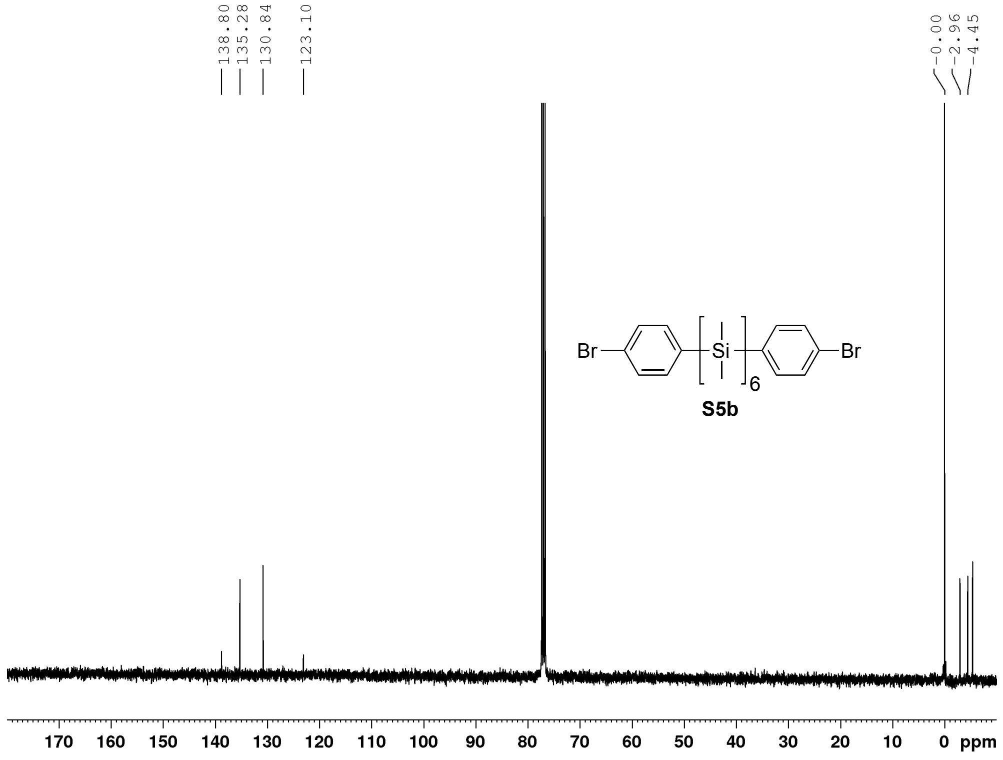

^{29}Si NMR, $CDCl_3$, 80 MHz

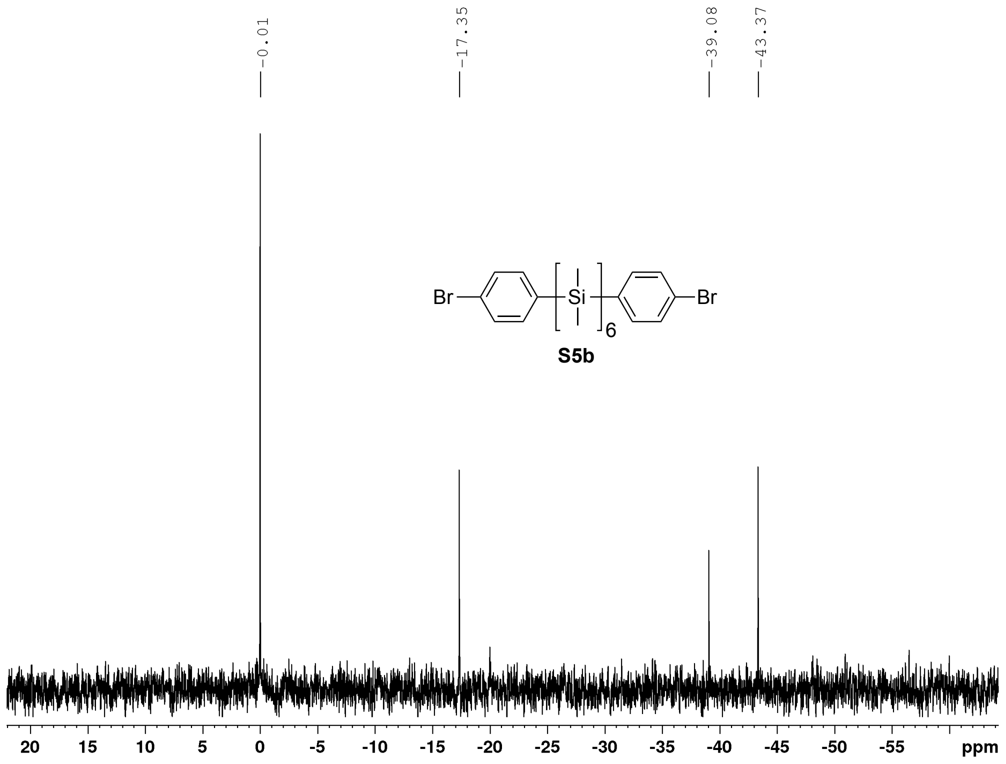

^{1}H NMR, $CDCl_3$, 400 MHz

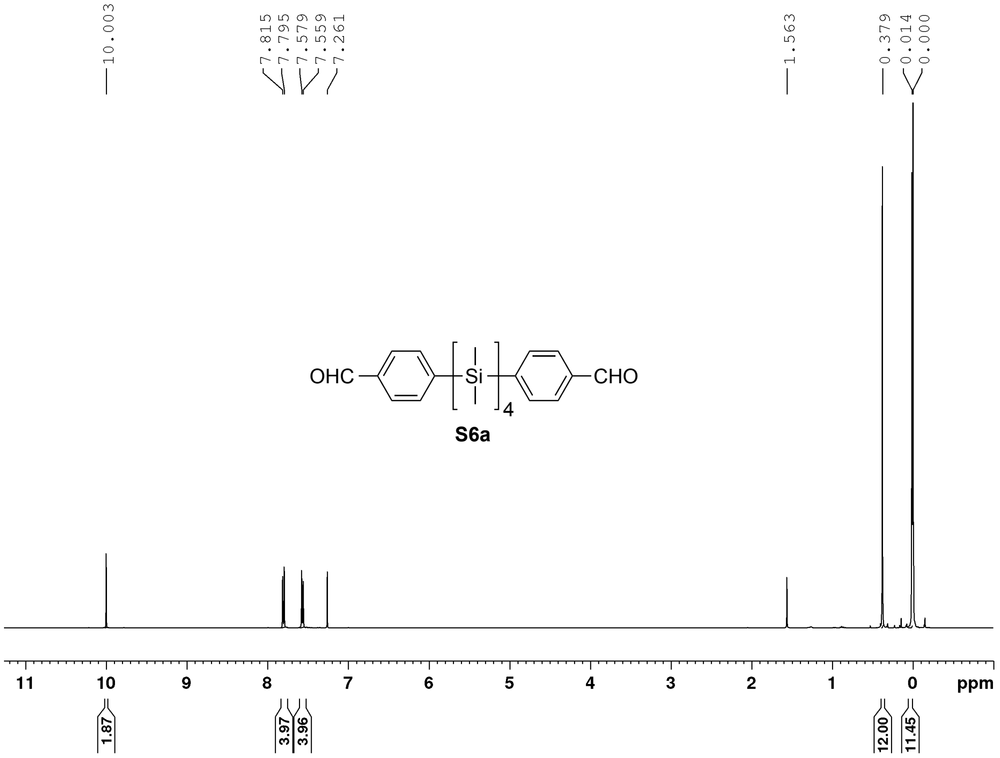

^{13}C NMR, $CDCl_3$, 100 MHz

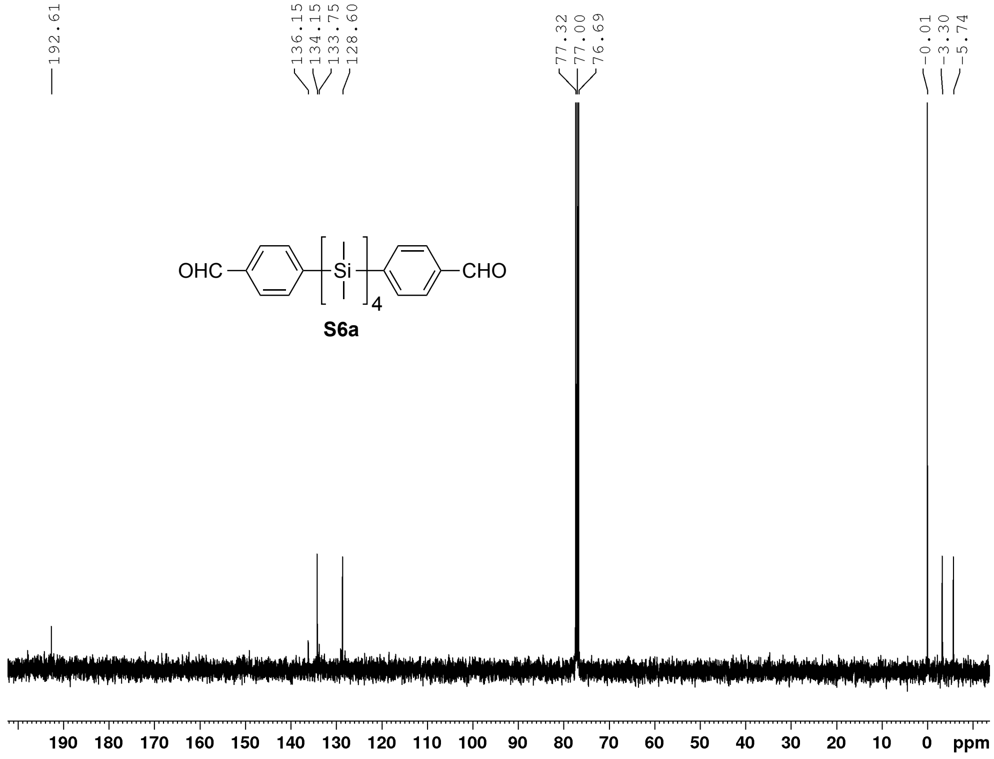

^{29}Si NMR, $CDCl_3$, 80 MHz

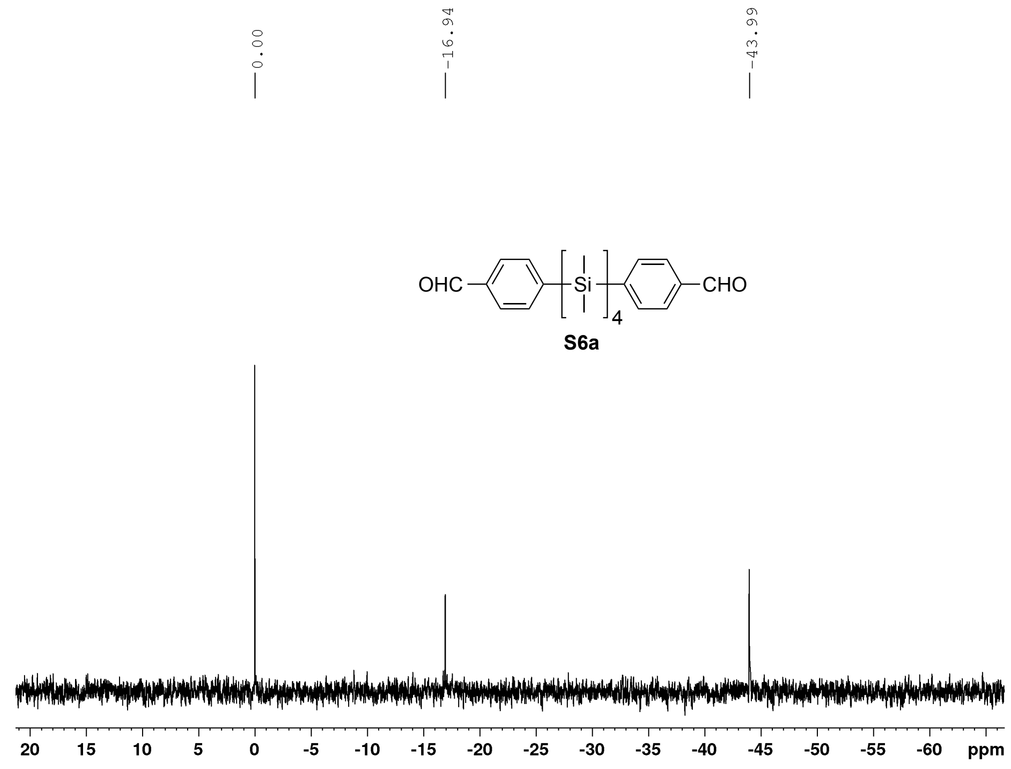

^{1}H NMR, $CDCl_3$, 400 MHz

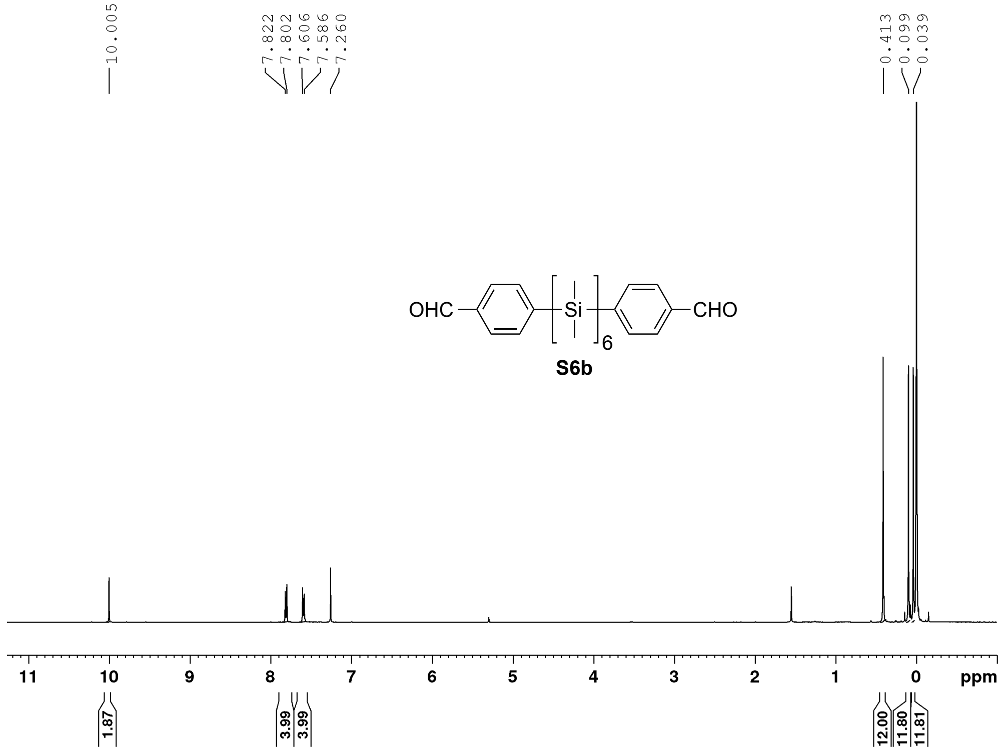

^{13}C NMR, $CDCl_3$, 100 MHz

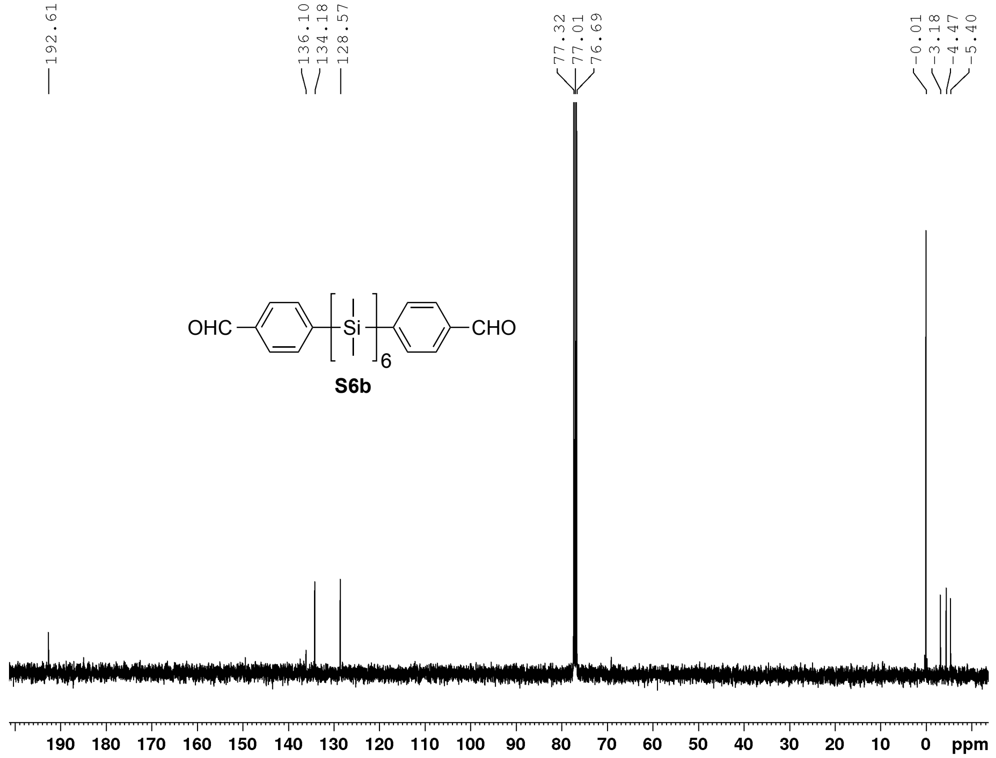

^{29}Si NMR, $CDCl_3$, 80 MHz

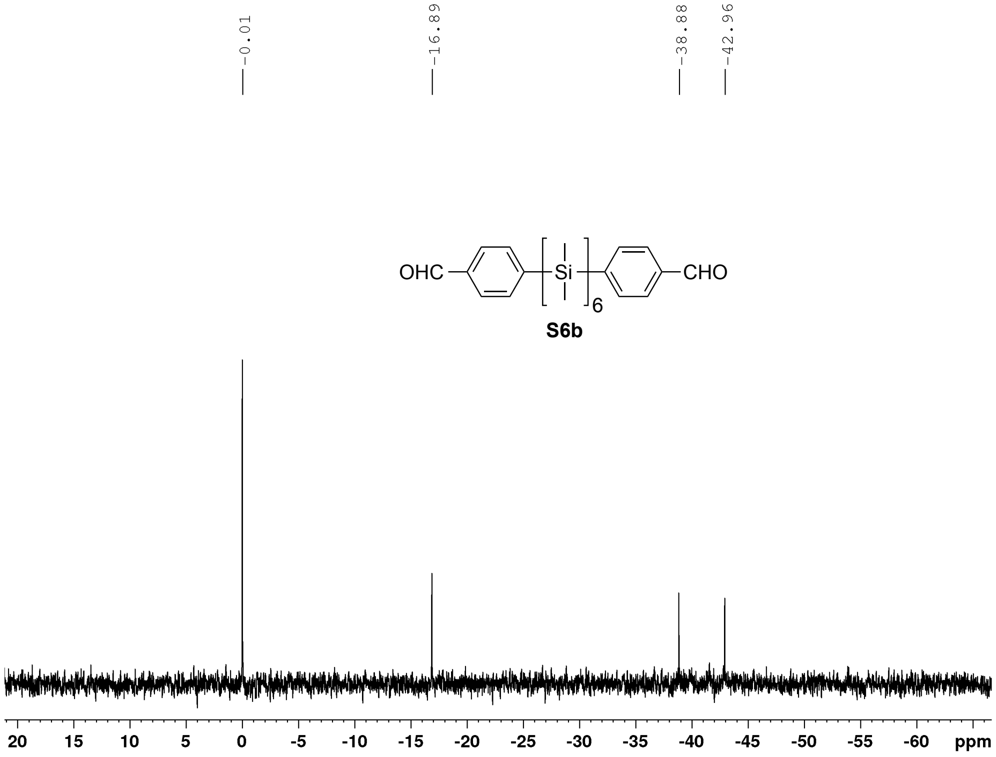

^{1}H NMR, $CDCl_3$, 400 MHz

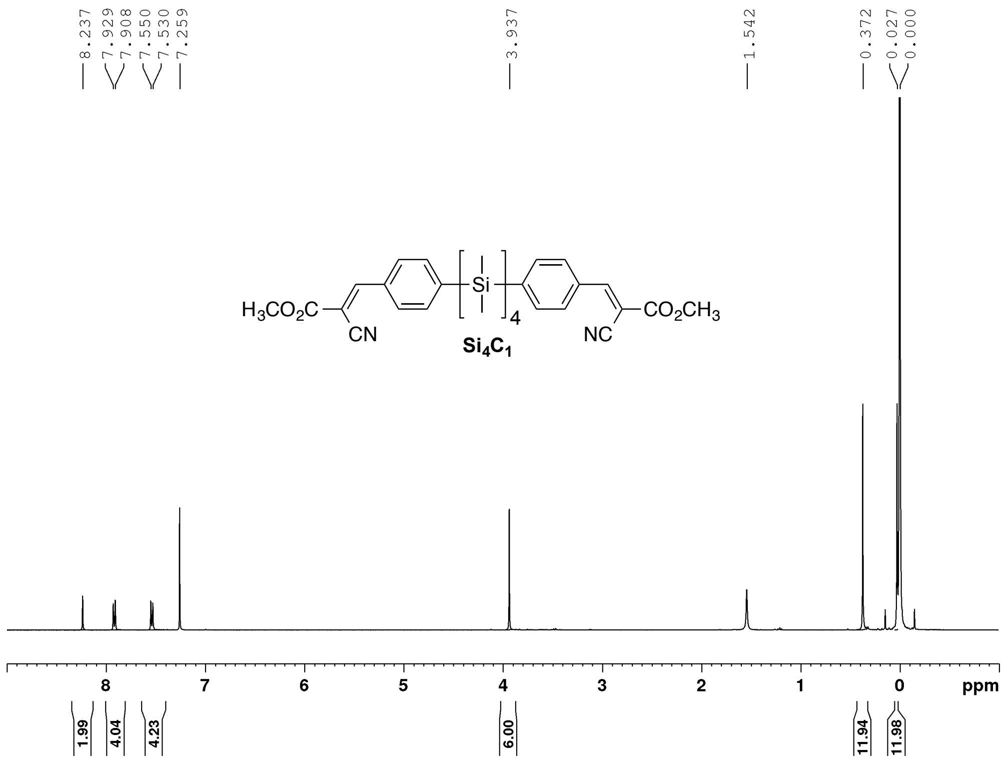

^{13}C NMR, $CDCl_3$, 100 MHz

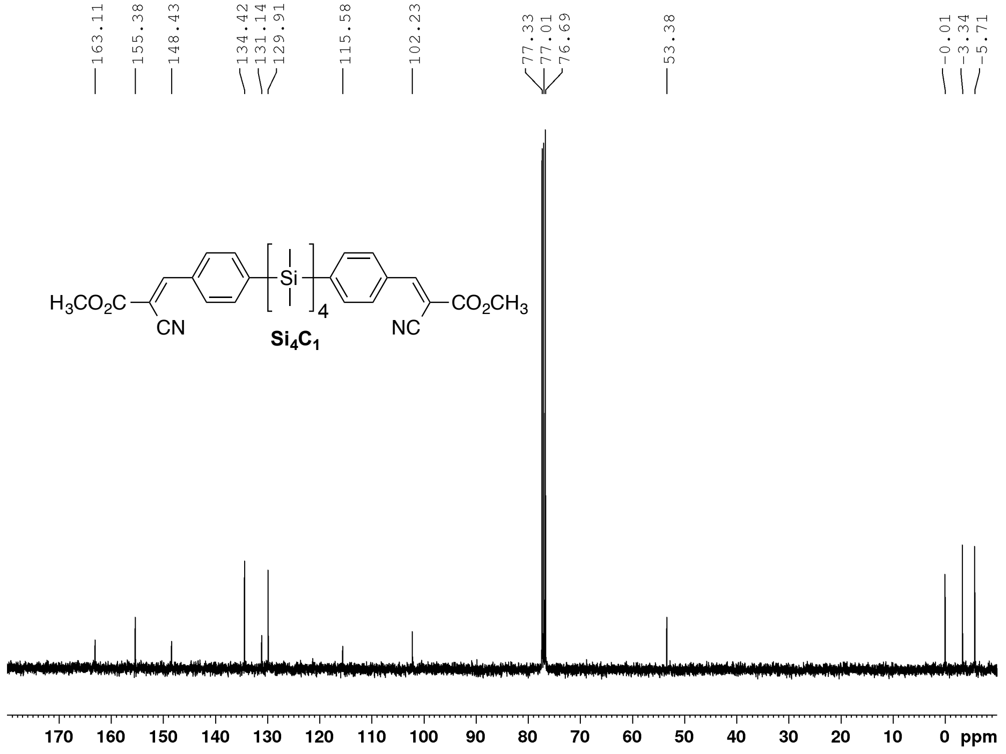

^{29}Si NMR, $CDCl_3$, 80 MHz

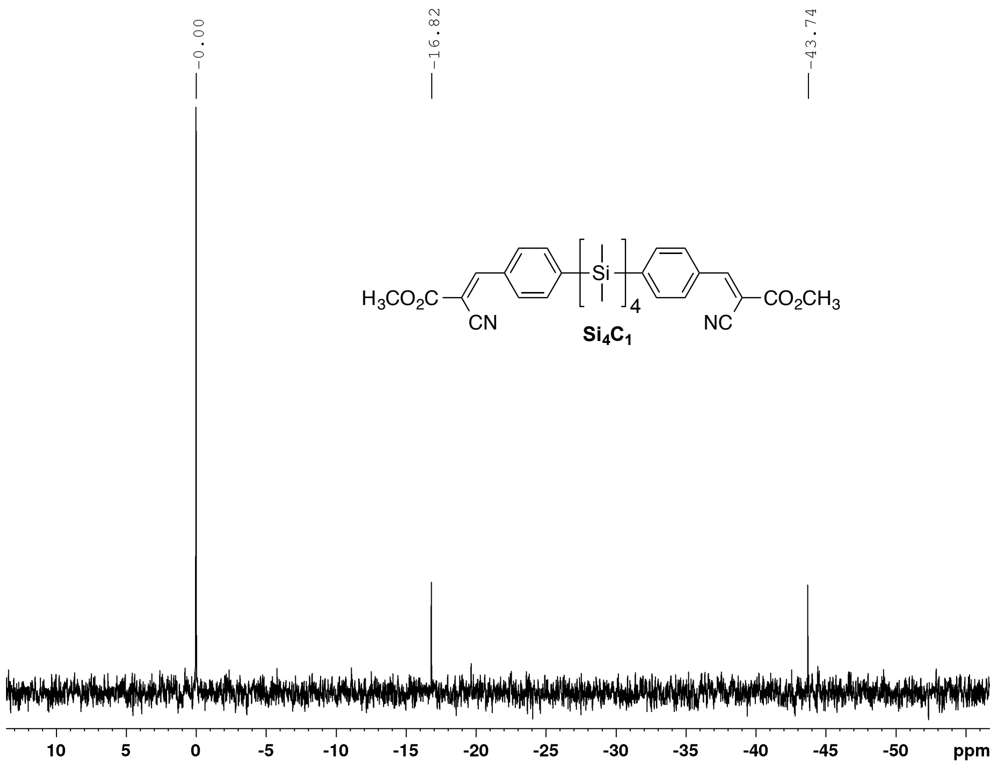

^{1}H NMR, $CDCl_3$, 400 MHz

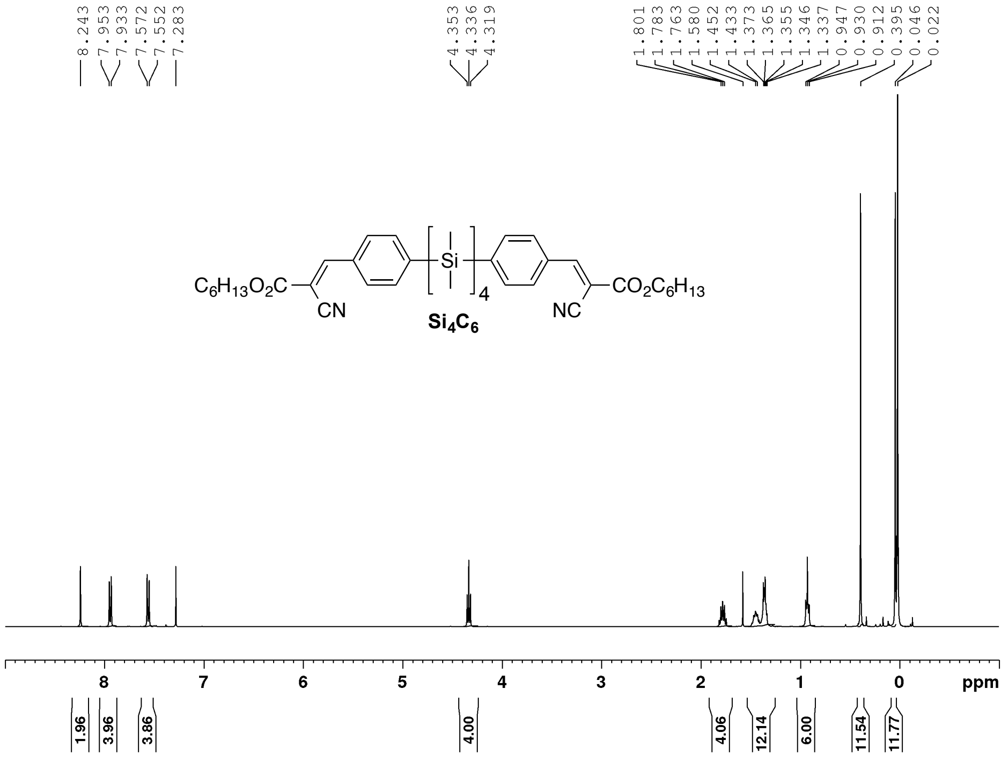

^{13}C NMR, $CDCl_3$, 100 MHz

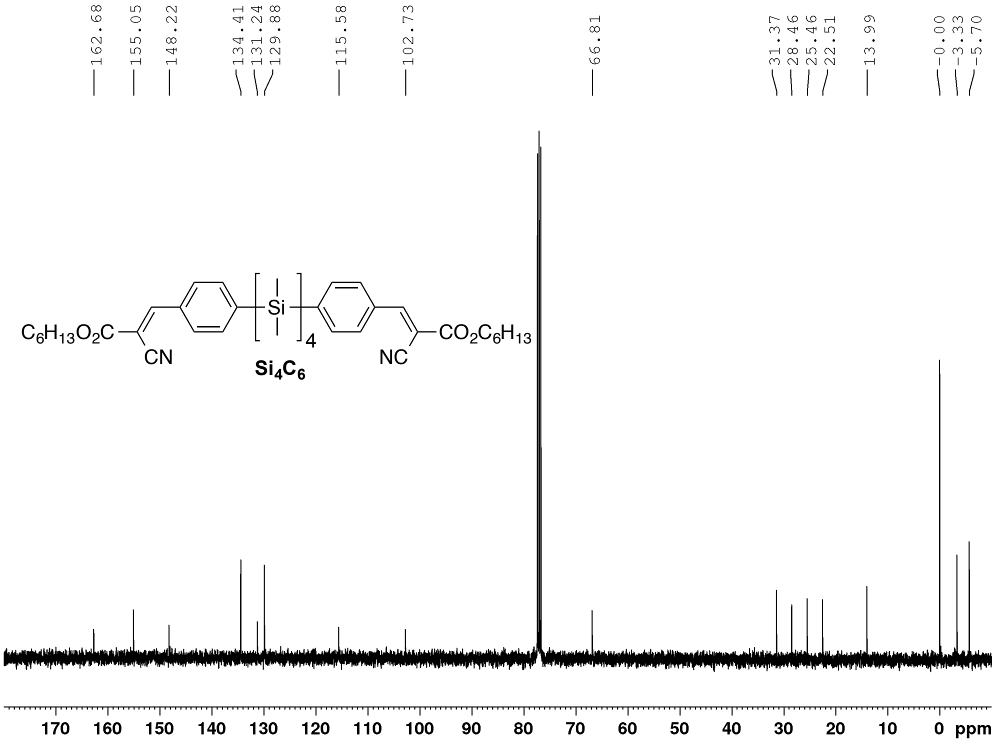

^{29}Si NMR, $CDCl_3$, 80 MHz

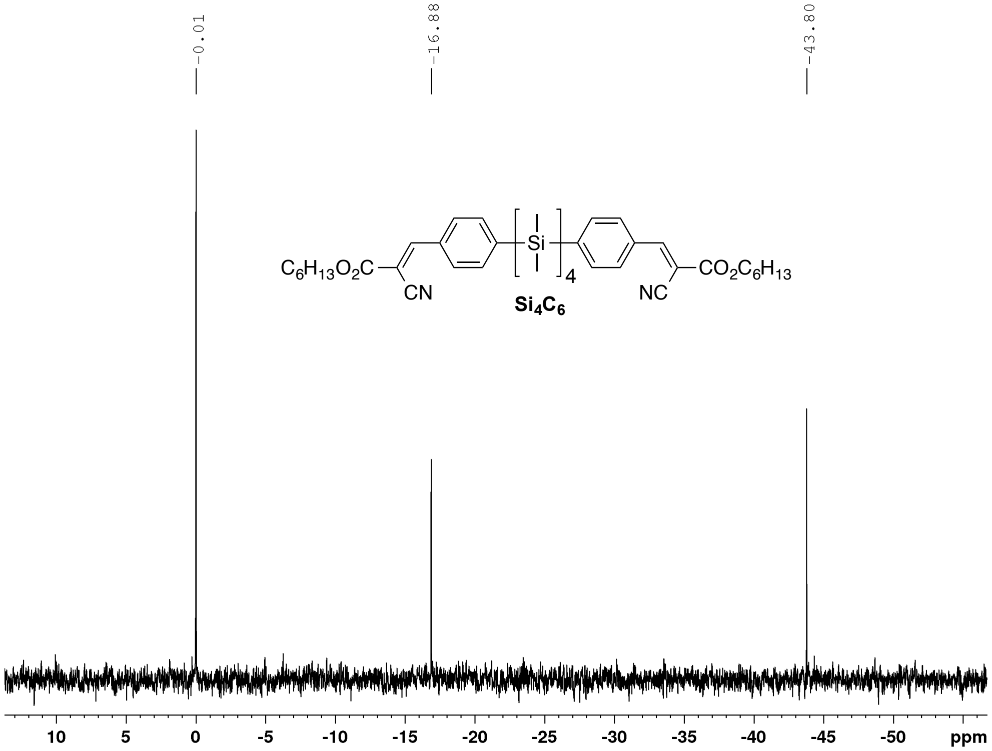

^1H NMR, $CDCl_3$, 400 MHz

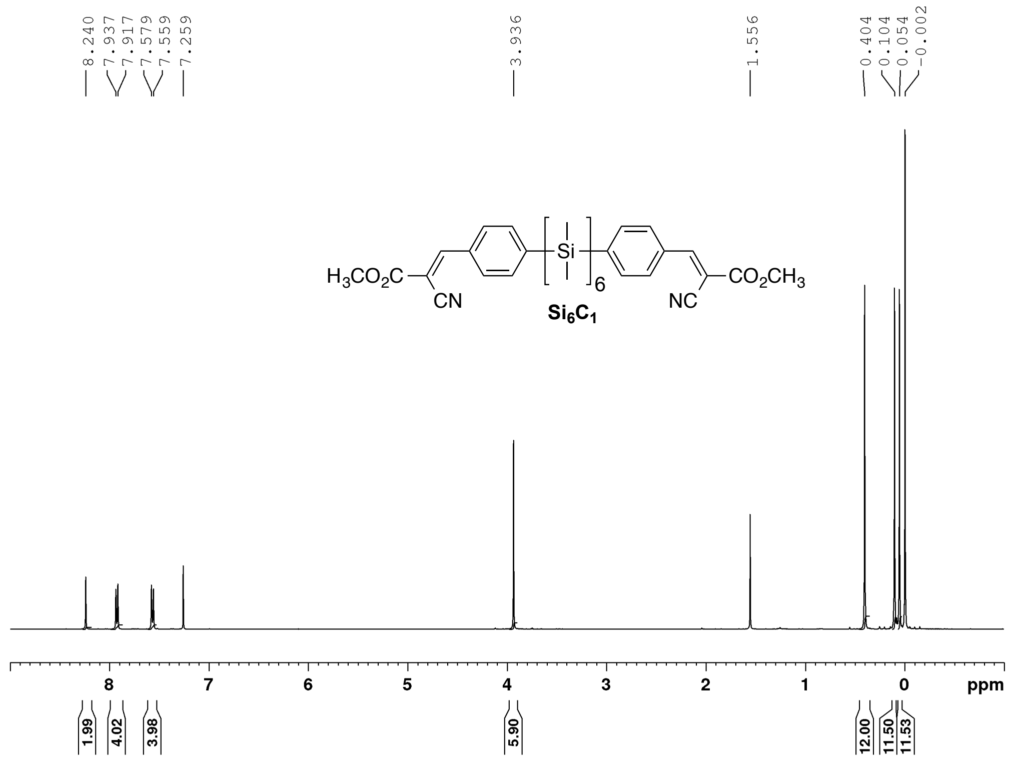

^{13}C NMR, $CDCl_3$, 100 MHz

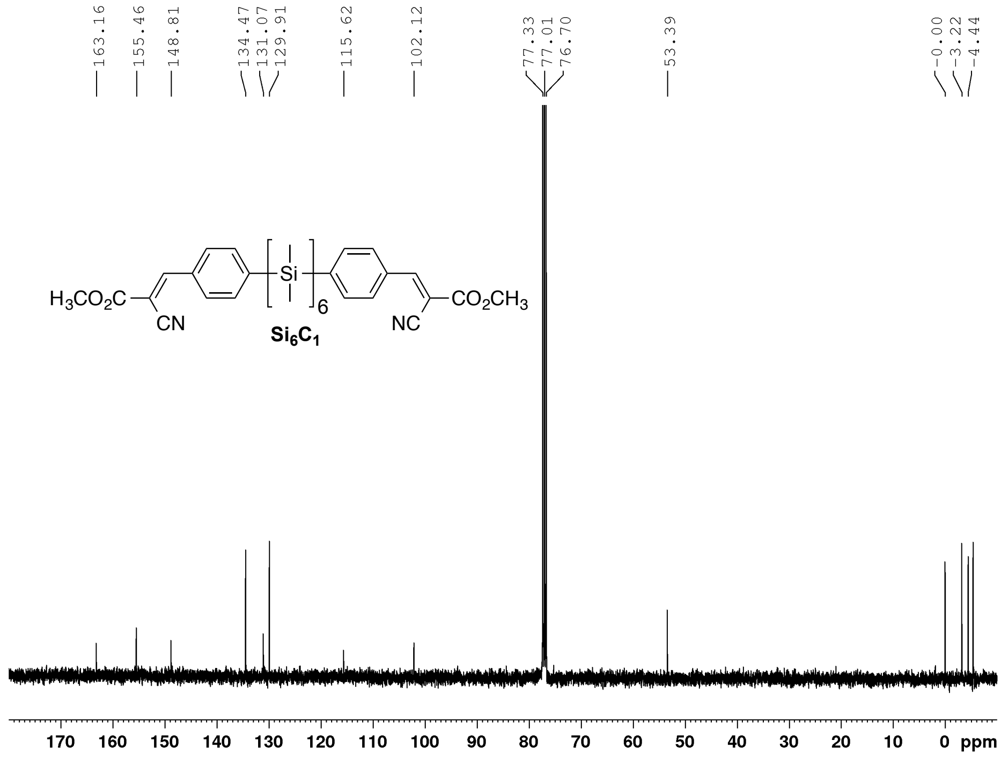

^{29}Si NMR, $CDCl_3$, 80 MHz

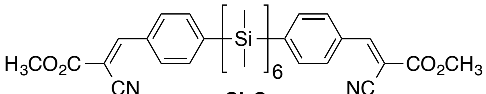

Si_6C_1

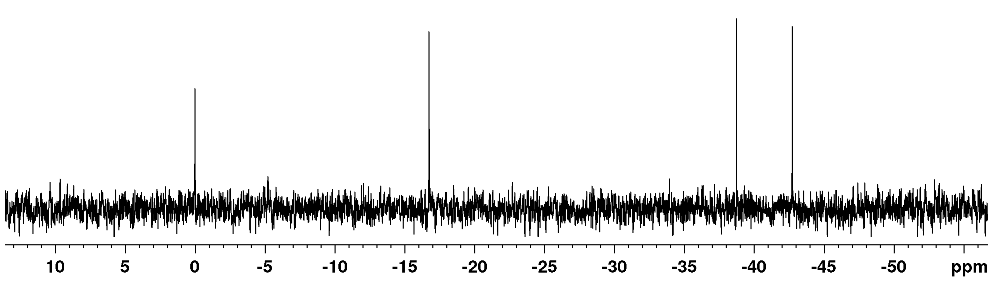

^{1}H NMR, $CDCl_3$, 400 MHz

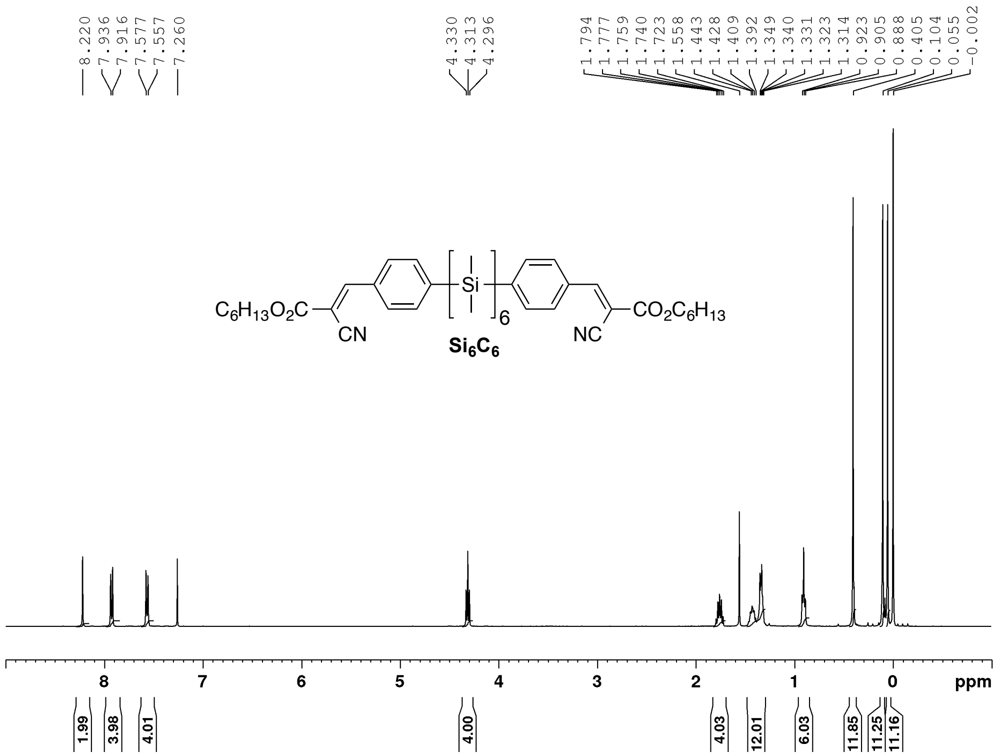

^{13}C NMR, $CDCl_3$, 100 MHz

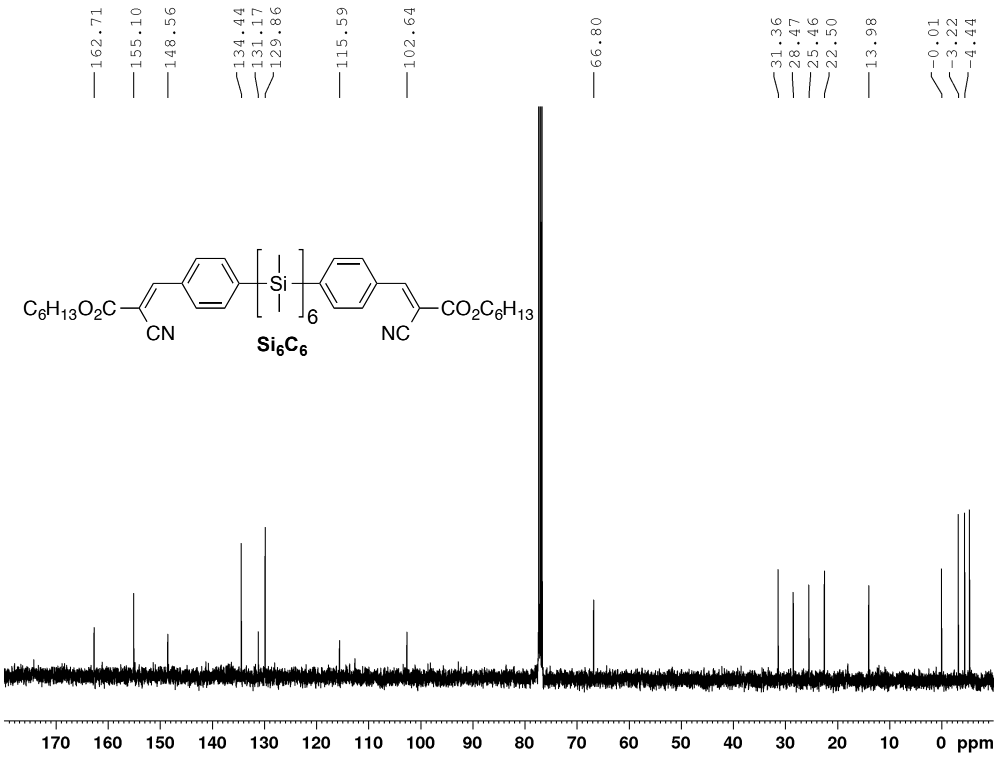

^{29}Si NMR, $CDCl_3$, 80 MHz

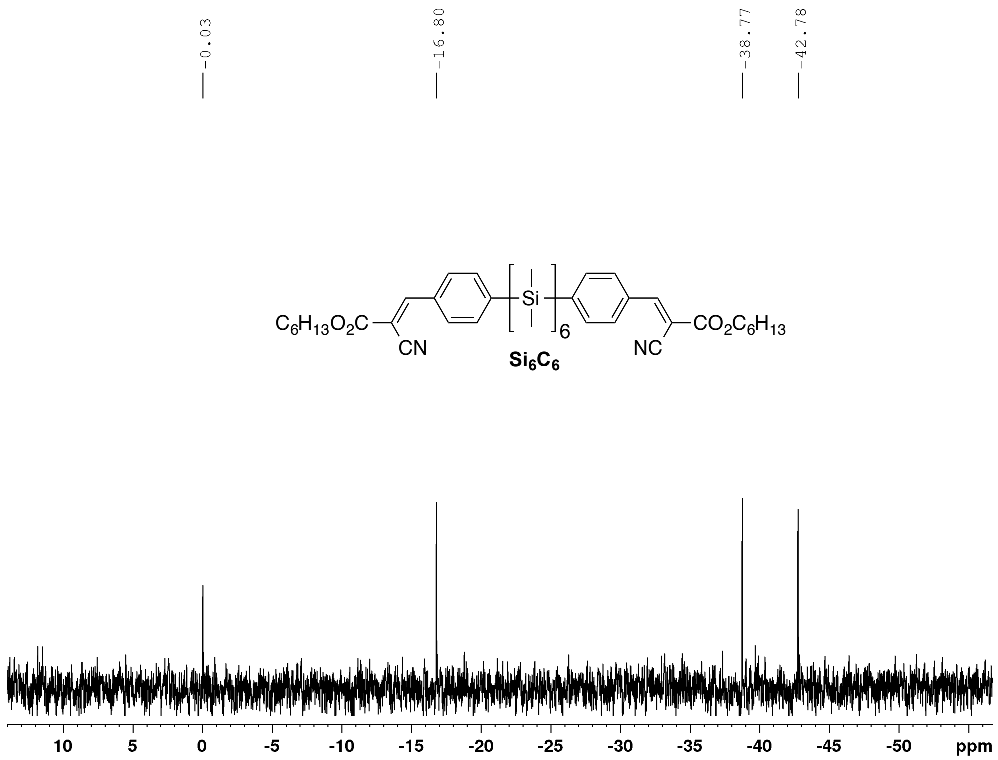

^{1}H NMR, $CDCl_3$, 400 MHz

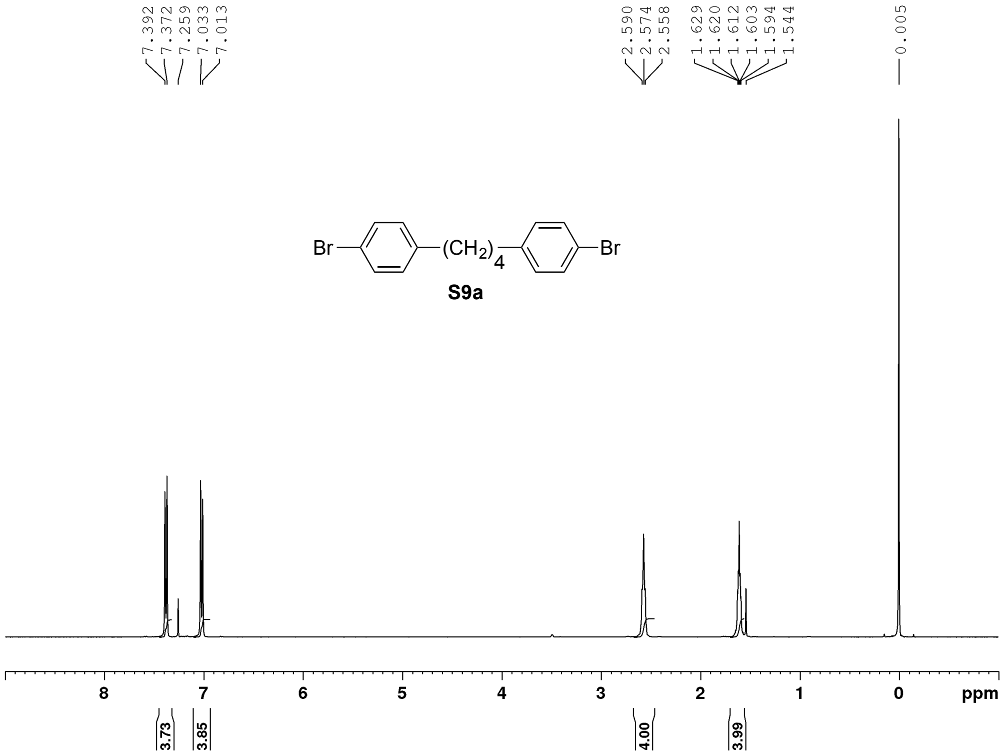

^{13}C NMR, $CDCl_3$, 100 MHz

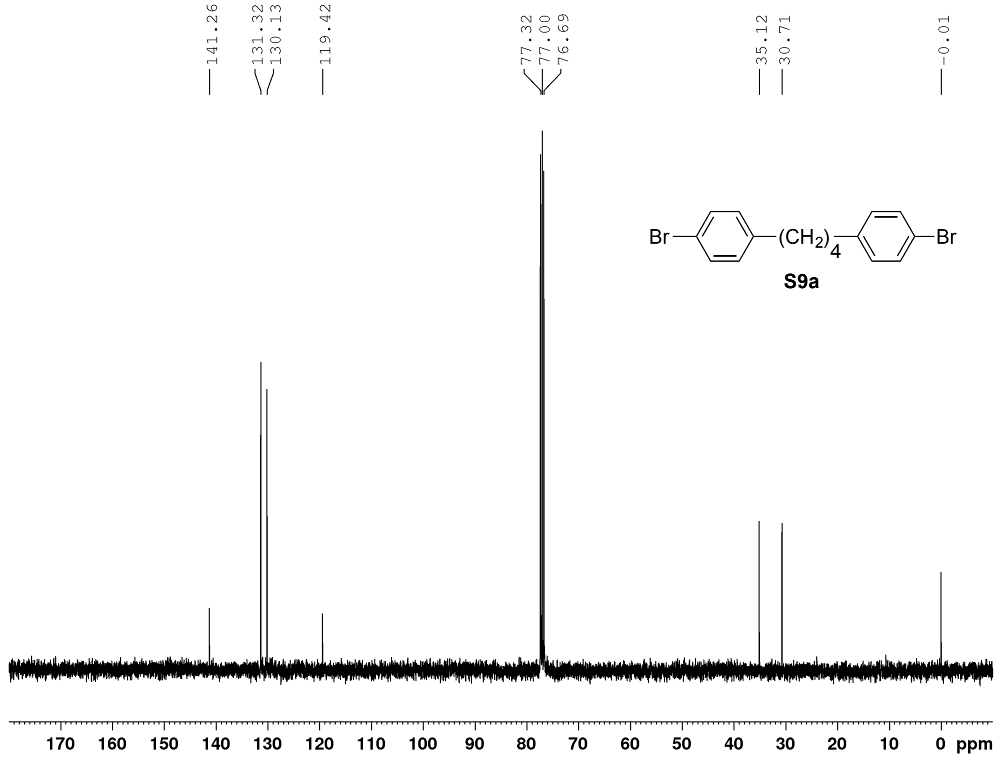

^{1}H NMR, $CDCl_3$, 400 MHz

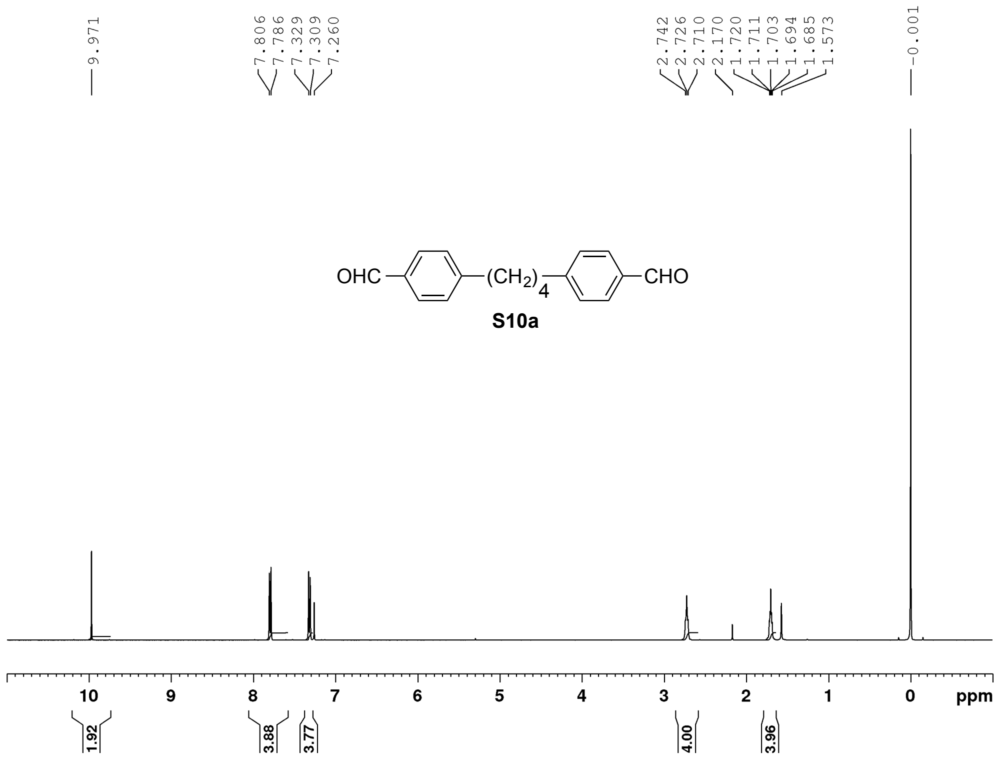

^{13}C NMR, $CDCl_3$, 100 MHz

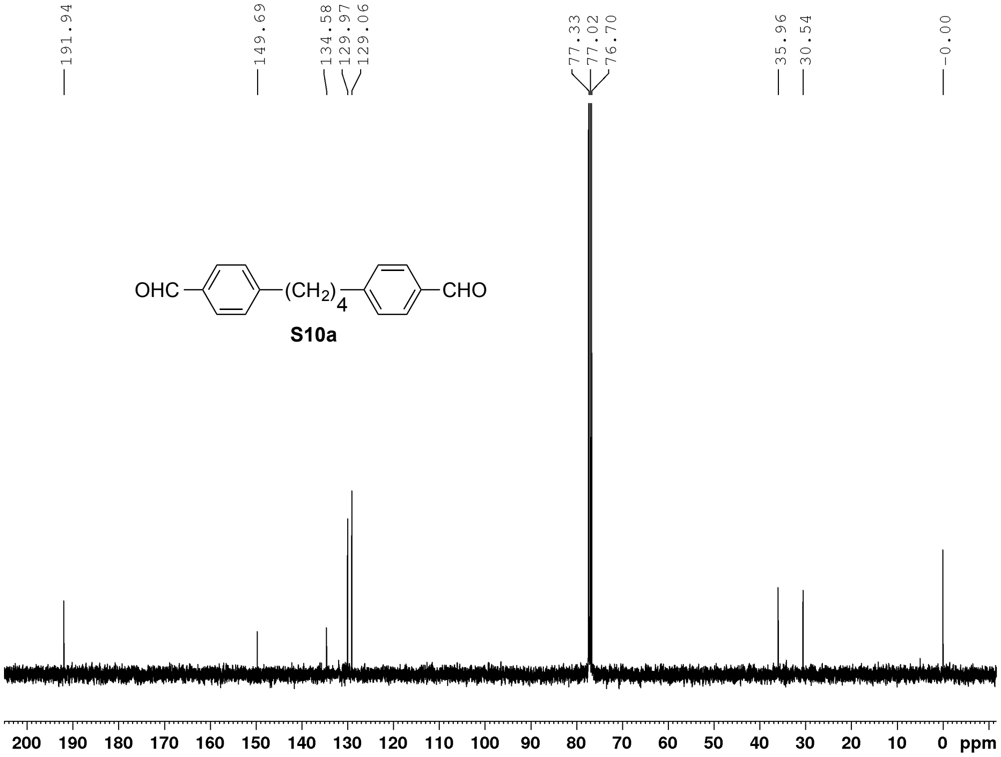

^1H NMR, $CDCl_3$, 400 MHz

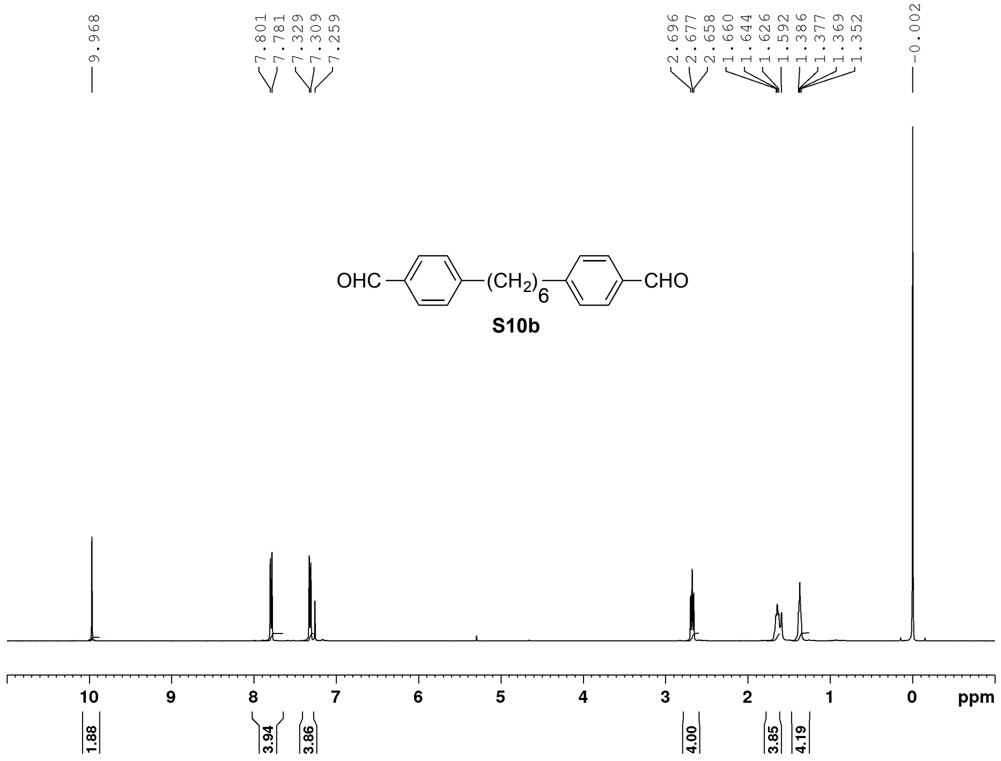

^{13}C NMR, $CDCl_3$, 100 MHz

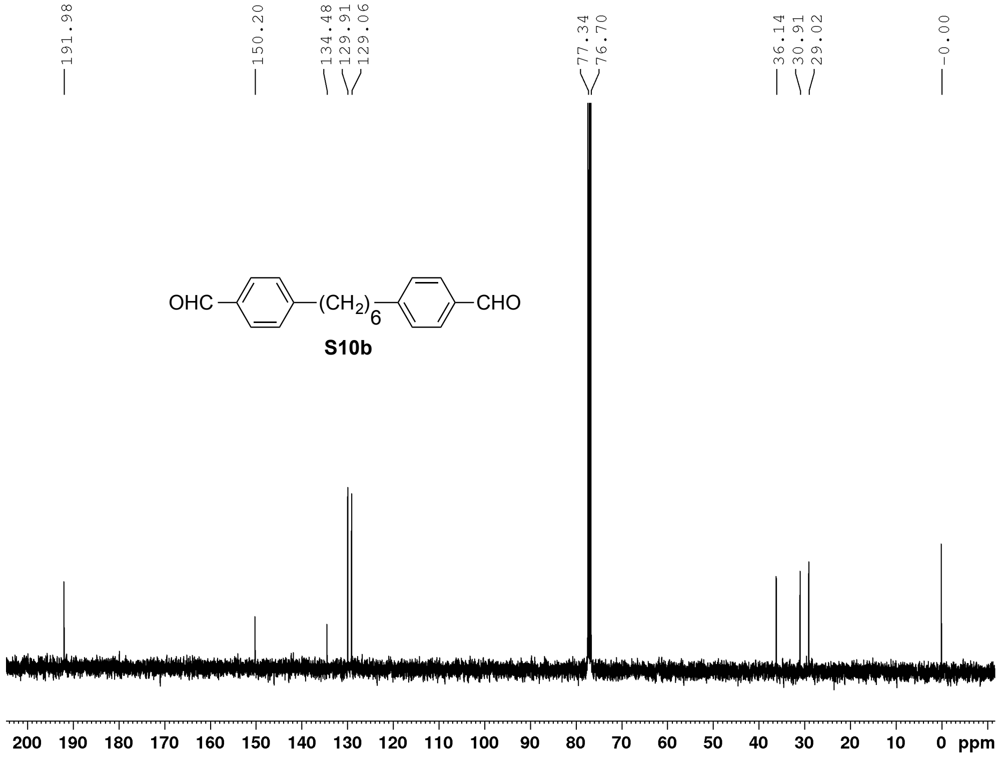

^{1}H NMR, $CDCl_3$, 400 MHz

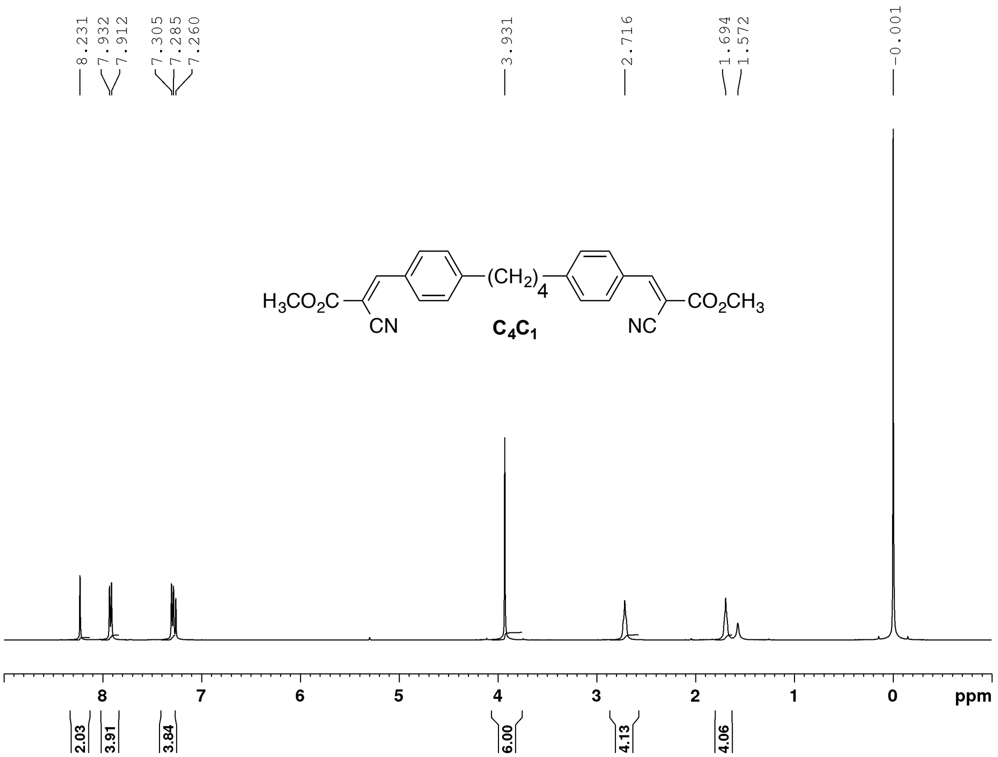

^{13}C NMR, $CDCl_3$, 100 MHz

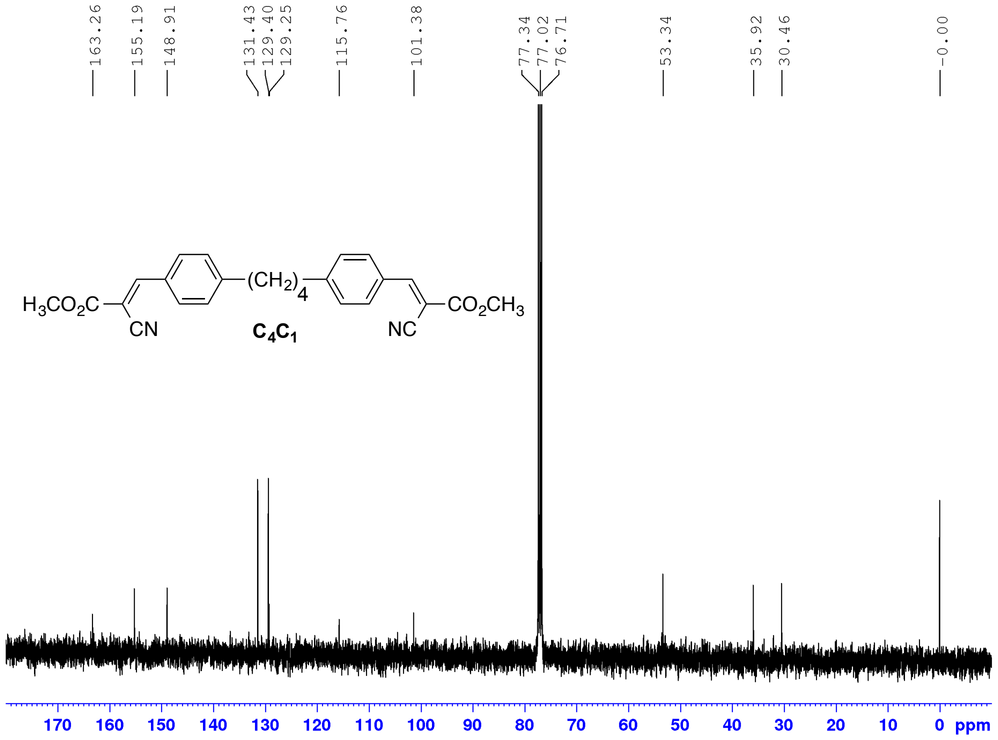

^{1}H NMR, $CDCl_3$, 400 MHz

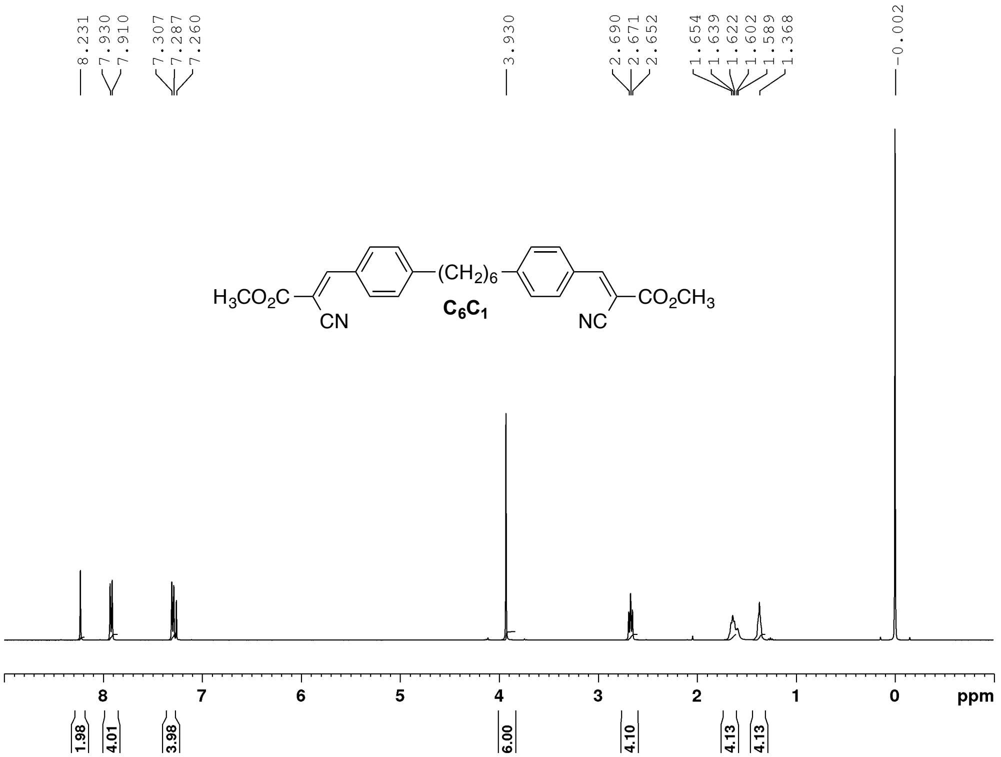

^{13}C NMR, $CDCl_3$, 100 MHz

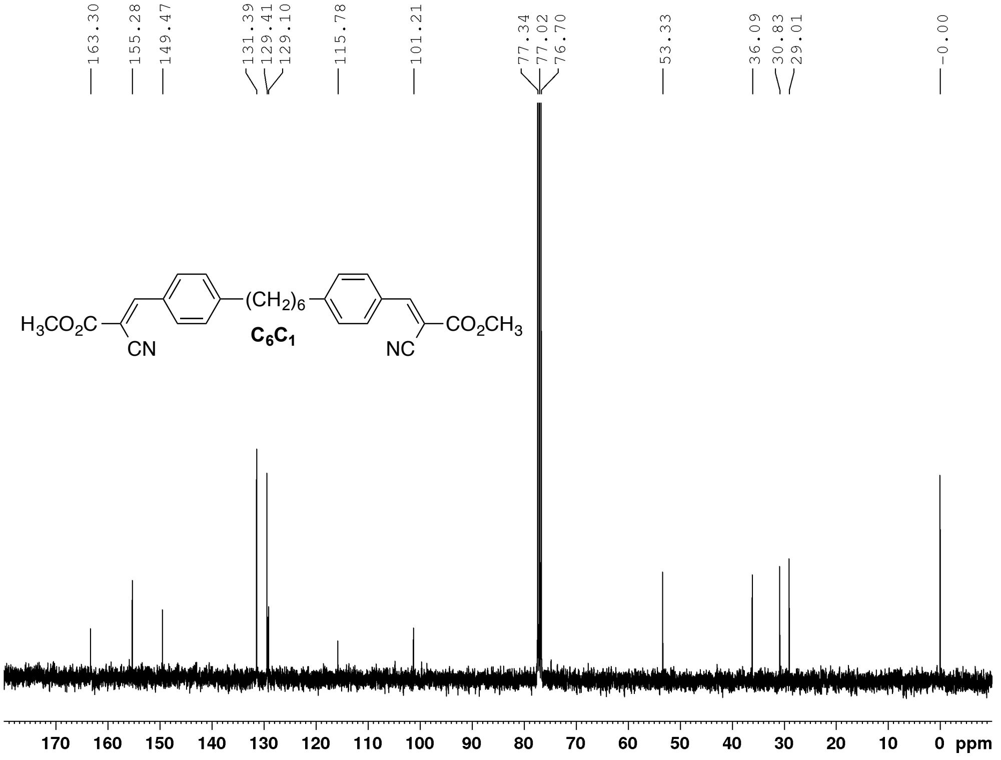

^{1}H NMR, $CDCl_3$, 400 MHz

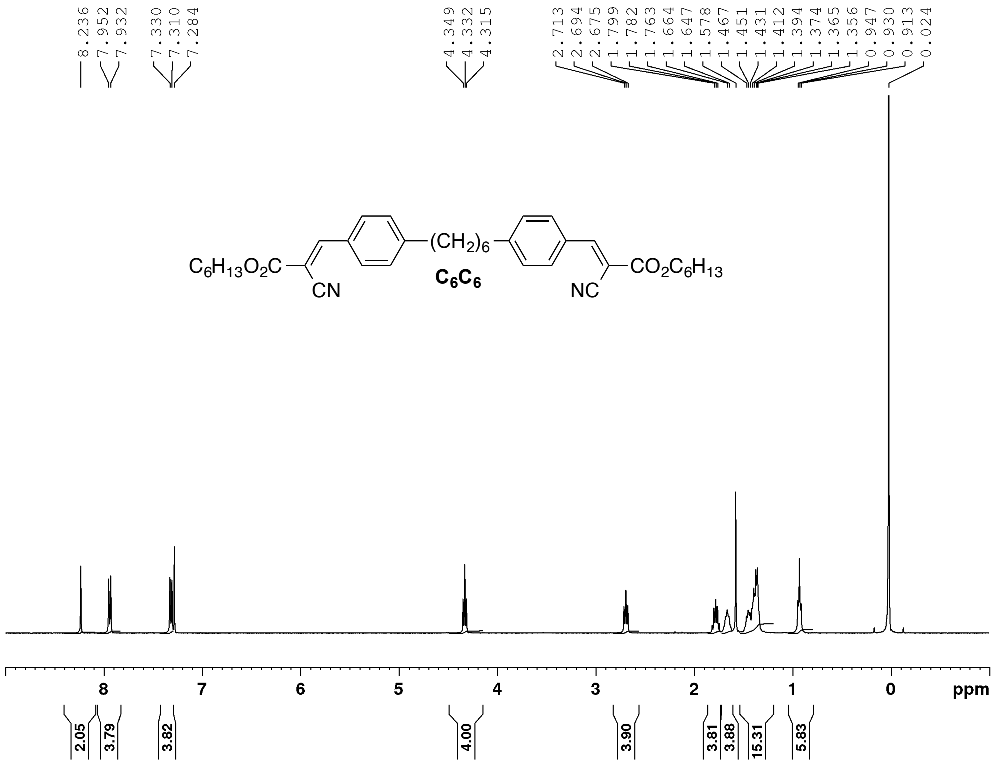

^{13}C NMR, $CDCl_3$, 100 MHz

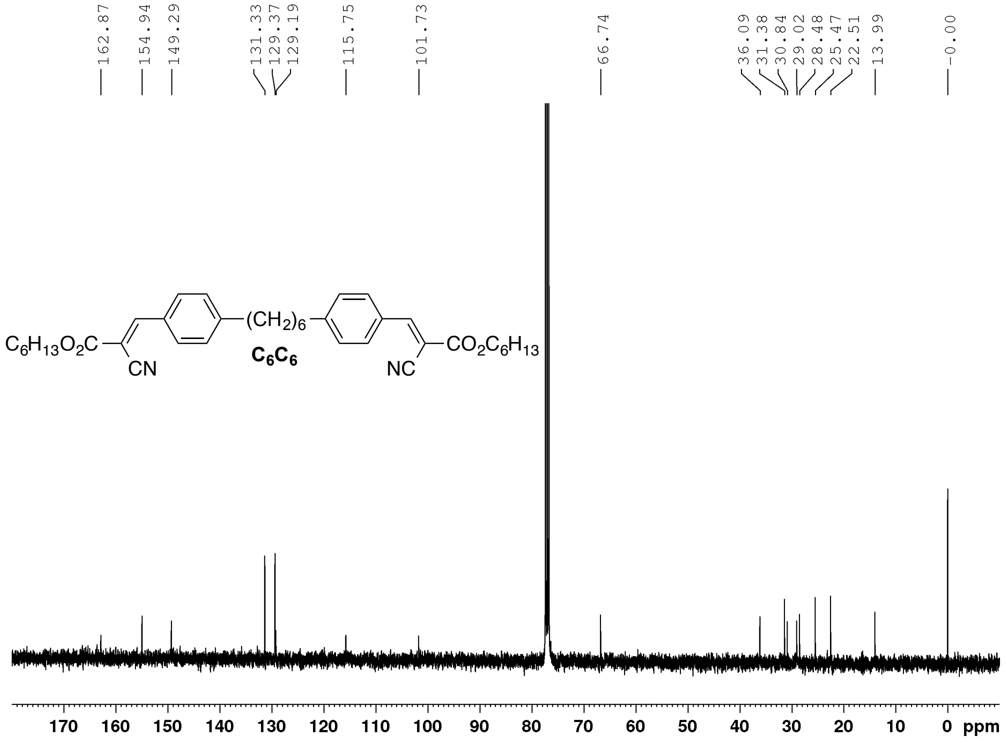

Supplement: Supplementary file 1 [file SC-006-C4SC03274H-s001.pdf]
